# Physiologically Based Pharmacokinetic (PBPK) Modeling of Clopidogrel and Its Four Relevant Metabolites for CYP2B6, CYP2C8, CYP2C19, and CYP3A4 Drug–Drug–Gene Interaction Predictions

**DOI:** 10.3390/pharmaceutics14050915

**Published:** 2022-04-22

**Authors:** Helena Leonie Hanae Loer, Denise Türk, José David Gómez-Mantilla, Dominik Selzer, Thorsten Lehr

**Affiliations:** 1 Department of Clinical Pharmacy, Saarland University, 66123 Saarbrücken, Germany; helena.loer@uni-saarland.de (H.L.H.L.); denise.tuerk@uni-saarland.de (D.T.); dominik.selzer@uni-saarland.de (D.S.); 2 Translational Medicine & Clinical Pharmacology, Boehringer Ingelheim Pharma GmbH & Co. KG, 55216 Ingelheim, Germany; jose_david.gomez_mantilla@boehringer-ingelheim.com

**Keywords:** physiologically based pharmacokinetic (PBPK) modeling, clopidogrel, clopidogrel acyl glucuronide, clopidogrel active metabolite, drug–gene interaction (DGI), drug–drug interaction (DDI), cytochrome P450 2C8 (CYP2C8), cytochrome P450 2C19 (CYP2C19), mechanism-based inactivation, model-informed drug development and discovery (MID3)

## Abstract

The antiplatelet agent clopidogrel is listed by the FDA as a strong clinical index inhibitor of cytochrome P450 (CYP) 2C8 and weak clinical inhibitor of CYP2B6. Moreover, clopidogrel is a substrate of—among others—CYP2C19 and CYP3A4. This work presents the development of a whole-body physiologically based pharmacokinetic (PBPK) model of clopidogrel including the relevant metabolites, clopidogrel carboxylic acid, clopidogrel acyl glucuronide, 2-oxo-clopidogrel, and the active thiol metabolite, with subsequent application for drug–gene interaction (DGI) and drug–drug interaction (DDI) predictions. Model building was performed in PK-Sim^®^ using 66 plasma concentration-time profiles of clopidogrel and its metabolites. The comprehensive parent-metabolite model covers biotransformation via carboxylesterase (CES) 1, CES2, CYP2C19, CYP3A4, and uridine 5′-diphospho-glucuronosyltransferase 2B7. Moreover, CYP2C19 was incorporated for normal, intermediate, and poor metabolizer phenotypes. Good predictive performance of the model was demonstrated for the DGI involving CYP2C19, with 17/19 predicted DGI AUC_last_ and 19/19 predicted DGI C_max_ ratios within 2-fold of their observed values. Furthermore, DDIs involving bupropion, omeprazole, montelukast, pioglitazone, repaglinide, and rifampicin showed 13/13 predicted DDI AUC_last_ and 13/13 predicted DDI C_max_ ratios within 2-fold of their observed ratios. After publication, the model will be made publicly accessible in the Open Systems Pharmacology repository.

## 1. Introduction

The antiplatelet agent clopidogrel is widely used in the prevention of atherothrombotic events, such as secondary prophylaxis after a myocardial infarction or in the event of an acute coronary syndrome [1]. In 2019, it was listed 36th in the outpatient prescription statistics for the United States [2]. Clopidogrel is a prodrug, and the therapeutic effect is due to irreversible binding of its active metabolite to the platelet P2Y12 receptor with subsequent inhibition of adenosine diphosphate (ADP)-induced platelet aggregation [3,4,5].

Clopidogrel is classified as a BCS class II drug [6]. Oral uptake is followed by a rapid absorption coupled with extensive first-pass metabolism [5,7]. After administration of a single dose (SD) of 75 mg clopidogrel, approximately 50% of clopidogrel-related products are detectable in urine and 46% in feces over a 120-h period, indicating an absorption of over 50% of the applied dose [8]. In vitro, clopidogrel has been identified as a substrate of P-glycoprotein (P-gp) [9]. The metabolism of clopidogrel can be divided into two pathways: 85–90% of the absorbed dose is converted via carboxylesterases (CES) into the inactive main metabolite, a carboxylic acid derivative (Clo-COOH), which undergoes further metabolism to clopidogrel acyl glucuronide (Clo-AG) via uridine 5′-diphospho-glucuronosyltransferases (UGTs) [1,10,11,12,13,14]. The remainder is transformed into 2-oxo-clopidogrel (2-Oxo-Clo), with subsequent metabolization to the active metabolite clopidogrel thiol H4 (Clo-AM), both steps via various cytochrome P450 (CYP) enzymes [15].

A high interindividual variability in plasma levels of clopidogrel and the effect on platelet aggregation can be observed, resulting mainly from the so-called “clopidogrel resistance”. Not well defined, this term is generally associated with a considerable attenuation of the inhibitory effect on platelet aggregation, with the specific mechanisms not yet conclusively investigated [16]. Genetic polymorphisms have been proposed as a possible cause, particularly of the *CYP2C19* gene, resulting in rapid (RM), normal (NM), intermediate (IM), or poor metabolizer (PM) phenotypes [16,17,18]. About 3% of Caucasians exhibit a CYP2C19 PM phenotype, whereas the frequency is much higher in Asians, e.g., about 14% among Chinese, making the drug–gene interactions (DGIs) clinically relevant [19]. In 2010, the United States Food and Drug Administration (FDA) added a boxed warning to the label of clopidogrel to specifically draw attention to its impaired efficacy in PMs of CYP2C19 [20].

Furthermore, drug–drug interactions (DDIs) are suspected to play a role with clopidogrel as the victim, especially in association with CYP3A4 and CYP2C19 interacting perpetrators [17,21]. While inhibitors (e.g., omeprazole, fluoxetine, grapefruit juice, ketoconazole) are reported to significantly decrease plasma levels of Clo-AM, inducers (e.g., rifampicin) have shown the opposite effect [22,23,24,25,26,27]. For example, concomitant intake of the proton pump inhibitor omeprazole and clopidogrel over several days decreases the area under the plasma concentration-time curve (AUC) of Clo-AM by approximately half, whereas pretreatment with the antibiotic agent rifampicin increases the AUC of Clo-AM by 4-fold [25,27]. However, clopidogrel does not only act as a victim but also as a perpetrator. The FDA lists clopidogrel as a strong clinical index inhibitor of CYP2C8 (specifically Clo-AG) and weak clinical inhibitor of CYP2B6 [28]. DDI studies conducted with various substrates of CYP2C8 (e.g., montelukast, pioglitazone, repaglinide, dasabuvir, desloratadine, selexipag) confirmed Clo-AG to be a potent, mechanism-based inactivator of CYP2C8 [29,30,31,32,33,34], increasing for instance the AUC of the antidiabetic agent repaglinide by 3.9-fold following a 75 mg maintenance dose (MD) of clopidogrel, potentially contributing to the risk of adverse events [33]. Moreover, clopidogrel proved to be the most potent CYP2B6 inhibitor (mechanism-based) out of 227 drugs analyzed [35]. Following pretreatment with 75 mg clopidogrel daily and subsequent intake of the antidepressant/smoking cessation agent bupropion, the AUC of its metabolite hydroxybupropion was reduced by 52% [36]. While DDIs and DGIs are usually investigated separately in studies, real-life occurrence of drug–drug–gene interactions (DDGIs) is rarely analyzed.

To ensure effective therapy of clopidogrel and concurrent drugs while maintaining adequate control of adverse events, the complex pharmacokinetics (PK) of clopidogrel and its metabolites, characterized by their high potential for DDIs, DGIs, and DDGIs, should be thoroughly investigated. Physiologically based pharmacokinetic (PBPK) modeling has been a capable tool for such approaches for many years, as it allows the study of complex metabolic processes and pharmacokinetic aspects. Where relevant, different genotypes as well as interactions involving multiple drugs can be considered [37,38]. Additionally, it is well recognized for model-informed drug development and discovery (MID3). In recent years, an increasing number of applications have been submitted to regulatory authorities containing PBPK models addressing different research questions, about two-thirds of which were related to DDIs [39].

The objectives of the present work were (a) the development of a whole-body PBPK model of clopidogrel including all relevant metabolites (Clo-COOH, Clo-AG, 2-Oxo-Clo, Clo-AM) as well as the application of the model to predict (b) the DGI involving CYP2C19, along with (c) DDIs involving CYP2B6, CYP2C19, CYP3A4, and specifically CYP2C8. Once published, the model will be made publicly accessible in the Open Systems Pharmacology (OSP) repository. The Supplementary Materials provide a detailed documentation regarding the development and evaluation of the model.

## 2. Materials and Methods

### 2.1. Software

PBPK model development, parameter optimization (Levenberg–Marquardt algorithm), and local sensitivity analysis were accomplished using PK-Sim^®^ and MoBi^®^ (Open Systems Pharmacology Suite 9.1, www.open-systems-pharmacology.org, 2020). Digitization of published clinical study data was carried out via Engauge Digitizer 12.1 (M. Mitchell [40], 2019) according to Wojtyniak et al. [41]. Calculation of pharmacokinetic parameters, creation of plots along with model performance measurements were performed with the R programming language version 4.1.1 (The R Foundation for Statistical Computing, Vienna, Austria, 2021) and Rstudio 1.4.1717 (RStudio Inc., Boston, MA, USA, 2021).

### 2.2. Clinical Study Data

Plasma concentration-time profiles of clopidogrel and its four metabolites (Clo-COOH, Clo-AG, 2-Oxo-Clo, Clo-AM) were digitized from published literature. All studies were conducted in healthy participants covering a wide dosing range, SD and MD, intravenous and peroral administration. Considering the digitized studies, training [23,42,43,44,45,46,47,48,49,50,51,52] and test datasets [42,44,53,54,55,56,57,58,59,60,61,62,63,64,65,66,67,68,69,70,71,72] were defined for development and evaluation of the clopidogrel model, respectively. The training dataset preferably included profiles reporting many measurement points over a long period of time and studies quantifying multiple metabolites of interest. A comprehensive list of all profiles utilized can be found in Table S2 of the Supplementary Materials.

### 2.3. PBPK Model Building

Development of the clopidogrel parent-metabolite model was initiated with an extensive literature search for physicochemical properties as well as information regarding absorption, distribution, metabolism, and excretion (ADME) processes of clopidogrel and its metabolites Clo-COOH, Clo-AG, 2-Oxo-Clo, and Clo-AM.

A virtual individual was created for each included study. If available, mean and mode data on age, sex, weight, height, body mass index, and ethnicity from the respective study were incorporated. If demographic data were missing, a default individual was generated based on data for different ethnicities provided in PK-Sim^®^. Relative expression for enzymes and transporters of interest in the different organs were specified according to the PK-Sim^®^ expression database [73]. Further information on the respective expression is available in Table S1 of the Supplementary Materials.

During model development, model input parameters missing from the literature or involved in calculation methods of PK-Sim^®^ were fitted using the training dataset. Due to the complexity of the model, a stepwise approach was applied. First, only clopidogrel parameters were optimized to establish the accurate ratio between the two major pathways. Next, separate optimizations were performed for parameters related to the pathways leading to the formation of Clo-AG (Clo-COOH, Clo-AG) and Clo-AM (2-Oxo-Clo, Clo-AM), respectively. Since many enzymes inhibited by clopidogrel are responsible for its own biotransformation, relevant inhibition parameters of clopidogrel and its metabolites were incorporated during model development. Studies quantifying clopidogrel and Clo-AM in CYP2C19 PMs were used to define CYP2C19 independent metabolism [48,51]. An overview of clopidogrel’s metabolism, specifically the processes implemented in PK-Sim^®^, can be found in Section 3.1.

### 2.4. PBPK Model Evaluation

The clopidogrel parent-metabolite model was evaluated both graphically and quantitatively. Predicted concentration-time profiles were plotted alongside their respective observed data points for visual comparison. Furthermore, goodness-of-fit (GOF) plots were used to display the agreement between predicted and observed concentration values. GOF plots were additionally generated to compare the calculated area under the concentration-time curve between first and last concentration measurements (AUC_last_) and maximum plasma concentration (C_max_) values for all predicted versus observed profiles. Predictions within the 2-fold deviation of observed values were considered successful. Quantitative evaluations were performed by calculating mean relative deviations (MRDs) for all plasma concentration predictions along with geometric mean fold errors (GMFEs) for all predicted AUC_last_ and C_max_ values according to Equations (1) and (2), respectively.

(1) $${\mathrm{MRD}=10}^{x};\;x=\sqrt{\frac{\sum_{i=1}^{k}{\;(\log}_{10}\hat{c_{i}}{\;-\;\log}_{10}c_{i}{)}^{2}}{k}}$$

where $c_{i}$ = i-th observed plasma concentration, $\hat{c_{i}}$ = predicted plasma concentration corresponding to the i-th observed plasma concentration, and k = number of observed values.

(2) $${\mathrm{GMFE}=10}^{x};\;x=\frac{\sum_{i=1}^{m}\;\left|\log_{10}\;\left(\frac{\hat{p_{i}}}{p_{i}}\right)\right|\;}{m}$$

where $p_{i}$ = observed AUC_last_ or C_max_ value of study i, $\hat{p_{i}}$ = corresponding predicted AUC_last_ or C_max_ value of study i, and m = number of studies.

Finally, a local sensitivity analysis was conducted, described fully in Section S2.7.1 of the Supplementary Materials.

### 2.5. DGI Modeling

Metabolism via CYP2C19 was implemented according to Michaelis–Menten kinetics. Different levels of CYP2C19 activity between phenotypes were modeled by maintaining the Michaelis–Menten constant (K_M_) unchanged while varying the catalytic rate constant (k_cat_) depending on the phenotype. For CYP2C19 NMs, activity was assumed to be 100%, for IMs 50%, and for PMs 0%, based on reported activity scores [18,74] (see Table 1). CYP2C19 NM phenotype was assumed when no genotyping had been conducted during studies.

DGI model evaluation was performed by plotting and comparing predicted to observed plasma concentration-time profiles of each phenotype. Predicted DGI AUC_last_ and C_max_ ratios were calculated for IMs and PMs, both in relation to NMs (Equation (3)), with subsequent comparison to corresponding observed ratios computed analogously.

(3) $$\mathrm{DGI}\;\mathrm{PK}\;\mathrm{parameter}\;\mathrm{ratio}=\;\frac{\mathrm{PK}\;{\mathrm{parameter}}_{\mathrm{DGI}}}{\mathrm{PK}\;{\mathrm{parameter}}_{\mathrm{Reference}}}$$

where PK parameter = AUC_last_ or C_max_, PK parameter_DGI_ = AUC_last_ or C_max_ of IM or PM phenotype, and PK parameter_Reference_ = AUC_last_ or C_max_ of NM phenotype.

The limits proposed by Guest et al. [75] were applied to evaluate prediction accuracy (including 20% variability to account for uncertainties in observed ratios). Quantitative assessment was performed by calculating GMFE values for all predicted DGI AUC_last_ and C_max_ ratios according to Equation (2).

### 2.6. DDI Network Modeling

DDI performance of the clopidogrel model was assessed by building a CYP2B6/CYP2C8/CYP2C19/CYP3A4 DDI network centered around clopidogrel, coupling the clopidogrel model with publicly accessible models of bupropion, montelukast, omeprazole, pioglitazone, repaglinide, and rifampicin [76,77,78,79,80] by incorporating relevant interaction parameters. DDI partners were selected if listed by the FDA as clinical (index) substrates/inhibitors/inducers of CYP enzymes relevant for clopidogrel and recommended for use in clinical DDI studies [28]. The different types of interaction implemented, i.e., induction, competitive inhibition, and mechanism-based inactivation, are described in Section S4.1 of the Supplementary Materials. The clopidogrel–repaglinide DDI was included in the training dataset to inform the intrahepatic Clo-AG concentration required for sufficient CYP2C8 inhibition [33].

Evaluating the DDI network, corresponding predicted as well as observed plasma concentration-time profiles of the victim drug administered with and without the respective perpetrator drug were plotted and compared graphically. Moreover, DDI AUC_last_ and C_max_ ratios were calculated for every predicted and observed profile according to Equation (4), with subsequent comparison, applying the limits proposed by Guest et al. [75] to determine prediction accuracy (including 20% variability).

(4) $$\mathrm{DDI}\;\mathrm{PK}\;\mathrm{parameter}\;\mathrm{ratio}=\;\frac{\mathrm{PK}\;{\mathrm{parameter}}_{\mathrm{DDI}}}{\mathrm{PK}\;{\mathrm{parameter}}_{\mathrm{Control}}\;}$$

where PK parameter = AUC_last_ or C_max_, PK parameter_DDI_ = AUC_last_ or C_max_ of victim drug during perpetrator co-administration, and PK parameter_Control_ = AUC_last_ or C_max_ of victim drug control.

Quantitative assessment was performed by calculating GMFE values for all predicted DDI AUC_last_ and C_max_ ratios according to Equation (2).

## 3. Results

### 3.1. PBPK Model Building and Evaluation

Building and evaluation of the clopidogrel parent-metabolite model was performed using one clinical study involving intravenous application of 0.1–300 mg clopidogrel (SD) as well as 31 studies administering clopidogrel perorally at common doses of 75 to 600 mg (SD and MD), providing a total of 23 clopidogrel, 21 Clo-COOH, 3 Clo-AG, 1 2-Oxo-Clo, and 18 Clo-AM plasma concentration-time profiles. Information on all profiles utilized is listed in Table S2 of the Supplementary Materials.

**Figure 1:**
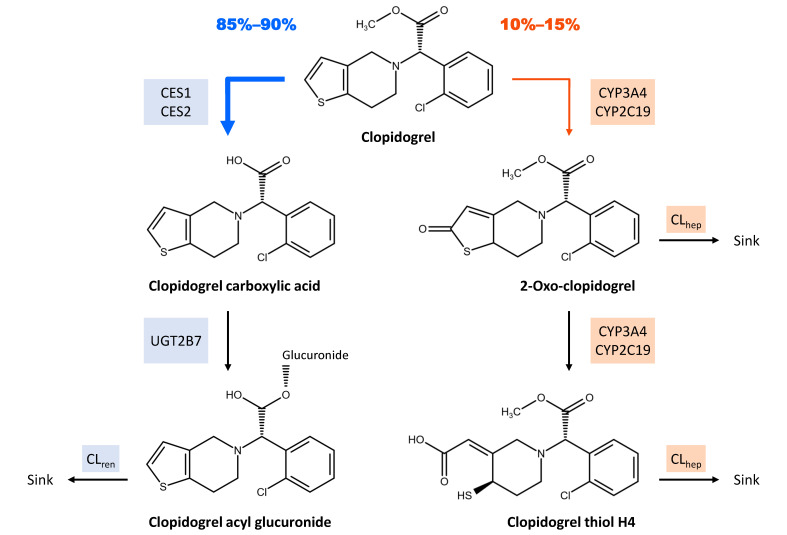
Clopidogrel’s main metabolic pathways and excretion processes implemented in PK-Sim^®^. In total, 85–90% of the absorbed dose is converted via CES1 and CES2 into clopidogrel carboxylic acid, which undergoes further metabolism to clopidogrel acyl glucuronide via UGT2B7, with the latter being excreted via a nonspecific renal clearance. The remainder of clopidogrel absorbed is transformed via CYP3A4 and CYP2C19 into 2-oxo-clopidogrel with subsequent metabolization to the active metabolite clopidogrel thiol H4, both metabolites being excreted via nonspecific hepatic clearances. CES: carboxylesterase, CL_hep_: hepatic clearance, CL_ren_: renal clearance, CYP: cytochrome P450, UGT: uridine 5′-diphospho-glucuronosyltransferase. Metabolism and excretion steps are represented by arrows.

Figure 1 depicts a schematic overview of the implemented metabolic pathways and excretion processes. Within the pathway leading to Clo-AG, metabolism from clopidogrel to Clo-COOH was incorporated via CES1 and CES2, with CES2 expression limited to the intestine. UGT2B7 was integrated for glucuronidation of Clo-COOH with subsequent elimination of the resulting Clo-AG via a nonspecific renal clearance. Within the second main pathway leading to the formation of Clo-AM, CYP2C19 and CYP3A4 were incorporated for the oxidation of clopidogrel to 2-Oxo-Clo and further metabolism to Clo-AM. Additional transformation of 2-Oxo-Clo into various inactive thiol metabolites and the elimination of Clo-AM were implemented via nonspecific hepatic clearance processes.

For each biotransformation process, K_M_ values were adopted from the literature, while k_cat_ and nonspecific clearance parameters were fitted. Moreover, all (auto)inhibition parameters of clopidogrel and its metabolites were implemented as literature values. A full list of model parameters of clopidogrel and its metabolites is provided in Section S1.3 of the Supplementary Materials.

**Figure 2:**
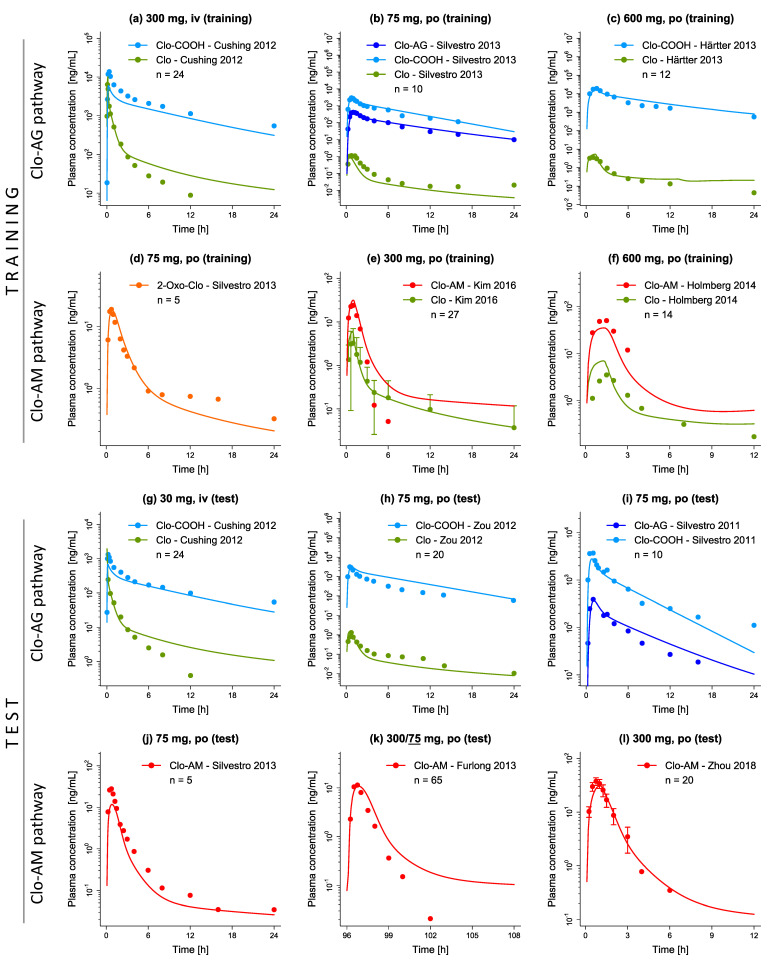
Representative plots of predicted plasma concentration-time profiles of clopidogrel and its metabolites. Split up according to (**a**–**f**) training and (**g**–**l**) test datasets, solid lines represent the model predictions, while corresponding observed data are shown as symbols (±standard deviation, if available) [23,42,44,46,47,54,59,62,72]. Detailed information on all profiles and clinical studies, can be found in Table S2 of the Supplementary Materials. 2-Oxo-Clo: 2-oxo-clopidogrel, Clo: clopidogrel, Clo-AM: clopidogrel thiol H4, Clo-AG: clopidogrel acyl glucuronide, Clo-COOH: clopidogrel carboxylic acid, iv: intravenous, n: number of participants, po: peroral.

The final clopidogrel parent-metabolite model allows good description (training dataset) and prediction (test dataset) of clopidogrel, Clo-COOH, Clo-AG, 2-Oxo-Clo, and Clo-AM plasma concentration-time profiles following intravenous and peroral clopidogrel administration. Figure 2 shows a representative sample of plasma concentration-time profiles from the training and test dataset. All predicted and observed plasma concentration-time profiles are depicted in Sections S2.1 and S2.2 of the Supplementary Materials as semilogarithmic and linear plots.

**Figure 3:**
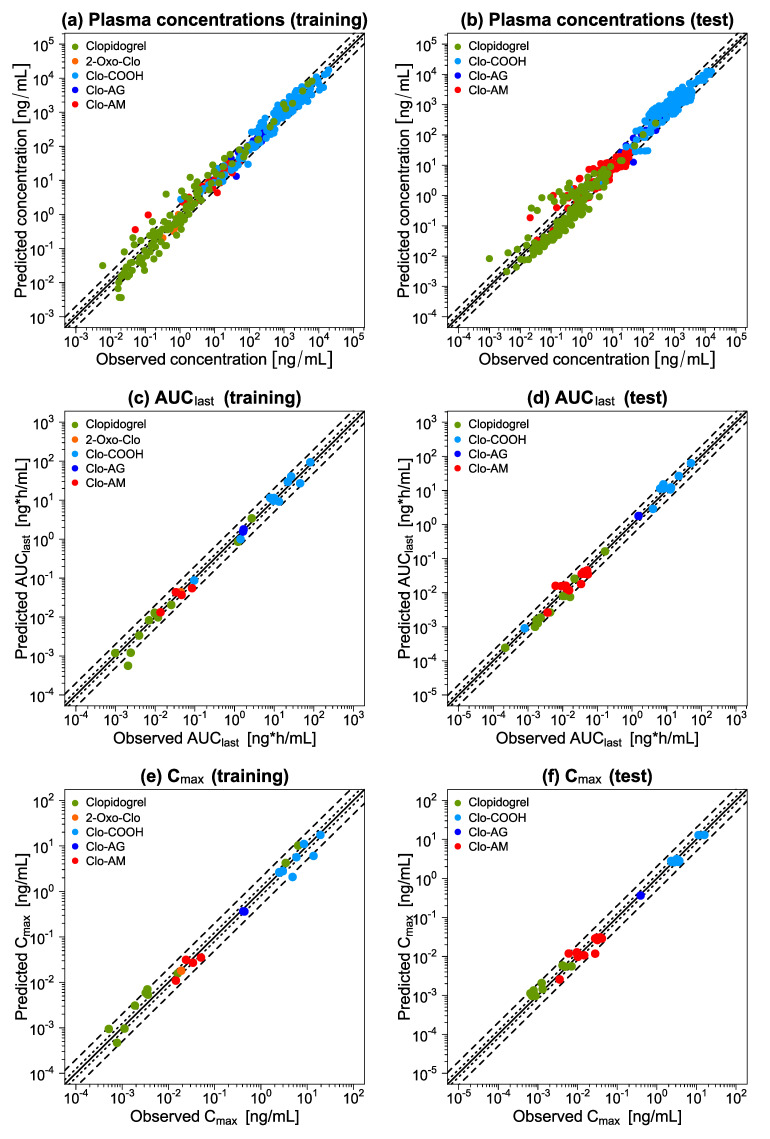
Goodness-of-fit plots of the final clopidogrel parent-metabolite model. Split into training and test datasets, (**a**,**b**) each predicted plasma concentration as well as predicted (**c**,**d**) AUC_last_ and (**e**,**f**) C_max_ values are plotted against their corresponding observed values. The solid line represents the line of identity, while dotted lines indicate 1.25-fold and dashed lines 2-fold deviation from the respective observed value. Detailed information on all profiles and clinical studies can be found in Table S2 of the Supplementary Materials. 2-Oxo-Clo: 2-oxo-clopidogrel, AUC_last_: area under the plasma concentration-time curve determined between first and last concentration measurements, Clo-AM: clopidogrel thiol H4, Clo-AG: clopidogrel acyl glucuronide, Clo-COOH: clopidogrel carboxylic acid, C_max_: maximum plasma concentration.

Figure 3 displays GOF plots for all concentration measurements as well as for all AUC_last_ and C_max_ values, divided into training and test datasets. Considering both datasets for all five compounds, 76% of the predicted concentration measurements, 63/66 predicted AUC_last_, and 55/58 predicted C_max_ values lie within the 2-fold range of their respective observed counterparts. In total, the model shows a mean MRD of 1.91 as well as mean GMFE_AUClast_ and GMFE_Cmax_ values of 1.38 and 1.35, respectively, thus, confirming its adequate descriptive and predictive performance. Individual MRD and GMFE values for all profiles are listed in Tables S5 and S6 of the Supplementary Materials.

Sensitivity analyses of an MD simulation over ten days with the administration of 75 mg clopidogrel daily revealed the AUC_last_ of clopidogrel, 2-Oxo-Clo, and Clo-AM to be most sensitive to perturbation of the respective optimized lipophilicity, while the AUC_last_ of Clo-COOH and Clo-AG showed the highest sensitivity to perturbation of the optimized k_cat_ of UGT2B7 and fraction unbound adopted from the literature, respectively. The complete quantitative assessment of the sensitivity analyses, along with a list of all parameters evaluated for their influence on AUC_last_ are provided in Section S2.7.2 of the Supplementary Materials.

### 3.2. DGI Modeling

The DGI model was developed using five DGI studies, with one study quantifying clopidogrel, three studies measuring Clo-AM, and one study investigating both compounds in relation to different phenotypes [48,51,71,81,82]. All studies utilized are listed in Table S8 of the Supplementary Materials. During the studies, *CYP2C19* genotyping was conducted for alleles *1, *2, and *3 with subsequent assignment of genotypes to phenotypes NM, IM, and PM according to Table 1.

**Figure 4:**
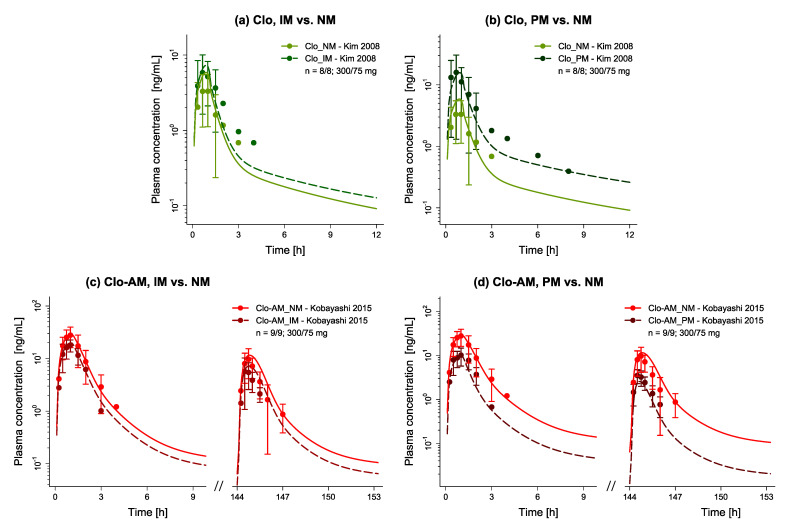

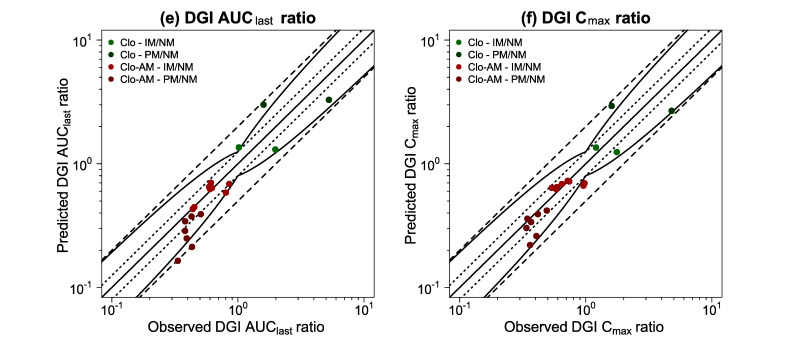
Drug–gene interaction model evaluation. Presented are examples of predicted plasma concentration-time profiles of (**a**,**b**) clopidogrel and (**c**,**d**) Clo-AM for IM and PM phenotypes compared separately to NM phenotypes, alongside corresponding observed data [48,51]. Dashed (IM or PM) and solid (NM) lines represent the model predictions, while corresponding observed data are shown as symbols (±standard deviation, if available). Predicted versus observed (**e**) DGI AUC_last_ and (**f**) DGI C_max_ ratios are shown with the solid line representing the line of identity, dotted lines indicating 1.25-fold, and dashed lines 2-fold deviation from the respective observed value, along with the curved lines marking the prediction success limits proposed by Guest et al. [75] (including 20% variability to account for uncertainties in observed ratios). Detailed information on all DGI studies as well as individual DGI AUC_last_ and DGI C_max_ ratios are provided in Tables S8 and S9 of the Supplementary Materials. AUC_last_: area under the plasma concentration-time curve determined between first and last concentration measurements, Clo: clopidogrel, Clo-AM: clopidogrel thiol H4, C_max_: maximum plasma concentration, DGI: drug–gene interaction, IM: cytochrome P450 2C19 intermediate metabolizer, n: number of participants, NM: cytochrome P450 2C19 normal metabolizer, PM: cytochrome P450 2C19 poor metabolizer.

Figure 4 presents examples of predicted versus observed plasma concentration-time profiles for (a–b) clopidogrel and (c–d) Clo-AM divided by phenotype, along with (e–f) predicted versus observed DGI AUC_last_ and C_max_ ratios for each profile. In total, 15/19 predicted DGI AUC_last_ and 16/19 predicted DGI C_max_ ratios fall within the limits proposed by Guest et al. [75], with low mean GMFE values of 1.36 and 1.27, respectively, thus, indicating a good performance of the model regarding CYP2C19 DGI predictions. Individual GMFE values for all DGI profiles are provided in Table S9 of the Supplementary Materials along with all predicted versus observed plasma concentration-time profiles as semilogarithmic and linear plots in Sections S3.2 and S3.3 of the Supplementary Materials.

### 3.3. DDI Network Modeling

**Figure 5:**
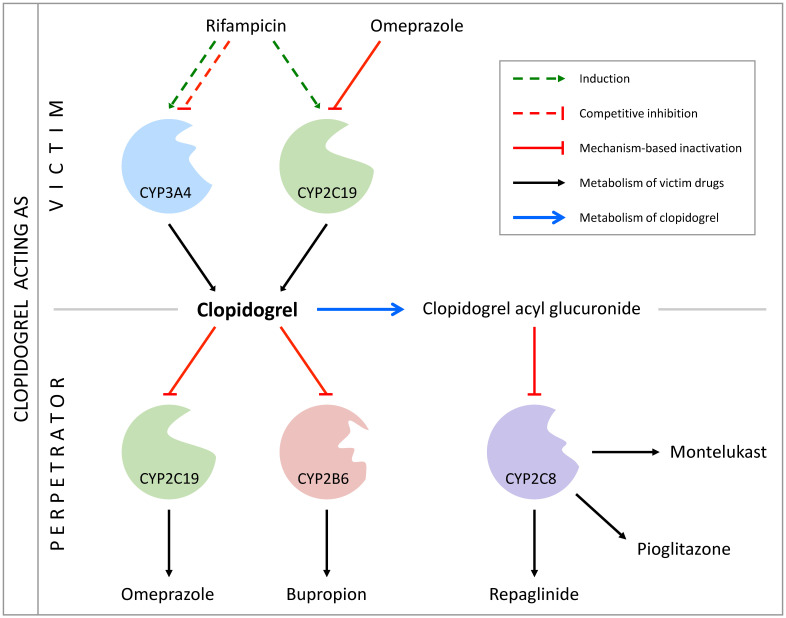
Schematic overview of the modeled drug–drug interaction network. For brevity, only the respective main interaction processes are shown. While clopidogrel acts as victim in the context of drug–drug interactions with omeprazole and rifampicin, it represents the perpetrator when administered concomitantly with bupropion and omeprazole. As stated by the United States Food and Drug Administration, in particular, clopidogrel acyl glucuronide acts as a perpetrator regarding CYP2C8 [28]. Montelukast, pioglitazone, and repaglinide were included in the drug–drug interaction network as CYP2C8 substrates. Table S11 of the Supplementary Materials contains a list of all implemented interaction processes per drug–drug interaction. CYP: cytochrome P450.

The DDI network centered around clopidogrel was built and evaluated using nine DDI studies. Regarding clopidogrel as the victim, two studies examined the impact of the CYP2C19 mechanism-based inactivator omeprazole on the PK of Clo-AM when co-administered with clopidogrel for several days [25,26], while one study addressed the effect of pretreatment with the CYP2C19 inducer/CYP3A4 competitive inhibitor and inducer rifampicin on Clo-AM [27]. With respect to clopidogrel as the perpetrator, two studies investigated clopidogrel’s pretreatment influence as the mechanism-based inactivator of CYP2B6 and CYP2C19. One study evaluated the effect on the PK of (hydroxy)bupropion and one on omeprazole [36,83]. Moreover, the impact on montelukast, pioglitazone, and repaglinide due to the mechanism-based inactivation of CYP2C8 by Clo-AG was assessed in three separate studies over several days of clopidogrel intake [31,32,33]. Figure 5 provides a schematic overview of the modeled DDI network, focusing on the respective main interaction processes. Table S11 of the Supplementary Materials contains a list of all implemented interaction processes per DDI. Extended information on DDI studies, including dosing regimens and subject data, as well as FDA classification by clinical (index) substrate/inhibitor/inducer and model parameters of the DDI partners can be found in Sections S4.2–S4.4 of the Supplementary Materials.

**Figure 6:**
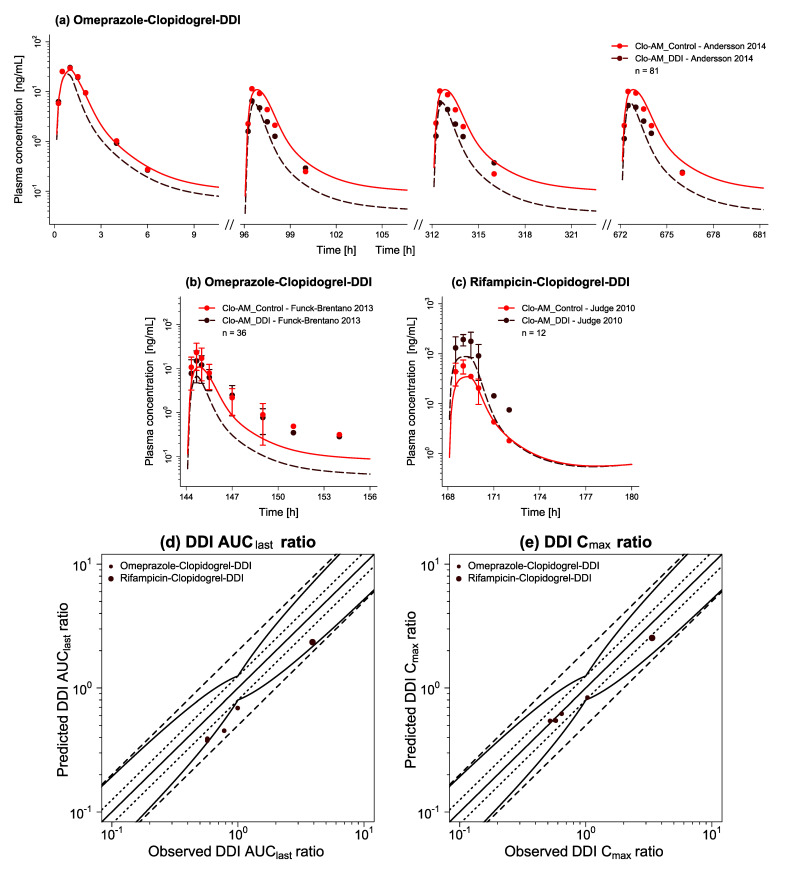
Drug–drug interaction network evaluation with clopidogrel as victim. Presented are predicted plasma concentration-time profiles of Clo-AM with (DDI) and without (Control) intake of the respective perpetrator drug ((**a**,**b**) omeprazole, (**c**) rifampicin), alongside corresponding observed data [25,26,27]. Dashed (DDI) and solid (Control) lines represent the model predictions, while corresponding observed data are shown as symbols (±standard deviation, if available). Predicted versus observed (**d**) DDI AUC_last_ and (**e**) DDI C_max_ ratios are shown with the solid line representing the line of identity, dotted lines indicating 1.25-fold, and dashed lines 2-fold deviation from the respective observed value, along with the curved lines marking the prediction success limits proposed by Guest et al. [75] (including 20% variability to account for uncertainties in observed ratios). Detailed information on all DDI studies as well as individual DDI AUC_last_ and DDI C_max_ ratios are provided in Tables S10 and S19 of the Supplementary Materials. AUC_last_: area under the plasma concentration-time curve determined between first and last concentration measurements, Clo-AM: clopidogrel thiol H4, C_max_: maximum plasma concentration, DDI: drug–drug interaction, n: number of participants.

**Figure 7:**
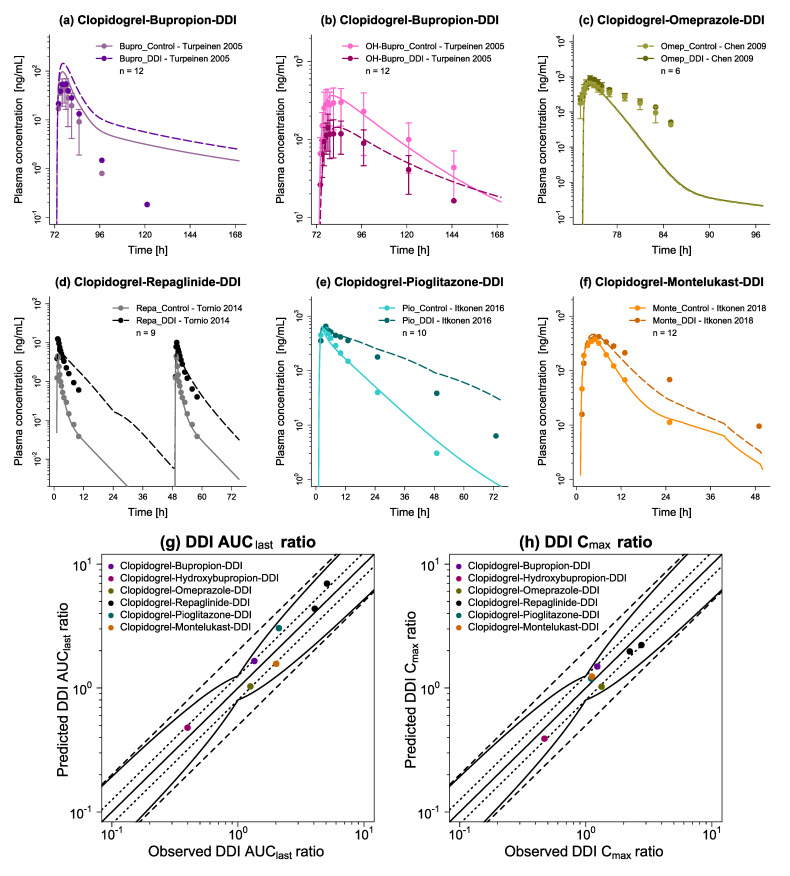
Drug–drug interaction network evaluation with clopidogrel as perpetrator. Presented are predicted plasma concentration-time profiles of the respective victim with (DDI) and without (Control) intake of clopidogrel ((**a**) bupropion, (**b**) hydroxybupropion, (**c**) omeprazole, (**d**) repaglinide, (**e**) pioglitazone, (**f**) montelukast), alongside corresponding observed data [31,32,33,36,83]. Dashed (DDI) and solid (Control) lines represent the model predictions, while corresponding observed data are shown as symbols (±standard deviation, if available). Predicted versus observed (**g**) DDI AUC_last_ and (**h**) DDI C_max_ ratios are shown with the solid line representing the line of identity, dotted lines indicating 1.25-fold, and dashed lines 2-fold deviation from the respective observed value, along with the curved lines marking the prediction success limits proposed by Guest et al. [75] (including 20% variability to account for uncertainties in observed ratios). Detailed information on all DDI studies as well as individual DDI AUC_last_ and DDI C_max_ ratios are provided in Tables S10 and S19 of the Supplementary Materials. AUC_last_: area under the plasma concentration-time curve determined between first and last concentration measurements, bupro: bupropion, C_max_: maximum plasma concentration, DDI: drug–drug interaction, Monte: montelukast, n: number of participants, OH-Bupro: hydroxybupropion, Omep: omeprazole, Pio: pioglitazone, Repa: repaglinide.

Classified according to whether clopidogrel represents the victim or perpetrator, Figure 6a–c and Figure 7a–f illustrate predicted versus observed victim plasma concentration-time profiles with and without intake of the respective perpetrator, while Figure 6d,e and 7g,h display predicted versus observed DDI AUC_last_ and C_max_ ratios for each profile. Overall, 11/13 predicted DDI AUC_last_ and 13/13 predicted DDI C_max_ ratios lie within the limits proposed by Guest et al. [75], while all ratios fall within the 2-fold deviation from their respective observed values. Furthermore, low mean GMFE values for predicted DDI AUC_last_ ratios (1.39) and for predicted DDI C_max_ ratios (1.15) demonstrate a good DDI prediction performance. Individual GMFE values for all DDI profiles are available in Table S19 of the Supplementary Materials along with all predicted versus observed plasma concentration-time profiles as semilogarithmic and linear plots in Section S4.5 and S4.6 of the Supplementary Materials.

## 4. Discussion

In this work, a clopidogrel parent-metabolite whole-body PBPK model was successfully built and evaluated, capable of adequately describing and predicting plasma concentrations of clopidogrel and its metabolites Clo-COOH, Clo-AG, 2-Oxo-Clo, and Clo-AM over a wide dosing range of intravenously (0.1–300 mg, SD) and perorally (75–600 mg, SD and MD) administered clopidogrel. Moreover, the application of the model allowed the successful prediction of the DGI involving CYP2C19, along with DDIs involving CYP2B6, CYP2C8, CYP2C19, and CYP3A4.

Predicted intestinal absorption ranges from 97–100% across the dosing range investigated, which is in line with the literature data on clopidogrel excretion, showing that more than 50% of administered clopidogrel is absorbed [8]. While clopidogrel has been identified as a P-gp substrate in vitro, the literature suggests an absence of significant effects on the rate and extent of intestinal absorption in vivo [9,84]. Hence, P-gp was not included in the model. Moreover, in line with published data, absorbed clopidogrel is almost entirely metabolized, resulting in only minimal levels being detectable in urine and feces [43]. For the two main concurrent metabolic pathways, fractions metabolized of 85–90% and 10–15% can be found in the literature with respect to the amount of clopidogrel absorbed [1,10]. Hence, during parameter optimization, the target corridor for absorbed clopidogrel converted to 2-Oxo-Clo was set at approximately 10–15%, which was closely met by the results of parameter fitting (8.0–14.5% for the dosing range of 75–600 mg clopidogrel).

Comparison of the pharmacokinetic profiles of dose-matched intravenously and perorally administered clopidogrel revealed a 170-fold higher AUC_last_ following intravenous application [7], supporting the hypothesis of an extensive clopidogrel first-pass metabolism. To replicate this effect in the model, the predominantly intestinally expressed CES2 was implemented for the conversion of clopidogrel to Clo-COOH in addition to the primarily hepatically expressed CES1 within the pathway leading to Clo-AG. Moreover, during model development, it became apparent that the intestinal first-pass metabolism must even exceed the hepatic first-pass metabolism for intravenous and peroral administrations to be adequately described. Hence, CES2 expression was limited to the intestine, enabling appropriate modeling of the first-pass effect. Representative of the various UGTs involved, UGT2B7 was integrated for the glucuronidation of Clo-COOH to Clo-AG since it demonstrated the highest impact regarding the glucuronidation in vitro [14,85]. A nonspecific renal clearance was incorporated for the excretion of Clo-AG due to its pronounced hydrophilicity [86].

According to the literature, various CYP enzymes are responsible for the formation of Clo-AM from clopidogrel via 2-Oxo-Clo as part of the second main metabolic pathway, e.g., CYP1A2, CYP2B6, CYP2C9, CYP2C19, and CYP3A4 [13,15,87,88]. The influence of paraoxonase 1 has been discussed with the conclusion that when involved in the metabolism of 2-Oxo-Clo, an inactive thiol is formed rather than Clo-AM [13,87,89,90,91]. CYP2C19 was implemented for both the conversion of clopidogrel to 2-Oxo-Clo and 2-Oxo-Clo to Clo-AM. As highlighted by Kazui et al. [15], CYP2C19 shows the greatest impact on the formation of Clo-AM in vitro (referring to both metabolization steps), with various DGI studies confirming its importance in vivo [51,71,81,82]. Additionally, implementation of another CYP enzyme was required for each of the two metabolization steps to allow subsequent DDI and DGI predictions. Hence, CYP3A4 was included to convert 2-Oxo-Clo to Clo-AM due to its reported high in vitro activity [15]. CYP3A4 was incorporated for the oxidation of clopidogrel to 2-Oxo-Clo as well, given that most in vitro studies have demonstrated its involvement in this step [13,15,24,87,88], and during model development, the observed data were most adequately described upon CYP3A4 integration. Additional transformation of 2-Oxo-Clo into various inactive thiol metabolites was represented by a nonspecific hepatic clearance [92]. Due to the irreversibility of the mechanism of action and the absence of more precise information on the excretion of Clo-AM, an elimination of Clo-AM through covalent binding to platelets was assumed, represented by a nonspecific hepatic clearance in the model since hepatocytes seem to primarily remove platelets from blood [4,93].

The final clopidogrel model was applied for successful prediction of the DGI involving CYP2C19, which is especially noteworthy considering that CYP2C19 is incorporated at two metabolic steps and no phenotype data were available for the intermediate metabolite 2-Oxo-Clo. However, incomplete published data caused limitations of the modeled DGI. For example, due to the exclusive availability of data obtained from Asian individuals, the predictive performance might vary for other ethnicities. Moreover, activity scores were used for DGI modeling, with the classification of genotypes according to phenotypes and assignment of the phenotypes to activity scores adopted from the literature [74]. Consequently, for instance, *2/*2 and *3/*3 genotypes were both classified as PM, and therefore, assigned the same activity score, thus, not allowing differentiation of genotypes by the model. Here, sufficient data on individual genotypes might be required to enable differentiated modeling. Additionally, Clo-AM in particular is subject to a high interindividual variability, e.g., C_max_ values of Clo-AM after peroral administration of 75 mg clopidogrel vary between 9.6 and 27.9 ng/mL in different individuals [44,71]. Here, a rationale for the observed pronounced heterogeneity might be the potentially incorrect assignment of wildtype to studies with no *CYP2C19* genotype information.

Furthermore, a CYP2B6/CYP2C8/CYP2C19/CYP3A4 DDI network was successfully built by coupling the final clopidogrel parent-metabolite model with models of bupropion, montelukast, omeprazole, pioglitazone, repaglinide, and rifampicin [76,77,78,79,80]. The good predictive performance of all DDIs is particularly remarkable considering that only the repaglinide DDI was part of the training dataset informing the intrahepatic concentration of Clo-AG (main CYP2C8 inhibitor), while all other DDIs were fully predicted without any optimized parametrization. Moreover, each interaction parameter was adopted from the literature and both Clo-AM (quantified for perpetrator–clopidogrel–DDI studies) and Clo-AG are secondary metabolites of clopidogrel, thus, requiring more parameters to be included in their modeling. Additionally, only plasma profiles of Clo-AG following the administration of 75 mg clopidogrel were available for model development, whereas some of the CYP2C8 DDI studies involved application of up to 300 mg clopidogrel. Lastly, mechanism-based inactivation of CYP2B6 and CYP2C8 by clopidogrel and Clo-AG was particularly challenging due to the combination of reversible and irreversible inhibition with multiple descriptive parameters required.

Previously published PBPK models of clopidogrel can be found in the literature [31,33,94,95,96,97], each focusing on only one of the two major metabolic pathways, with no model incorporating all four relevant metabolites. The presented clopidogrel model predicts the complex metabolism of clopidogrel including simulations in *CYP2C19* variant allele carriers as well as the comprehensive DDI network involving several perpetrator and victim drugs.

## 5. Conclusions

The developed clopidogrel parent-metabolite whole-body PBPK model shows a good descriptive and predictive performance for all modeled compounds, especially considering model complexity and the partly sparse data availability, e.g., regarding 2-Oxo-Clo. In addition, the model has been successfully applied not only for the prediction and study of the DGI involving CYP2C19 but also of DDIs centered around clopidogrel as CYP2C19 and CYP3A4 substrate as well as CYP2B6, CYP2C8, and CYP2C19 inhibitor. Potential applications of the model include the support of MID3 or the provision of dose recommendations. Further expansion of the model to other DDI partners may be considered in the future once appropriate DDI studies and corresponding perpetrator/victim PBPK models become available. For instance, simulation of DDIs involving potent CYP3A4 inhibitors such as ketoconazole would be beneficial to further examine the role of CYP3A4 in the metabolism of clopidogrel [24]. Although DDGIs involving clopidogrel are of great interest, only data related to pharmacodynamics (PD) are currently available from the literature [98,99,100]. Once PK studies become available, the existing model can be further refined to cover predictions of PK DDGI scenarios as well. Lastly, extending the model in future studies to include clopidogrel PD might further increase its applicability to investigate complex DD(G)I dose–effect relationships.

## Figures and Tables

**Table 1:** Relation between CYP2C19 phenotypes, activity scores, and genotypes according to [74], including assumed relative activity.

Phenotype	Activity Score	Common *CYP2C19* Genotypes	Assumed Activity (%)
Poor metabolizer (PM)	0	*2/*2, *2/*3, *3/*3	0
Intermediate metabolizer (IM)	1	*1/*2, *1/*3	50
Normal metabolizer (NM)	2	*1/*1	100

CYP: cytochrome P450.

## Data Availability

All modeling files, including the clinical study data utilized, can be found at https://github.com/Open-Systems-Pharmacology.

## Supplementary Materials

The following supporting information can be downloaded at https://www.mdpi.com/article/10.3390/pharmaceutics14050915/s1: a comprehensive reference manual containing detailed documentation on development and evaluation of the model. Section S1: PBPK Model Building; Section S2: PBPK Model Evaluation; Section S3: DGI Modeling; Section S4: DDI Network Modeling. References [101,102,103,104,105,106,107,108,109,110,111,112,113,114,115,116,117,118,119,120,121,122,123,124,125,126,127,128,129,130,131,132,133,134,135,136,137,138,139,140,141,142,143,144,145,146,147,148] are cited in the Supplementary Materials.
