# Supplementary material for: Physiologically Based Pharmacokinetic (PBPK) Modeling of Clopidogrel and Its Four Relevant Metabolites for CYP2B6, CYP2C8, CYP2C19, and CYP3A4 Drug–Drug–Gene Interaction Predictions
Source: Pharmaceutics. 2022 Apr 22;14(5):915. doi: 10.3390/pharmaceutics14050915 (PMC9145019; doi:10.3390/pharmaceutics14050915)
Supplement: Supplementary file 1 [file pharmaceutics-14-00915-s001.zip › pharmaceutics-1633046-supplementary.pdf]

Physiologically Based Pharmacokinetic (PBPK) Modeling of Clopidogrel and Its Four Relevant Metabolites for CYP2B6, CYP2C8, CYP2C19, and CYP3A4 Drug–Drug–Gene Interaction Predictions

Supplementary Materials

Helena Leonie Hanae Loer[1], Denise Türk[1], José David Gómez-Mantilla[2], Dominik Selzer[1], Thorsten Lehr[1]

[1]Clinical Pharmacy, Saarland University, Saarbrücken, Germany
[2]Translational Medicine & Clinical Pharmacology, Boehringer Ingelheim Pharma GmbH & Co. KG, 55216 Ingelheim, Germany

Funding:
Thorsten Lehr was supported by the German Federal Ministry of Education and Research (BMBF, Horizon 2020 INSPIRATION grant 643271), under the frame of ERACoSysMed.

Conflict of Interest:
José David Gómez-Mantilla is a paid employee of Boehringer Ingelheim GmbH. Helena Leonie Hanae Loer, Denise Türk, Dominik Selzer, and Thorsten Lehr declare no conflict of interest. The funders had no role in the design of the study; in the collection, analyses, or interpretation of data; in the writing of the manuscript, or in the decision to publish the results.

Corresponding Author:
Thorsten Lehr, PhD
Clinical Pharmacy
Saarland University
Campus C5 3
66123 Saarbrücken
Germany

Contents

S1 PBPK Model Building 4
S1.1 System-Dependent Parameters . . . 4
S1.2 Clinical Study Data . . . 5
S1.3 Drug-Dependent Parameters . . . 8

S2 PBPK Model Evaluation 14
S2.1 Plasma Concentration-Time Profiles (Semilogarithmic) . . . 14
S2.1.1 Clopidogrel . . . 14
S2.1.2 Clopidogrel Carboxylic Acid . . . 17
S2.1.3 Clopidogrel Acyl Glucuronide . . . 20
S2.1.4 2-Oxo-Clopidogrel . . . 20
S2.1.5 Clopidogrel Thiol H4 . . . 21
S2.2 Plasma Concentration-Time Profiles (Linear) . . . 23
S2.2.1 Clopidogrel . . . 23
S2.2.2 Clopidogrel Carboxylic Acid . . . 26
S2.2.3 Clopidogrel Acyl Glucuronide . . . 29
S2.2.4 2-Oxo-Clopidogrel . . . 29
S2.2.5 Clopidogrel Thiol H4 . . . 30
S2.3 Plasma Concentration Goodness-of-Fit Plots . . . 32
S2.4 AUC_{last} and C_{max} Goodness-of-Fit Plots . . . 33
S2.5 MRD of Plasma Concentration Predictions . . . 34
S2.6 Predicted and Observed AUC_{last} and C_{max} Values with Mean GMFEs . . . 36
S2.7 Local Sensitivity Analysis . . . 39
S2.7.1 Method . . . 39
S2.7.2 Results . . . 40

S3 DGI Modeling 46
S3.1 Clinical Study Data . . . 46
S3.2 Plasma Concentration-Time Profiles (Semilogarithmic) . . . 48
S3.3 Plasma Concentration-Time Profiles (Linear) . . . 50
S3.4 DGI AUC_{last} and DGI C_{max} Ratio Goodness-of-Fit Plots . . . 52
S3.5 Predicted and Observed DGI AUC_{last} and DGI C_{max} Ratios with Mean GMFEs . . . 53

S4 DDI Network Modeling 54
S4.1 Types of Interaction Implemented . . . 54
S4.1.1 Competitive Inhibition . . . 54
S4.1.2 Mechanism-Based Inactivation . . . 54
S4.1.3 Induction . . . 54
S4.2 Clinical Study Data . . . 55
S4.3 Implemented Interaction Processes . . . 56
S4.3.1 Bupropion . . . 56
S4.3.2 Montelukast . . . 56
S4.3.3 Omeprazole . . . 56
S4.3.4 Pioglitazone . . . 56
S4.3.5 Repaglinide . . . 56
S4.3.6 Rifampicin . . . 57
S4.4 Drug-Dependent Parameters DDI Partner . . . 58
S4.4.1 Bupropion . . . 58
S4.4.2 Montelukast . . . 61
S4.4.3 Omeprazole . . . 62

S4.4.4 Pioglitazone . . . 63
S4.4.5 Repaglinide . . . 64
S4.4.6 Rifampicin . . . 65
S4.4.7 Partition Coefficients Intracellular : Plasma DDI Partner . . . 67
S4.5 Plasma Concentration-Time Profiles (Semilogarithmic) . . . 68
S4.5.1 Clopidogrel as Victim . . . 68
S4.5.2 Clopidogrel as Perpetrator . . . 69
S4.6 Plasma Concentration-Time Profiles (Linear) . . . 70
S4.6.1 Clopidogrel as Victim . . . 70
S4.6.2 Clopidogrel as Perpetrator . . . 71
S4.7 DDI AUC_{last} and DDI C_{max} Ratio Goodness-of-Fit Plots . . . 72
S4.8 Predicted and Observed DDI AUC_{last} and DDI C_{max} Ratios with Mean GMFEs . . . 73
Bibliography **75**

S1 PBPK Model Building

S1.1 System-Dependent Parameters

Table S1: System-dependent parameters

Enzyme/ Transporter	Mean reference conc.^{a} [µmol/l]	Relative organ expression^b	Localization/ Direction	Half-life liver [h]	Half-life intestine [h]
11β-HSD	1.0^c	Array [1]	intracellular	36	23
AADAC	1.0^c	RT-PCR [2]	intracellular	36	23
CES1	42^d [3]	RT-PCR [2]	intracellular	36	23
CES2	1.02^{d,e} [3]	RT-PCR^f [2]	intracellular	32	23
CYP2B6	1.56 [4]	RT-PCR [5]	intracellular	32	23
CYP2C8	2.56 [4]	RT-PCR [5]	intracellular	23	23
CYP2C9	3.84 [4]	RT-PCR [5]	intracellular	104	23
CYP2C19	0.76 [4]	RT-PCR [5]	intracellular	26	23
CYP3A4	4.32 [4]	RT-PCR [5]	intracellular	36	23
CYP3A5	0.04 [4]	RT-PCR [5]	intracellular	36	23
NRT^g	1.0^c	EST [6]	extracellular membrane	36	23
OATP1B1	0.07^h[7]	RT-PCR [8]	influx	36	23
OATP1B3	1.0^c	Array [1]	influx	36	23
P-gp	1.41 [9]	RT-PCR^i [8]	efflux	36	23
UGT2B7	0.09^j [10]	EST [6]	intracellular	36	23

AADAC: arylacetamide deacetylase, Array: microarray expression profile, CES: carboxylesterase, conc.: concentration, CYP: cytochrome P450, EST: expressed sequence tags, HSD: hydroxysteroid dehydrogenase , NRT: noradrenaline reuptake transporter, OATP: organic-anion-transporting, polypeptide, P-gp: P-glycoprotein, RT-PCR: reverse transcription polymerase chain reaction, UGT: uridine 5'-diphospho-glucuronosyltransferase.
^{a}: µmol protein/l in the tissue of highest expression
^{b}: according to PK-Sim® expression database
^{c}: if no information was available, the mean reference concentration was set to 1.0 µmol/l and the catalytic catalytic rate constant was optimized according to [11]
^{d}: calculated from protein per mg microsomal protein x 40.0 mg microsomal protein per g liver [12],
^{e}: intestinal expression extrapolated from liver expression
^{f}: expression reduced to intestinal mucosa duodenum, upper/ lower jejunum, upper/ lower ileum, colon ascendens/ transversum/ descendens/ sigmoid,
non-mucosal tissue small/ large intestine
^{g}: expression profile used for general binding partner bupropion
^{h}: calculated from transporter per mg membrane protein x 37.0 mg membrane protein per g liver [7]
^{i}: with the relative expression in the intestinal mucosa increased by factor 3.57
^{j}: calculated from transporter per mg membrane protein x 26.2 mg human kidney microsomal protein per g kidney [12]

S1.2 Clinical Study Data

Table S2: Clinical studies of clopidogrel used for PBPK model development

Compound measured	Clopidogrel dosing regimen: Route	Clopidogrel dosing regimen: Dose [mg]	n	Females [%]	Ethnicity implemented	Age [years]	Weight [kg]	Height [cm]	Dataset	Reference
Clo	iv (bolus, s.d.)	0.1	24	46	White American	46.0±13.0	76.3±15.8	169±9	test	Cushing 2012 [13]
Clo	iv (bolus, s.d.)	1	24	50	White American	41.3±14.7	68.4±11.5	165±9	training	Cushing 2012 [13]
Clo	iv (bolus, s.d.)	10	24	46	White American	44.5±14.9	72.2±13.4	166±8	test	Cushing 2012 [13]
Clo	iv (bolus, s.d.)	30	24	46	White American	43.0±15.9	74.9±12.4	170±7	test	Cushing 2012 [13]
Clo	iv (inf, 4 min, s.d.)	100	24	33	White American	44.4±14.1	77.2±12.4	169±9	training	Cushing 2012 [13]
Clo	iv (inf, 8 min, s.d.)	300	24	50	White American	43.3±15.3	75.6±15.8	167±11	training	Cushing 2012 [13]
Clo	po (tab, s.d.)	75	6	50	European	32.2±14.5	-	-	training	Savu 2016 [14]
Clo	po (-, s.d.)	75	10	-	European	-	-	-	training	Silvestro 2013 [15]
Clo	po (-, s.d.)	75	6	-	Asian	-	-	-	test	Nirogi 2006 [16]
Clo	po (tab, s.d.)	75	20	0	Asian	24.3<br>(22–29)	64.1<br>(55–71)	171<br>(163–185)	test	Zou 2012 [17]
Clo	po (tab, s.d.)	75	24	46	White American	33.7±5.2<br>(21–42)	72.4±6.83<br>(59–82)	171±7<br>(160–181)	test	Di Girolamo 2010 [18]
Clo	po (tab, s.d.)	75	92	0	White American	36±8<br>(18–52)	75.6±7.0<br>(60.3–89.7)	174±6<br>(161–191)	test	Brvar 2014 [19]
Clo	po (tab, s.d.)	75	46	0	European	27.3±6.3	58.2±5.0	166.7±5.7	test	McGregor 2016 [20]
Clo	po (tab, s.d.)	150	8	0	Asian	(20–28)	63.9±7.3	-	training	Shin 2007 [21]
Clo	po (tab, s.d.)	150	36	-	European	-	-	-	test	Robinson 2007 [22]
Clo	po (tab, s.d.)	300	27	0	Asian	24.9±2.4	68.0±7.2	174.5±5.0	training	Kim 2016 [23]
Clo	po (tab, s.d.)	300	20	0	Asian	(25–30)	-	-	test	Zhou 2018 [24]
Clo	po (-, l.d./m.d., 5d)	300/75	24	0	European	31.5±5.5<br>(23–40)	80.4±7.4<br>(64–91)	-	training	Härtter 2013 [25]
Clo	po (-, l.d./m.d., 7d)	300/75	8 PM	0	Asian	24.1±2.8	67.3±5.6	-	training	Kim 2008 [26]
Clo	po (-, l.d./m.d., 5d)	300/75	40	0	Asian	26.5±4.0	69.4±6.2	-	test	Kim 2012 [27]
Clo	po (-, l.d./m.d., 6d)	300/75	44	0	Asian	24.3±2.7<br>(19–33)	70.0±8.2<br>(56.7–88.9)	175.5±5.5<br>(163–187)	test	Kim 2009 [28]
Clo	po (-, s.d.)	600	12	0	European	32.8±5.6<br>(22–40)	81.6±9.4<br>(68–100)	-	training	Härtter 2013 [25]
Clo	po (tab, s.d.)	600	14	43	European	22.0±1.5<br>(21–26)	69±10<br>(54–88)	174±6<br>(164–183)	training	Holmberg 2014 [29]

-: not given, 2-Oxo-Clo: 2-Oxo-clopidogrel, Clo: clopidogrel, Clo-AG: clopidogrel acyl glucuronide, Clo-AM: clopidogrel thiol H4, Clo-COOH: clopidogrel carboxylic acid, d: dosage period in days, inf: infusion, iv: intravenous, l.d.: loading dose, m.d.: maintenance dose (once daily), n: number of participants, NM: cytochrome P450 normal metabolizer, PM: cytochrome P450 2C19 poor metabolizer, po: peroral, s.d.: single dose, tab: tablet; values for age, weight and height are shown as mean ± standard deviation (range).

Table S2: Clinical studies of clopidogrel used for PBPK model development *(continued)*

Compound measured	Clopidogrel dosing regimen: Route	Clopidogrel dosing regimen: Dose [mg]	n	Females [%]	Ethnicity implemented	Age [years]	Weight [kg]	Height [cm]	Dataset	Reference
Clo-COOH	iv (bolus, s.d.)	0.1	24	46	White American	46.0±13.0	76.3±15.8	169±9	test	Cushing 2012 [13]
Clo-COOH	iv (bolus, s.d.)	1	24	50	White American	41.3±14.7	68.4±11.5	165±9	training	Cushing 2012 [13]
Clo-COOH	iv (bolus, s.d.)	10	24	46	White American	44.5±14.9	72.2±13.4	166±8	training	Cushing 2012 [13]
Clo-COOH	iv (bolus, s.d.)	30	24	46	White American	43.0±15.9	74.9±12.4	170±7	test	Cushing 2012 [13]
Clo-COOH	iv (inf, 4 min, s.d.)	100	24	33	White American	44.4±14.1	77.2±12.4	169±9	training	Cushing 2012 [13]
Clo-COOH	iv (inf, 8 min, s.d.)	300	24	50	White American	43.3±15.3	75.6±15.8	167±11	training	Cushing 2012 [13]
Clo-COOH	po (tab, s.d.)	75	6	50	European	32.2±14.5	-	-	training	Savu 2016 [14]
Clo-COOH	po (-, s.d.)	75	10	-	European	-	-	-	training	Silvestro 2013 [15]
Clo-COOH	po (tab, s.d.)	75	20	0	Asian	24.3 (22–29)	64.1 (55–71)	171 (163–185)	test	Zou 2012 [17]
Clo-COOH	po (-, s.d.)	75	10	-	European	-	-	-	test	Silvestro 2011 [30]
Clo-COOH	po (tab, s.d.)	75	42	50	White American	28.7±7.6 (18–43)	66.7±8.1 (48.8–81.2)	170±1.1 (150–190)	test	Junior 2010 [31]
Clo-COOH	po (tab, s.d.)	75	76	0	Asian	29.1±6.9 (18–45)	77.7±8.8	-	test	Yousef 2013 [32]
Clo-COOH	po (tab, s.d.)	75	12	0	Asian	27.9±3.1	76.5±4.0	174.6±5.4	test	Souri 2006 [33]
Clo-COOH	po (tab, s.d.)	75	32	-	European	-	-	-	test	Ksycinska 2006 [34]
Clo-COOH	po (tab, s.d.)	75	92	0	White American	36±8 (18–52)	75.6±7.0 (60.3–89.7)	174±6 (161–191)	test	Brvar 2014 [19]
Clo-COOH	po (tab, s.d.)	150	24	0	Asian	24.8±6.5	75.3±4.2	-	training	Bahrami 2008 [35]
Clo-COOH	po (tab, s.d.)	300	24	23	White American	42±13 (19–59)	82.8±14.3 (57.5–107)	176±8 (158–190)	training	Small 2008 [36]
Clo-COOH	po (-, l.d./m.d., 5d)	300/75	24	0	European	31.5±5.5	80.4±7.4	-	training	Härtter 2013 [25]
Clo-COOH	po (-, l.d./m.d., 5d)	300/75	40	0	Asian	26.5±4.0	69.4±6.2	-	test	Kim 2012 [27]
Clo-COOH	po (-, l.d./m.d., 6d)	300/75	44	0	Asian	24.3±2.7 (19–33)	70.0±8.2 (56.7–88.9)	175.5±5.5 (163–187)	test	Kim 2009 [28]
Clo-COOH	po (-, s.d.)	600	12	0	European	32.8±5.6	81.6±9.4	-	training	Härtter 2013 [25]
Clo-AG	po (tab, s.d.)	75	6	50	European	32.2±14.5	-	-	training	Savu 2016 [14]
Clo-AG	po (-, s.d.)	75	10	-	European	-	-	-	training	Silvestro 2013 [15]
Clo-AG	po (-, s.d.)	75	10	-	European	-	-	-	test	Silvestro 2011 [30]

-: not given, 2-Oxo-Clo: 2-Oxo-clopidogrel, Clo: clopidogrel, Clo-AG: clopidogrel acyl glucuronide, Clo-AM: clopidogrel thiol H4, Clo-COOH: clopidogrel carboxylic acid, d: dosage period in days, inf: infusion, iv: intravenous, l.d.: loading dose, m.d.: maintenance dose (once daily), n: number of participants, NM: cytochrome P450 normal metabolizer, PM: cytochrome P450 2C19 poor metabolizer, po: peroral, s.d.: single dose, tab: tablet; values for age, weight and height are shown as mean ± standard deviation (range).

Table S2: Clinical studies of clopidogrel used for PBPK model development *(continued)*

Compound measured	Clopidogrel dosing regimen Route	Dose [mg]	n	Females [%]	Ethnicity implemented	Age [years]	Weight [kg]	Height [cm]	Dataset	Reference
2-Oxo-Clo	po (-, s.d.)	75	5	-	European	-	-	-	training	Silvestro 2013 [15]
Clo-AM	po (-, s.d.)	75	5	-	European	-	-	-	test	Silvestro 2013 [15]
Clo-AM	po (-, m.d., 10d)	75	9	33	Asian	30.3±7.5	63.8±8.5	165.4±7.2	test	Li 2018 [37]
Clo-AM	po (tab, s.d.)	300	27	0	Asian	24.9±2.4	68.0±7.2	174.5±5.0	training	Kim 2016 [23]
Clo-AM	po (tab, s.d.)	300	20	0	Asian	(25–30)	-	-	test	Zhou 2018 [24]
Clo-AM	po (tab, s.d.)	300	36	39	Japanese	-	-	-	test	Umemura 2016 [38]
Clo-AM	po (tab, s.d.)	300	66	29	White American	42±17	76.3±11.6	-	test	Takahashi 2008 [39]
Clo-AM	po (tab, s.d.)	300	16	19	Asian	31±10	65.2±8.9	-	test	Small 2010 [40]
Clo-AM	po (-, l.d./m.d., 7d)	300/75	9 NM	0	Japanese	(20–35)	(50.4–87.4)	-	training	Kobayashi 2015 [41]
Clo-AM	po (-, l.d./m.d., 7d)	300/75	9 PM	0	Japanese	(20–35)	(50.4–87.4)	-	training	Kobayashi 2015 [41]
Clo-AM	po (-, l.d./m.d., 5d)	300/75	65	17	White American	33.8±10<br>(29–44)	75.9±10.8<br>(49.6–76.8)	171.7±9.3	training	Angiolillo 2011 [42]
Clo-AM	po (-, l.d./m.d., 5d)	300/75	66	0	European	31±10	78.5±8.9	175.3±6.3	test	Hurbin 2012 [43]
Clo-AM	po (-, l.d./m.d., 5d)	300/75	65	-	White American	-	-	-	test	Furlong 2013 [44]
Clo-AM	po (tab, s.d.)	600	14	43	European	22.0±1.5<br>(21–26)	69±10<br>(54–88)	174±6<br>(164–183)	training	Holmberg 2014 [29]

-: not given, 2-Oxo-Clo: 2-Oxo-clopidogrel, Clo: clopidogrel, Clo-AG: clopidogrel acyl glucuronide, Clo-AM: clopidogrel thiol H4, Clo-COOH: clopidogrel carboxylic acid, d: dosage period in days, inf: infusion, iv: intravenous, l.d.: loading dose, m.d.: maintenance dose (once daily), n: number of participants, NM: cytochrome P450 normal metabolizer, PM: cytochrome P450 2C19 poor metabolizer, po: peroral, s.d.: single dose, tab: tablet; values for age, weight and height are shown as mean ± standard deviation (range).

S1.3 Drug-Dependent Parameters

Table S3: Drug-dependent parameters of the final clopidogrel parent-metabolite PBPK model

Parameter	Unit	Value	Source	Literature	Reference	Description
			**Clopidogrel**			
Molecular weight	g/mol	321.82	Lit.	321.82	[46]	Molecular weight
TPSA*	Å^2		Lit.	29.54	[46]	Topological polar surface area
pKa, base		4.66^a	Lit.	4.6–4.77	[46–48]	Acid dissociation constant
Solubility (pH)	mg/ml	0.06 (7.4)	Lit.	0.06 (7.4)	[46]	Solubility
Lipophilicity	log units	3.23	Opt.	logP: 2.50–4.03; logD$_{7.4}$: 4.03^b	[46–48]	Lipophilicity
f_u	%	2	Lit.	2	[49]	Fraction unbound
CES1 K_M → Clo-COOH	µmol/l	46.40	Lit.	46.40^c	[50]	Michaelis-Menten constant
CES1 k_{cat} → Clo-COOH	1/min	2.51	Opt.	3558.00	[50]	Catalytic rate constant
CES2 K_M → Clo-COOH	µmol/l	14.08	Lit.	14.08^c	[51]	Michaelis-Menten constant
CES2 k_{cat} → Clo-COOH	1/min	4960.15	Opt.	357.00	[51]	Catalytic rate constant
CYP2C19 K_M → 2-Oxo-Clo	µmol/l	0.05	Lit.	0.05^c	[52]	Michaelis-Menten constant
CYP2C19 NM k_{cat} → 2-Oxo-Clo	1/min	28.51	Opt.	7.52	[52]	Catalytic rate constant
CYP2C19 IM k_{cat} → 2-Oxo-Clo	1/min	14.26	Calc.	-	-	Catalytic rate constant
CYP2C19 PM k_{cat} → 2-Oxo-Clo	1/min	0	Calc.	-	-	Catalytic rate constant
CYP3A4 K_M → 2-Oxo-Clo	µmol/l	2.07	Lit.	2.07^c	[51]	Michaelis-Menten constant
CYP3A4 k_{cat} → 2-Oxo-Clo	1/min	55.36	Opt.	266.00	[51]	Catalytic rate constant
GFR fraction		1	Asm.	-	-	Filtered drug in the urine
EHC continuous fraction		1	Asm.	-	-	Bile fraction continously released
Intestinal permeability	cm/min	$1.50 \cdot 10^{-4}$	Opt.	$7.98 \cdot 10^{-6}$	[53]	Transcellular intestinal permeability
Cellular permeability	cm/min	0.07	Calc.	Charge dependent Schmitt	[54]	Permeability into the cellular space
Partition coefficients			Calc.	PK-Sim Standard	[55]	Organ-plasma partition coefficients
Dissolution time (Weibull)	min	15.16	Lit.	15.16^d	[56]	Dissolution time (50%)
Dissolution shape (Weibull)		1.16	Opt.	3.17	[56]	Dissolution shape

-: not available, *: no model parameter, listed for additional information, ^{a}: mean, ^{b}: calculated by Chemicalize, ^{c}: *in vitro* values corrected for binding in the assay ($f_{u,mic}$) calculated according to [45], ^{d}: obtained from literature dissolution profile, 2-Oxo-Clo: 2-Oxo-clopidogrel, asm.: assumed, calc.: calculated, CES: carboxylesterase, CI: competitive inhibition, Clo-AG: clopidogrel acyl glucuronide, Clo-AM: clopidogrel thiol H4, Clo-COOH: clopidogrel carboxylic acid, conc.: concentration, CYP: cytochrome P450, EHC: enterohepatic circulation, GFR: glomerular filtration rate, IM: intermediate metabolizer, lit.: literature, MBI: mechanism-based inactivation, NM: normal metabolizer, OATP: organic-anion-transporting polypeptide, opt.: optimized, PM: poor metabolizer, UGT: uridine 5'-diphospho-glucuronosyltransferase.

Table S3: Drug-dependent parameters of the final clopidogrel parent-metabolite PBPK model *(continued)*

Parameter	Unit	Value	Source	Literature	Reference	Description
CYP1A2 K_i	µmol/l	12.15	Lit.	12.15	[57]	Conc. for half-maximal inhibition (CI)
CYP2B6 K_i	µmol/l	$7.3 \cdot 10^{-3}$	Lit.	$7.3 \cdot 10^{-3}$–$1.7 \cdot 10^{-2}$[c]	[57–59]	Conc. for half-maximal inhibition (CI)
CYP2B6 K_I	µmol/l	$5.7 \cdot 10^{-3}$	Lit.	$5.7 \cdot 10^{-3}$–2.4[c]	[59–63]	Conc. for half-maximal inactivation (MBI)
CYP2B6 k_{inact}	1/min	1.3	Lit.	$3.7 \cdot 10^{-2}$–1.9	[59–63]	Maximum inactivation rate constant (MBI)
CYP2C8 K_i	µmol/l	8.10[a]	Lit.	1.4–16.6[c]	[48, 57, 64, 65]	Conc. for half-maximal inhibition (CI)
CYP2C9 K_i	µmol/l	6.70	Lit.	6.70	[57]	Conc. for half-maximal inhibition (CI)
CYP2C19 K_i	µmol/l	5.00[a]	Lit.	0.262, 9.74[c]	[57, 66]	Conc. for half-maximal inhibition (CI)
CYP2C19 K_I	µmol/l	4.86	Lit.	4.86[c]	[66]	Conc. for half-maximal inactivation (MBI)
CYP2C19 k_{inact}	1/min	$5.57 \cdot 10^{-2}$	Lit.	$5.57 \cdot 10^{-2}$	[66]	Maximum inactivation rate constant (MBI)
CYP3A4 K_i	µmol/l	17.41[a]	Lit.	3.7, 31.13[c]	[48, 64]	Conc. for half-maximal inhibition (CI)
CYP3A4 K_I	µmol/l	19.36	Lit.	19.36[c]	[48]	Conc. for half-maximal inactivation (MBI)
CYP3A4 k_{inact}	1/min	$5.3 \cdot 10^{-2}$	Lit.	$5.3 \cdot 10^{-2}$	[48]	Maximum inactivation rate constant (MBI)
OATP1B1 K_i	µmol/l	1.975	Lit.	1.975	[67]	Conc. for half-maximal inhibition (CI)
			**Clopidogrel carboxylic acid**			
Molecular weight	g/mol	307.79	Lit.	307.79	[68]	Molecular weight
TPSA*	Å^2		Lit.	40.54	[68]	Topological polar surface area
pKa, base		5.75	Lit.	5.75	[68]	Acid dissociation constant
pKa, acid		1.79	Lit.	1.79	[68]	Acid dissociation constant
Solubility (pH)	mg/ml	3.98 (7.4)	Lit.	3.98 (7.4)	[68]	Solubility
Lipophilicity	log units	3.01	Opt.	logP: 2.57; log$D_{7.4}$: 1.03[b]	[68]	Lipophilicity
f_u	%	0.10	Opt.	≤ 1	[69]	Fraction unbound
UGT2B7 K_M → Clo-AG	µmol/l	20.30	Lit.	20.30[c]	[70]	Michaelis-Menten constant
UGT2B7 k_{cat} → Clo-AG	1/min	14637.76	Opt.	50.50	[70]	Catalytic rate constant
GFR fraction		1	Asm.	-	-	Filtered drug in the urine
EHC continuous fraction		1	Asm.	-	-	Bile fraction continously released
Intestinal permeability	cm/min	$1.45 \cdot 10^{-4}$	Calc.	$1.45 \cdot 10^{-4}$	-	Transcellular intestinal permeability
Cellular permeability	cm/min	0.05	Calc.	PK-Sim Standard	[55]	Permeability into the cellular space
Partition coefficients			Calc.	PK-Sim Standard	[55]	Organ-plasma partition coefficients
CYP2C8 K_i	µmol/l	61.00[a]	Lit.	54, 68	[64]	Conc. for half-maximal inhibition (CI)
CYP3A4 K_i	µmol/l	280.00	Lit.	280	[64]	Conc. for half-maximal inhibition (CI)

-: not available, *: no model parameter, listed for additional information, [a]: mean, [b]: calculated by Chemicalize, [c]: *in vitro* values corrected for binding in the assay ($f_{u,mic}$) calculated according to [45], [d]: obtained from literature dissolution profile, 2-Oxo-Clo: 2-Oxo-clopidogrel, asm.: assumed, calc.: calculated, CES: carboxylesterase, CI: competitive inhibition, Clo-AG: clopidogrel acyl glucuronide, Clo-AM: clopidogrel thiol H4, Clo-COOH: clopidogrel carboxylic acid, conc.: concentration, CYP: cytochrome P450, EHC: enterohepatic circulation, GFR: glomerular filtration rate, IM: intermediate metabolizer, lit.: literature, MBI: mechanism-based inactivation, NM: normal metabolizer, OATP: organic-anion-transporting polypeptide, opt.: optimized, PM: poor metabolizer, UGT: uridine 5'-diphospho-glucuronosyltransferase.

Table S3: Drug-dependent parameters of the final clopidogrel parent-metabolite PBPK model *(continued)*

Parameter	Unit	Value	Source	Literature	Reference	Description
**Clopidogrel acyl glucuronide**						
Molecular weight	g/mol	483.92	Lit.	483.92	[71]	Molecular weight
TPSA*	Å^2		Lit.	136.76	[71]	Topological polar surface area
pKa, base		4.42^a	Lit.	4.23, 4.60	[48, 71]	Acid dissociation constant
pKa, acid		2.83^a	Lit.	2.65, 3.01	[48, 71]	Acid dissociation constant
Solubility (pH)	mg/ml	74.17 (7.4)	Lit.	74.17 (7.4)	[71]	Solubility
Lipophilicity	log units	0.02	Opt.	logP: 0.264, 1.345; logD$_{7.4}$: -1.53^b	[48, 71]	Lipophilicity
f_u	%	1	Lit.	1	[48]	Fraction unbound
CL_{ren} → sink	1/min	1076.46	Opt.	-	-	Unspecific renal clearance
EHC continuous fraction		1	Asm.	-	-	Bile fraction continously released
Intestinal permeability	cm/min	$1.71 \cdot 10^{-8}$	Calc.	$1.71 \cdot 10^{-8}$	-	Transcellular intestinal permeability
Cellular permeability	cm/min	$3.10 \cdot 10^{-6}$	Calc.	PK-Sim Standard	[55]	Permeability into the cellular space
Partition coefficients			Calc.	PK-Sim Standard	[55]	Organ-plasma partition coefficients
CYP2C8 K_i	µmol/l	10.80	Lit.	1.9–28.19^c	[48, 64]	Conc. for half-maximal inhibition (CI)
CYP2C8 K_I	µmol/l	9.87	Lit.	9.87^c	[48]	Conc. for half-maximal inactivation (MBI)
CYP2C8 k_{inact}	1/min	$4.7 \cdot 10^{-2}$	Lit.	$4.7 \cdot 10^{-2}$	[48]	Maximum inactivation rate constant (MBI)
CYP3A4 K_i	µmol/l	95.46^a	Lit.	21, 169.92^c	[48, 64]	Conc. for half-maximal inhibition (CI)
OATP1B1 K_i	µmol/l	5.45	Lit.	5.45	[67]	Conc. for half-maximal inhibition (CI)

-: not available, *: no model parameter, listed for additional information, ^{a}: mean, ^{b}: calculated by Chemicalize, ^{c}: *in vitro* values corrected for binding in the assay ($f_{u,mic}$) calculated according to [45], ^{d}: obtained from literature dissolution profile, 2-Oxo-Clo: 2-Oxo-clopidogrel, asm.: assumed, calc.: calculated, CES: carboxylesterase, CI: competitive inhibition, Clo-AG: clopidogrel acyl glucuronide, Clo-AM: clopidogrel thiol H4, Clo-COOH: clopidogrel carboxylic acid, conc.: concentration, CYP: cytochrome P450, EHC: enterohepatic circulation, GFR: glomerular filtration rate, IM: intermediate metabolizer, lit.: literature, MBI: mechanism-based inactivation, NM: normal metabolizer, OATP: organic-anion-transporting polypeptide, opt.: optimized, PM: poor metabolizer, UGT: uridine 5'-diphospho-glucuronosyltransferase.

Table S3: Drug-dependent parameters of the final clopidogrel parent-metabolite PBPK model *(continued)*

Parameter	Unit	Value	Source	Literature	Reference	Description
**2-Oxo-clopidogrel**						
Molecular weight	g/mol	337.82	Lit.	337.82	[72]	Molecular weight
TPSA*	Å^2		Lit.	46.61	[72]	Topological polar surface area
pKa, base		4.18^a	Lit.	3.945, 4.42	[72, 73]	Acid dissociation constant
pKa, acid		8.46	Lit.	8.46	[72]	Acid dissociation constant
Solubility (pH)	mg/ml	0.02 (7.4)	Lit.	0.02 (7.4)	[72]	Solubility
Lipophilicity	log units	3.06	Opt.	logP: 2.868; logD$_{7.4}$: 2.83^b	[72]	Lipophilicity
f_u	%	7.42	Lit.	7.42	[74]	Fraction unbound
CYP2C19 K_M → Clo-AM	µmol/l	3.03	Lit.	3.03^c	[52]	Michaelis-Menten constant
CYP2C19 NM k_{cat} → Clo-AM	1/min	0.15	Opt.	9.06	[52]	Catalytic rate constant
CYP2C19 IM k_{cat} → Clo-AM	1/min	0.07	Calc.	-	-	Catalytic rate constant
CYP2C19 PM k_{cat} → Clo-AM	1/min	0	Calc.	-	-	Catalytic rate constant
CYP3A4 K_M → Clo-AM	µmol/l	6.97	Lit.	6.97^c	[52]	Michaelis-Menten constant
CYP3A4 k_{cat} → Clo-AM	1/min	33.64	Opt.	3.63	[52]	Catalytic rate constant
CL_{hep} → sink	1/min	$3.33 \cdot 10^{-3}$	Opt.	-	-	Unspecific hepatic clearance
GFR fraction		1	Asm.	-	-	Filtered drug in the urine
EHC continuous fraction		1	Asm.	-	-	Bile fraction continously released
Intestinal permeability	cm/min	$1.03 \cdot 10^{-4}$	Calc.	$1.03 \cdot 10^{-4}$	-	Transcellular intestinal permeability
Cellular permeability	cm/min	0.03	Calc.	PK-Sim Standard	[55]	Permeability into the cellular space
Partition coefficients			Calc.	Schmitt	[75]	Organ-plasma partition coefficients
CYP2B6 K_i	µmol/l	0.65	Lit.	0.65	[57]	Conc. for half-maximal inhibition (CI)
CYP2B6 K_I	µmol/l	0.71	Lit.	0.71^c	[60]	Conc. for half-maximal inactivation (MBI)
CYP2B6 k_{inact}	1/min	$1.63 \cdot 10^{-1}$	Lit.	$1.63 \cdot 10^{-1}$	[60]	Maximum inactivation rate constant (MBI)
CYP2C8 K_i	µmol/l	8.77^a	Lit.	2.1–16.95^c	[48, 57, 64]	Conc. for half-maximal inhibition (CI)
CYP2C19 K_i	µmol/l	7.49^a	Lit.	0.50–14.49^c	[57, 66]	Conc. for half-maximal inhibition (CI)
CYP3A4 K_i	µmol/l	4.05^a	Lit.	2.4, 5.70^c	[48, 64]	Conc. for half-maximal inhibition (CI)
OATP1B1 K_i	µmol/l	4.09	Lit.	4.09	[67]	Conc. for half-maximal inhibition (CI)

-: not available, *: no model parameter, listed for additional information, ^{a}: mean, ^{b}: calculated by Chemicalize, ^{c}: *in vitro* values corrected for binding in the assay ($f_{u,mic}$) calculated according to [45], ^{d}: obtained from literature dissolution profile, 2-Oxo-Clo: 2-Oxo-clopidogrel, asm.: assumed, calc.: calculated, CES: carboxylesterase, CI: competitive inhibition, Clo-AG: clopidogrel acyl glucuronide, Clo-AM: clopidogrel thiol H4, Clo-COOH: clopidogrel carboxylic acid, conc.: concentration, CYP: cytochrome P450, EHC: enterohepatic circulation, GFR: glomerular filtration rate, IM: intermediate metabolizer, lit.: literature, MBI: mechanism-based inactivation, NM: normal metabolizer, OATP: organic-anion-transporting polypeptide, opt.: optimized, PM: poor metabolizer, UGT: uridine 5'-diphospho-glucuronosyltransferase.

Table S3: Drug-dependent parameters of the final clopidogrel parent-metabolite PBPK model *(continued)*

Parameter	Unit	Value	Source	Literature	Reference	Description
**Clopidogrel thiol H4**						
Molecular weight	g/mol	355.83	Lit.	355.83	[76]	Molecular weight
TPSA*	Å^2		Lit.	66.84	[76]	Topological polar surface area
pKa, base		4.93^a	Lit.	4.43–5.45	[47, 73, 76]	Acid dissociation constant
pKa1, acid		3.15^a	Lit.	2.47–3.98	[47, 73, 76]	Acid dissociation constant
pKa2, acid		9.49^a	Lit.	9.15, 9.82	[47, 76]	Acid dissociation constant
Solubility (pH)	mg/ml	9.83 (7.4)	Lit.	9.83 (7.4)	[76]	Solubility
Lipophilicity	log units	3.00	Opt.	logP: 1.137–1.96; logD$_{7.4}$: -0.48^b	[47, 73, 76]	Lipophilicity
f_u	%	0.97	Opt.	≤ 1	[69]	Fraction unbound
$CL_{hep} \rightarrow$ sink	1/min	452.55	Opt.	-	-	Unspecific hepatic clearance
GFR fraction		1	Asm.	-	-	Filtered drug in the urine
EHC continuous fraction		1	Asm.	-	-	Bile fraction continously released
Intestinal permeability	cm/min	$7.03 \cdot 10^{-5}$	Calc.	$7.03 \cdot 10^{-5}$	-	Transcellular intestinal permeability
Cellular permeability	cm/min	0.02	Calc.	PK-Sim Standard	[55]	Permeability into the cellular space
Partition coefficients			Calc.	Rodgers + Rowland	[77, 78]	Organ-plasma partition coefficients

-: not available, *: no model parameter, listed for additional information, ^{a}: mean, ^{b}: calculated by Chemicalize, ^{c}: *in vitro* values corrected for binding in the assay ($f_{u,mic}$) calculated according to [45], ^{d}: obtained from literature dissolution profile, 2-Oxo-Clo: 2-Oxo-clopidogrel, asm.: assumed, calc.: calculated, CES: carboxylesterase, CI: competitive inhibition, Clo-AG: clopidogrel acyl glucuronide, Clo-AM: clopidogrel thiol H4, Clo-COOH: clopidogrel carboxylic acid, conc.: concentration, CYP: cytochrome P450, EHC: enterohepatic circulation, GFR: glomerular filtration rate, IM: intermediate metabolizer, lit.: literature, MBI: mechanism-based inactivation, NM: normal metabolizer, OATP: organic-anion-transporting polypeptide, opt.: optimized, PM: poor metabolizer, UGT: uridine 5'-diphospho-glucuronosyltransferase.

Table S4: Partition coefficients between intracellular space and plasma of the final clopidogrel parent-metabolite PBPK model

Tissue	Clopidogrel[a]	Clo-COOH[a]	Clo-AG[a]	2-Oxo-Clo[b]	Clo-AM[c]
Bone	9.31	0.28	$7.95 \cdot 10^{-3}$	2.06	0.10
Brain	3.81	0.12	$9.38 \cdot 10^{-3}$	9.36	0.05
Fat	27.21	0.82	$9.97 \cdot 10^{-3}$	75.02	0.05
Gonads	1.16	0.04	$8.55 \cdot 10^{-3}$	2.87	0.05
Heart	3.54	0.11	$8.67 \cdot 10^{-3}$	9.53	0.16
Kidney	1.91	0.06	$8.60 \cdot 10^{-3}$	5.44	0.13
Stomach	2.22	0.07	$8.82 \cdot 10^{-3}$	5.93	0.16
Small intestine	2.22	0.07	$8.82 \cdot 10^{-3}$	5.93	0.16
Large intestine	2.22	0.07	$8.82 \cdot 10^{-3}$	5.93	0.16
Liver periportal	2.50	0.08	$8.53 \cdot 10^{-3}$	7.03	0.09
Liver pericentral	2.50	0.08	$8.53 \cdot 10^{-3}$	7.03	0.09
Lung	0.49	0.02	$8.51 \cdot 10^{-3}$	3.65	0.21
Muscle	0.59	0.02	$8.57 \cdot 10^{-3}$	1.25	0.07
Pancreas	2.83	0.09	$8.18 \cdot 10^{-3}$	4.34	0.06
Skin	3.63	0.11	$7.70 \cdot 10^{-3}$	12.31	0.28
Spleen	0.71	0.02	$8.31 \cdot 10^{-3}$	2.15	0.10
Saliva	0.02	$1.00 \cdot 10^{-3}$	0.01	0.07	$9.66 \cdot 10^{-3}$

[a]: estimated via PK-Sim Standard [55], [b]: estimated via Schmitt [75], [c]: estimated via Rodgers + Rowland [77, 78], 2-Oxo-Clo: 2-Oxo-clopidogrel, Clo-AG: clopidogrel acyl glucuronide, Clo-AM: clopidogrel thiol H4, Clo-COOH: clopidogrel carboxylic acid.

S2 PBPK Model Evaluation

S2.1 Plasma Concentration-Time Profiles (Semilogarithmic)

S2.1.1 Clopidogrel

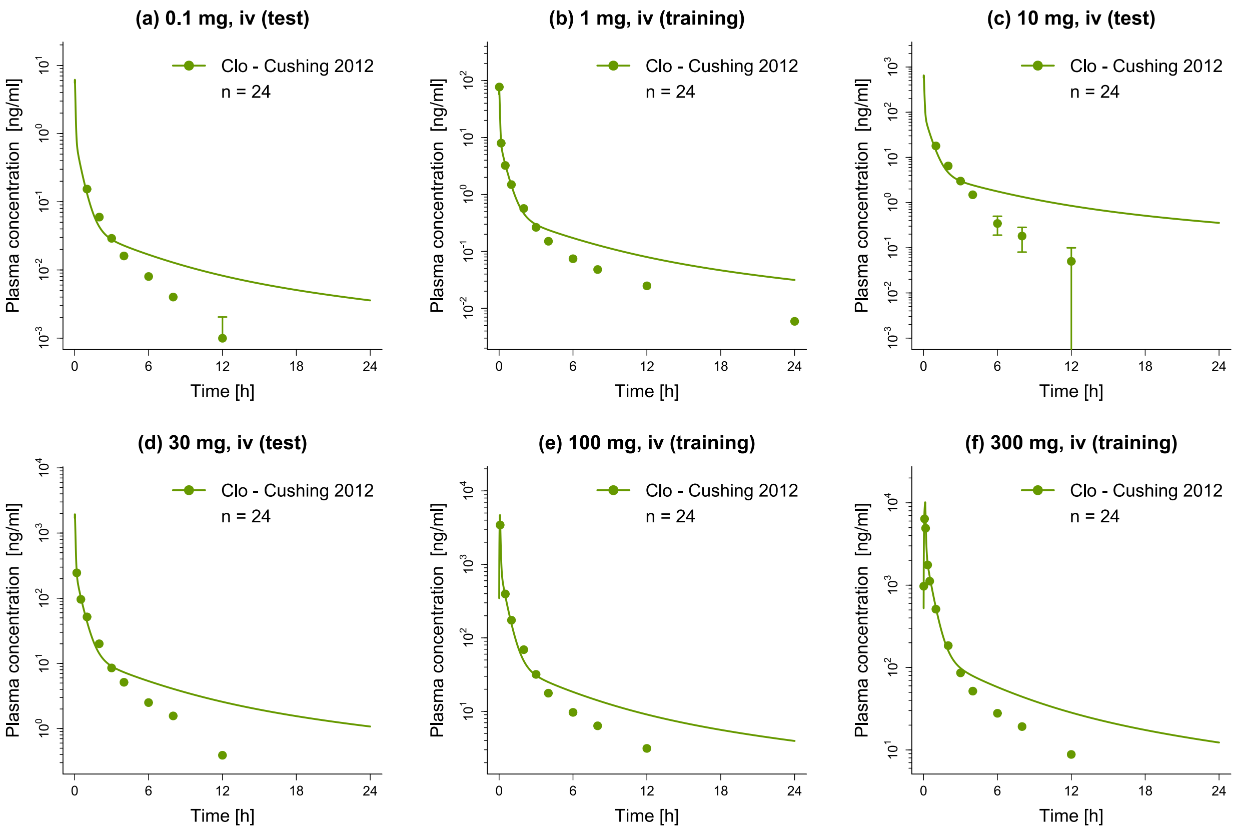

Figure S1: Semilogarithmic plots of predicted plasma concentration-time profiles of clopidogrel. Solid lines represent the model predictions, while corresponding observed data are shown as symbols (± standard deviation, if available) [13]. Clo: clopidogrel, iv: intravenous, n: number of participants.

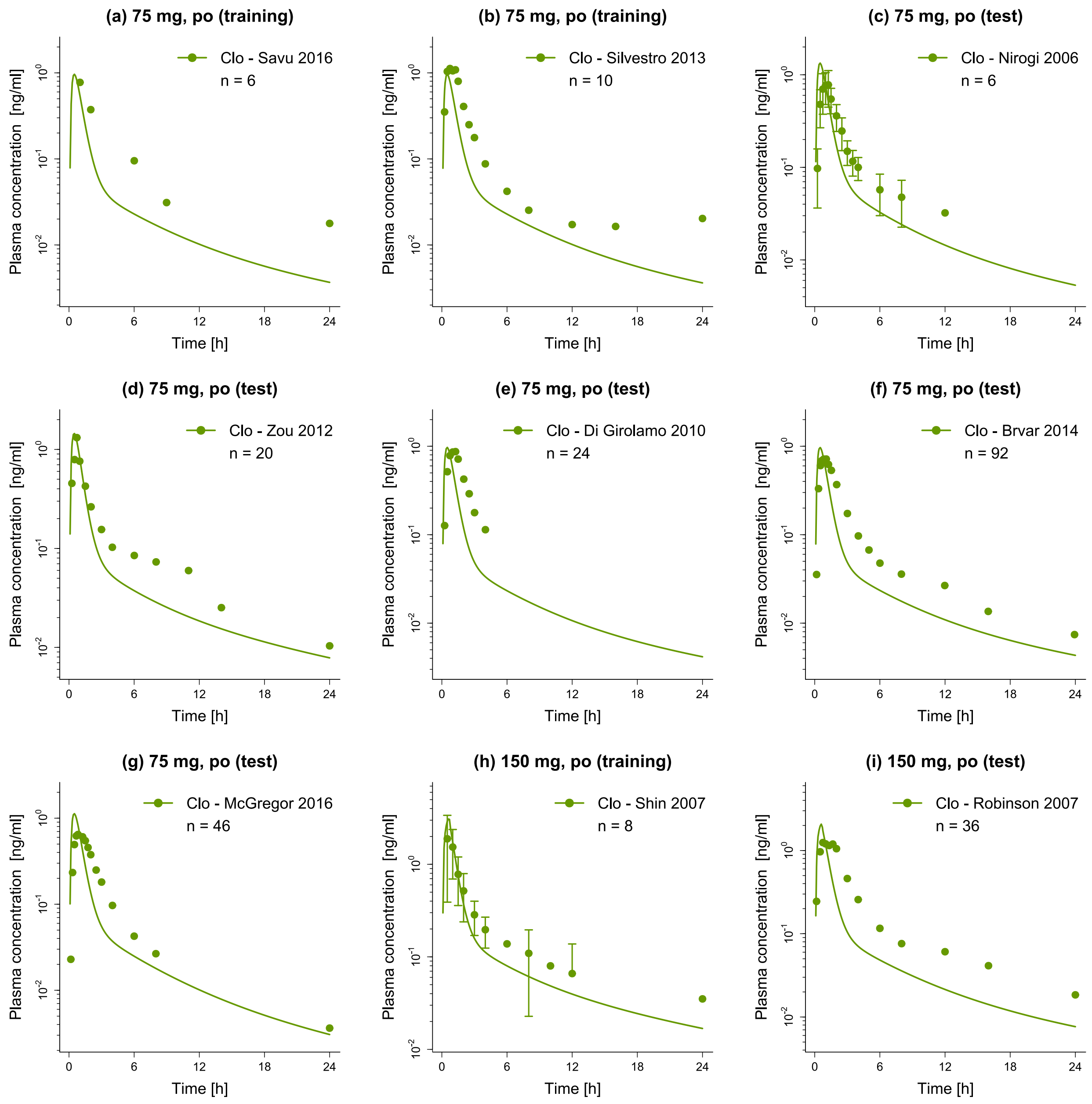

Figure S2: Semilogarithmic plots of predicted plasma concentration-time profiles of clopidogrel. Solid lines represent the model predictions, while corresponding observed data are shown as symbols ($\pm$ standard deviation, if available) [14–22]. Clo: clopidogrel, n: number of participants, po: peroral.

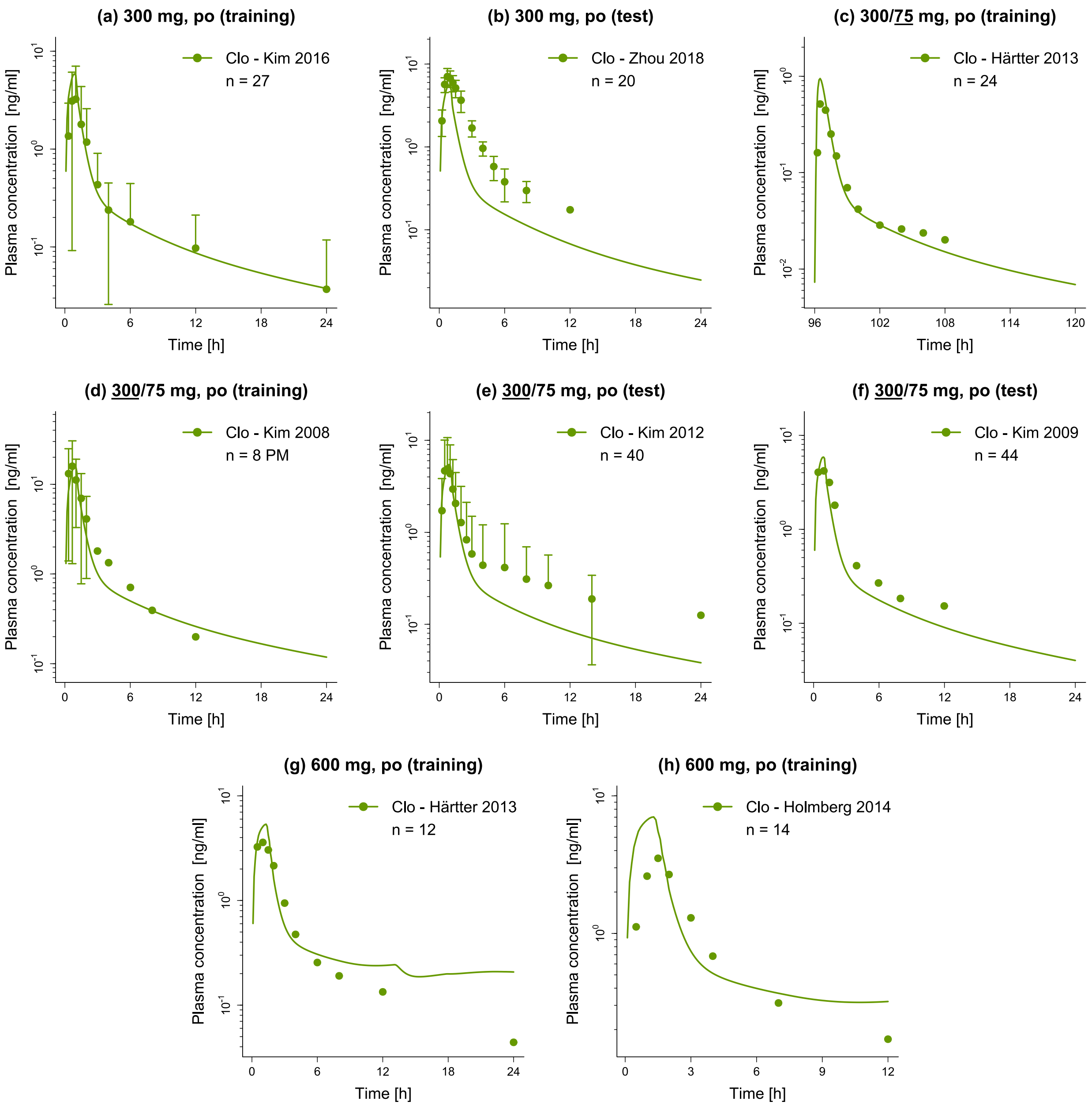

Figure S3: Semilogarithmic plots of predicted plasma concentration-time profiles of clopidogrel. Solid lines represent the model predictions, while corresponding observed data are shown as symbols (± standard deviation, if available) [23–29]. Clo: clopidogrel, n: number of participants, PM: cytochrome P450 2C19 poor metabolizer, po: peroral.

S2.1.2 Clopidogrel Carboxylic Acid

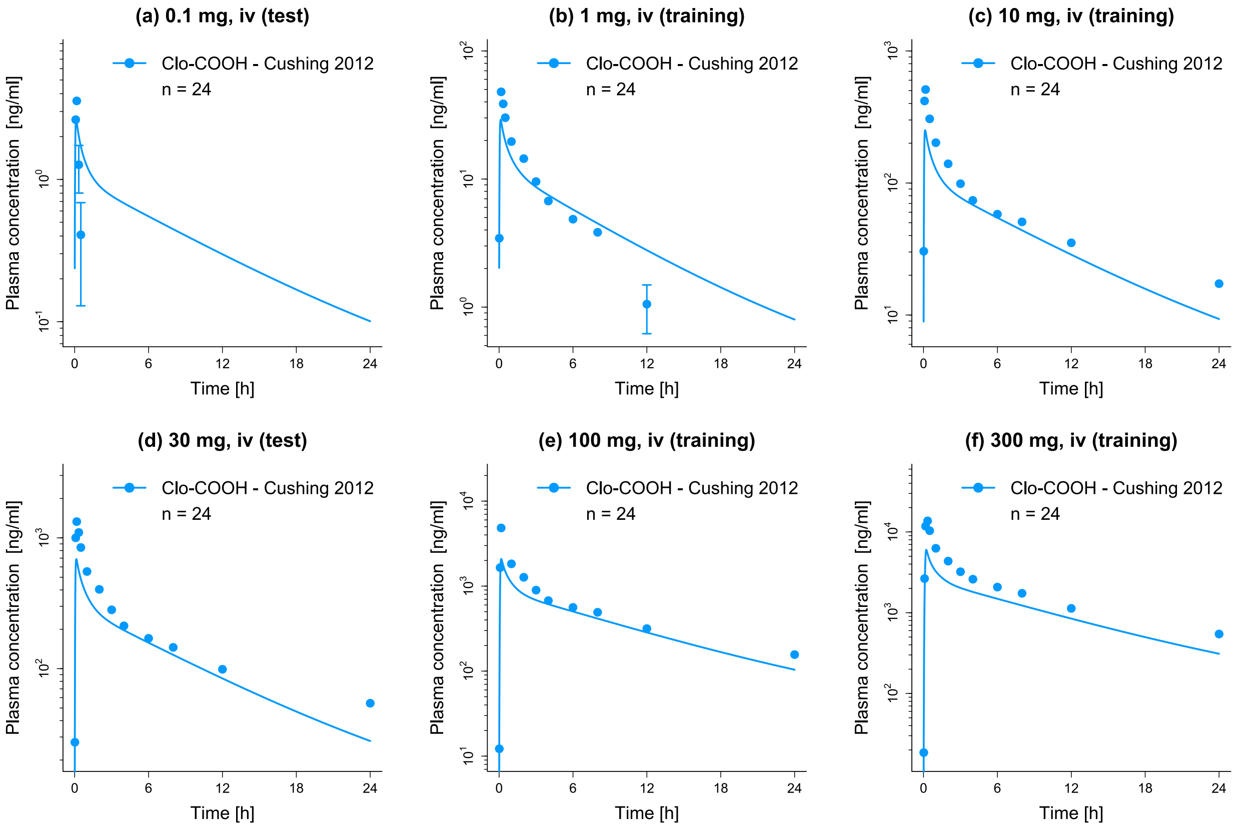

Figure S4: Semilogarithmic plots of predicted plasma concentration-time profiles of clopidogrel carboxylic acid following administration of clopidogrel. Solid lines represent the model predictions, while corresponding observed data are shown as symbols (± standard deviation, if available) [13]. Clo-COOH: clopidogrel carboxylic acid, iv: intravenous, n: number of participants.

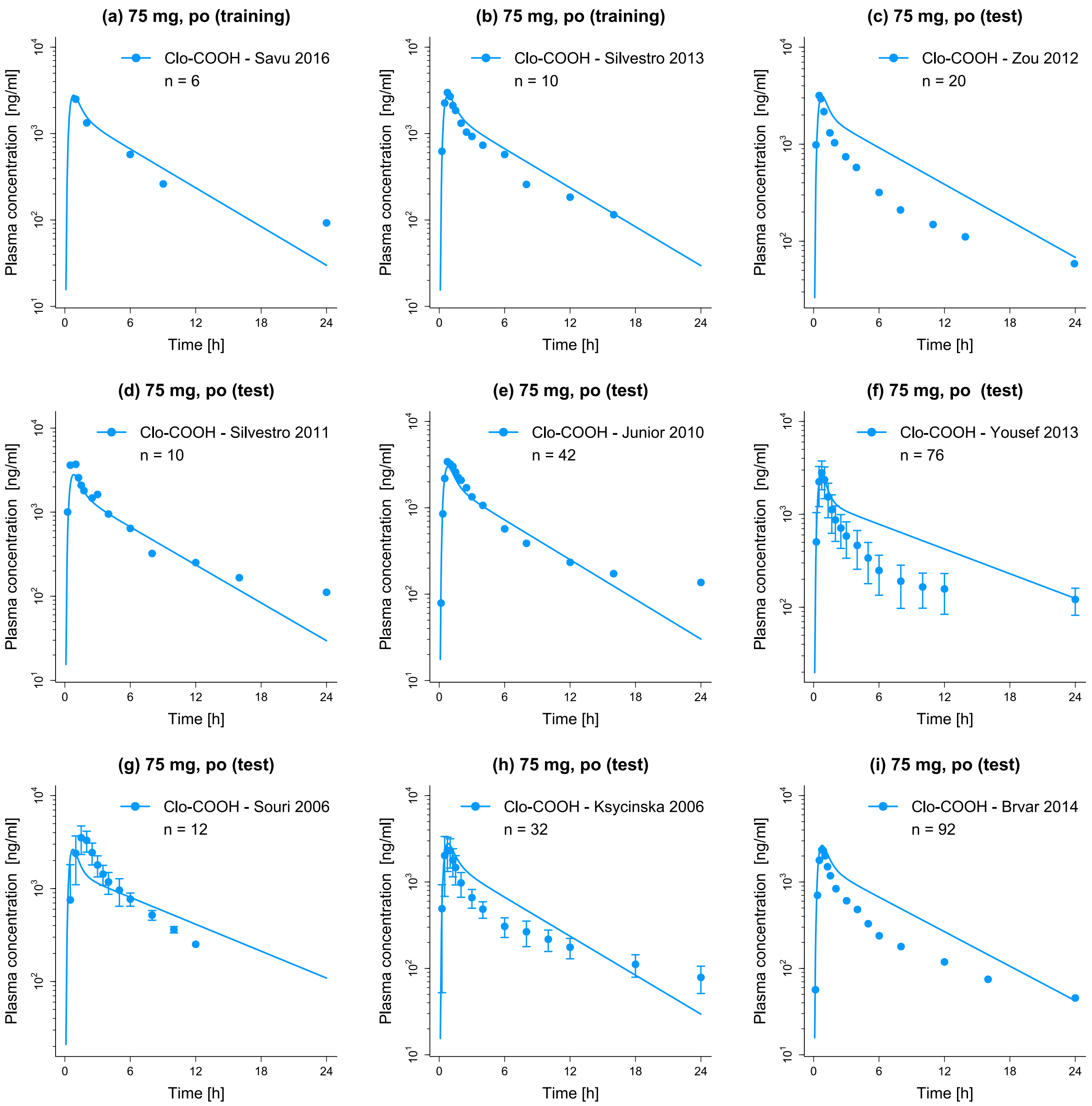

Figure S5: Semilogarithmic plots of predicted plasma concentration-time profiles of clopidogrel carboxylic acid following administration of clopidogrel. Solid lines represent the model predictions, while corresponding observed data are shown as symbols (± standard deviation, if available) [14, 15, 17, 19, 30–34]. Clo-COOH: clopidogrel carboxylic acid, n: number of participants, po: peroral.

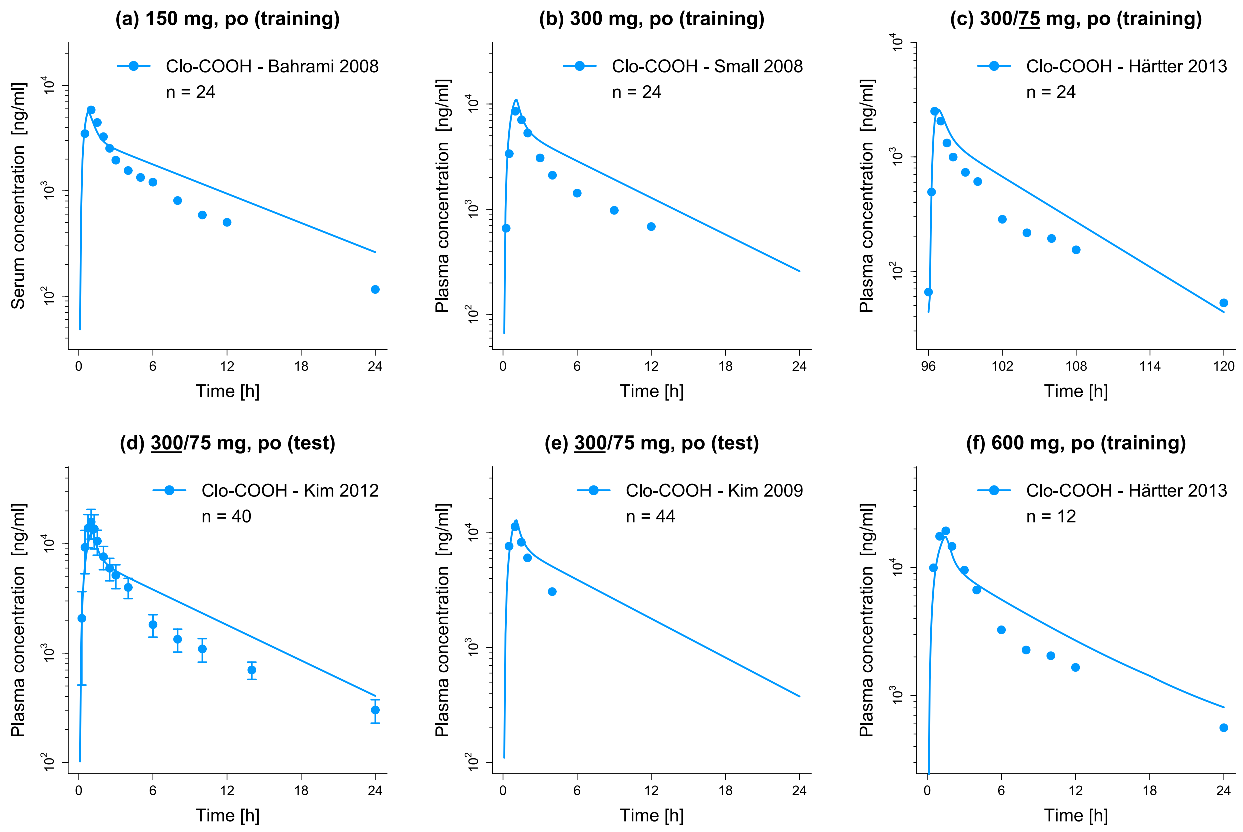

Figure S6: Semilogarithmic plots of predicted plasma concentration-time profiles of clopidogrel carboxylic acid following administration of clopidogrel. Solid lines represent the model predictions, while corresponding observed data are shown as symbols (± standard deviation, if available) [25, 27, 28, 35, 36]. Clo-COOH: clopidogrel carboxylic acid, n: number of participants, po: peroral.

S2.1.3 Clopidogrel Acyl Glucuronide

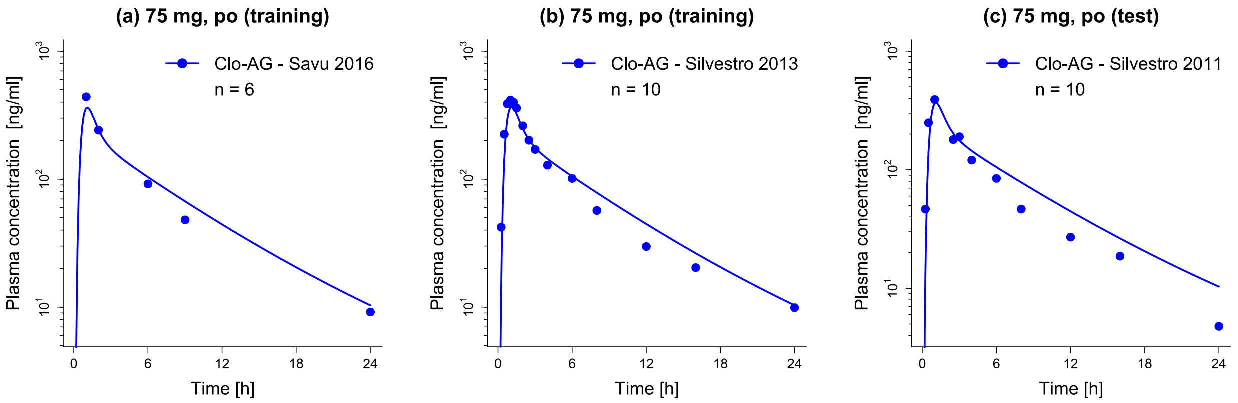

Figure S7: Semilogarithmic plots of predicted plasma concentration-time profiles of clopidogrel carboxylic acid following administration of clopidogrel. Solid lines represent the model predictions, while corresponding observed data are shown as symbols (± standard deviation, if available) [14, 15, 30]. Clo-AG: clopidogrel acyl glucuronide, n: number of participants, po: peroral.

S2.1.4 2-Oxo-Clopidogrel

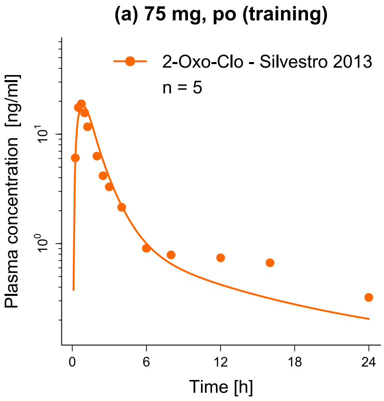

Figure S8: Semilogarithmic plots of predicted plasma concentration-time profiles of 2-oxo-clopidogrel following administration of clopidogrel. Solid lines represent the model predictions, while corresponding observed data are shown as symbols (± standard deviation, if available) [15]. 2-Oxo-Clo: 2-Oxo-clopidogrel, n: number of participants, po: peroral.

S2.1.5 Clopidogrel Thiol H4

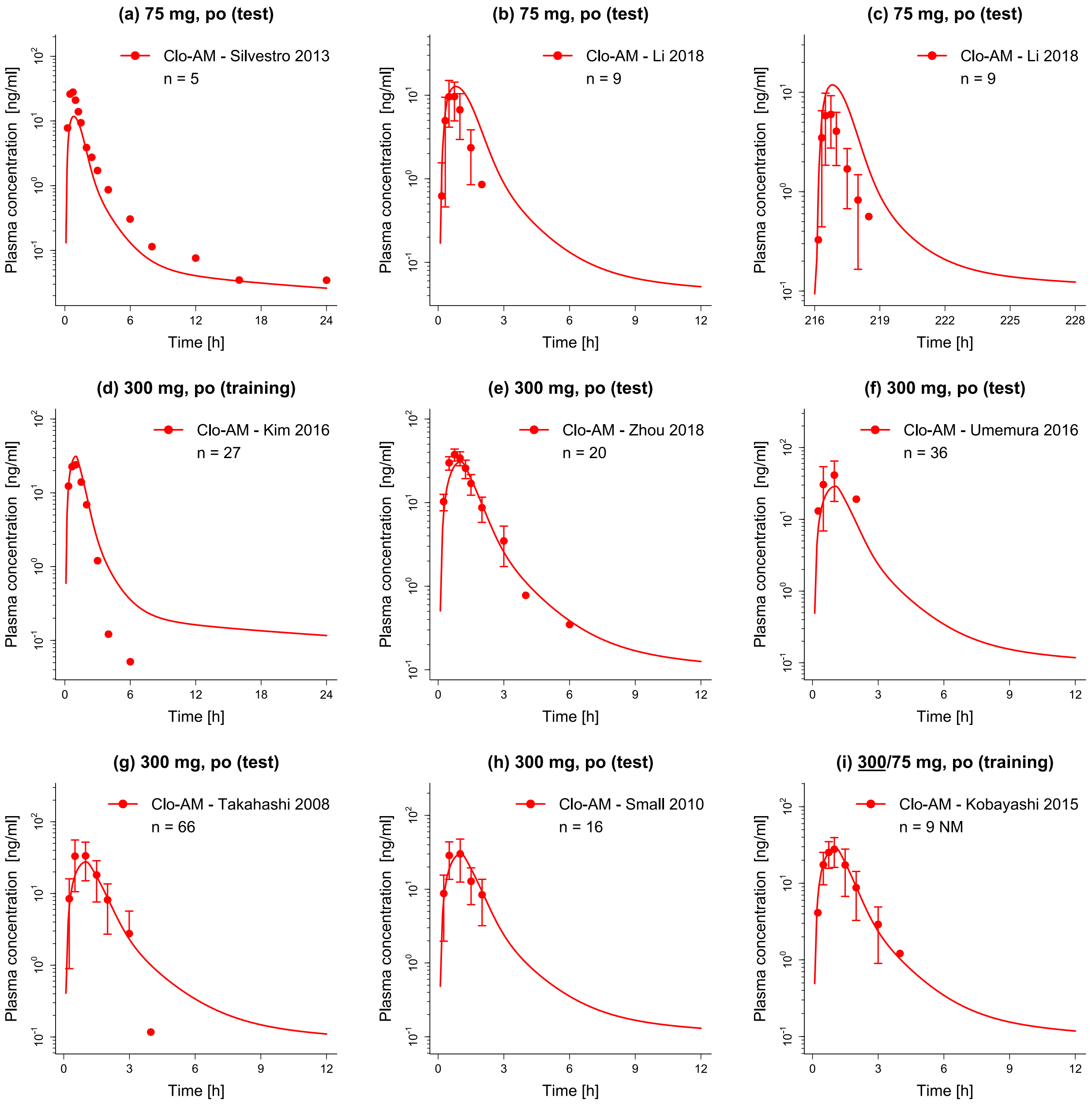

Figure S9: Semilogarithmic plots of predicted plasma concentration-time profiles of clopidogrel thiol H4 following administration of clopidogrel. Solid lines represent the model predictions, while corresponding observed data are shown as symbols (± standard deviation, if available) [15, 23, 24, 37–41]. Clo-AM: clopidogrel thiol H4, n: number of participants, NM: cytochrome P450 2C19 normal metabolizer, po: peroral.

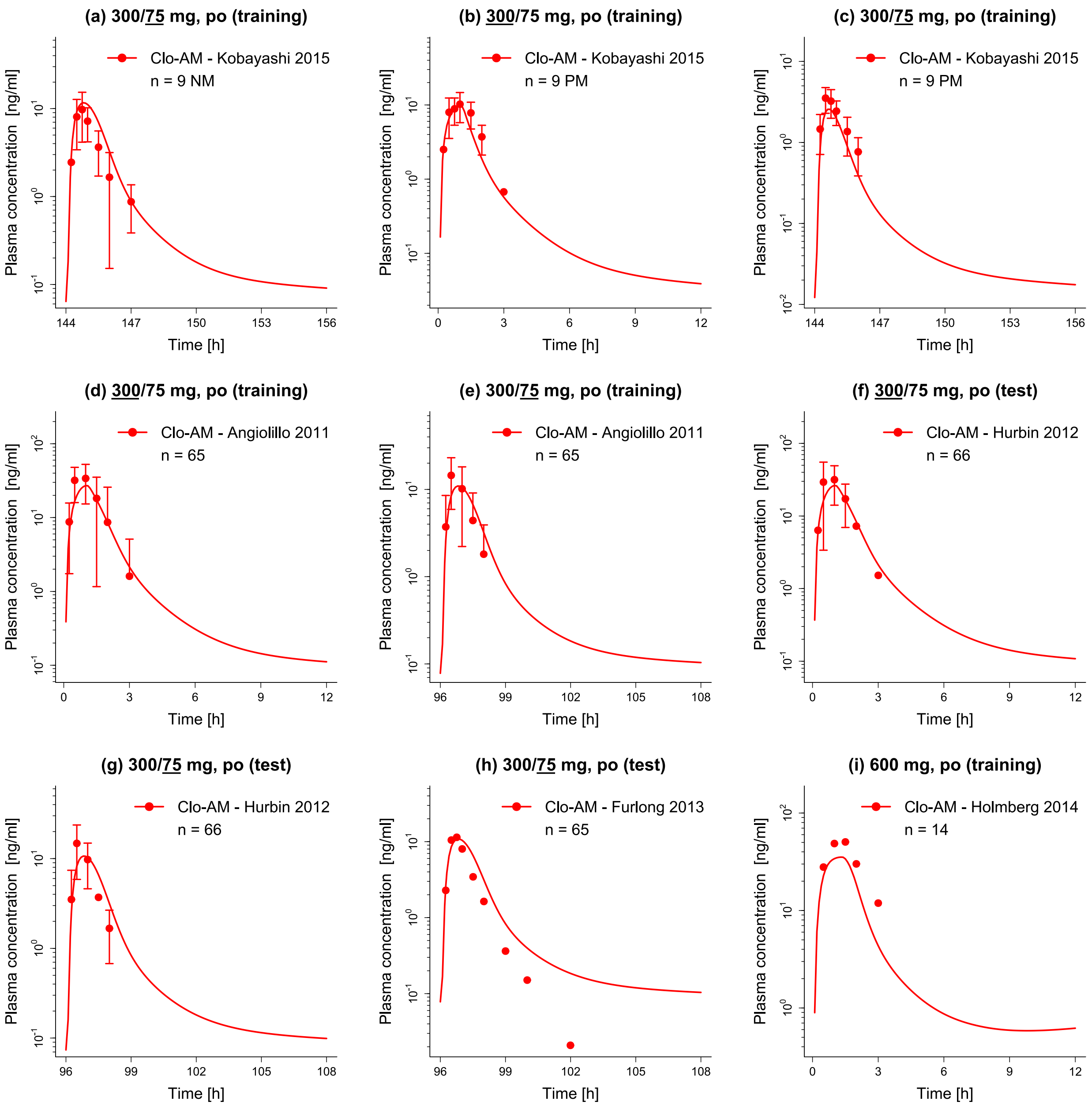

Figure S10: Semilogarithmic plots of predicted plasma concentration-time profiles of clopidogrel thiol H4 following administration of clopidogrel. Solid lines represent the model predictions, while corresponding observed data are shown as symbols (± standard deviation, if available) [29, 41–44]. Clo-AM: clopidogrel thiol H4, n: number of participants, NM: cytochrome P450 2C19 normal metabolizer, PM: cytochrome P450 2C19 poor metabolizer, po: peroral.

S2.2 Plasma Concentration-Time Profiles (Linear)

S2.2.1 Clopidogrel

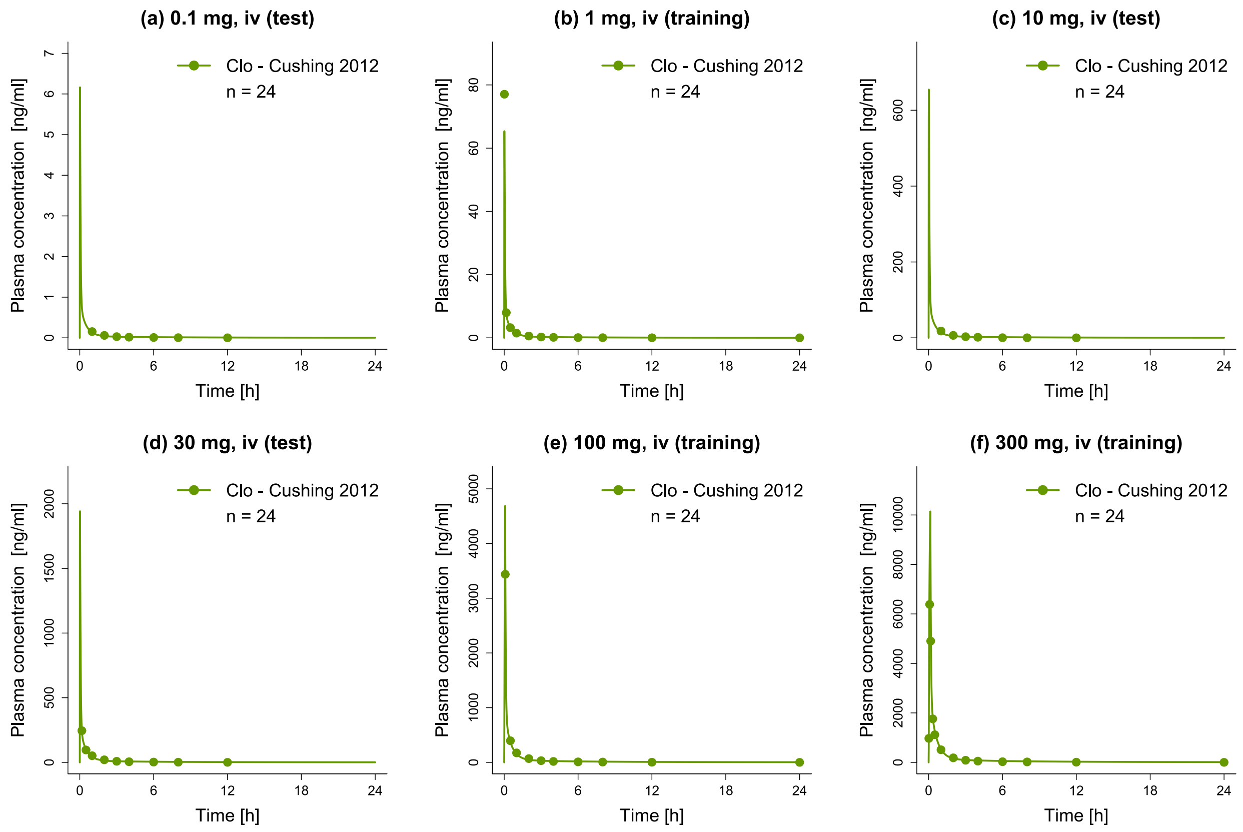

Figure S11: Linear plots of predicted plasma concentration-time profiles of clopidogrel. Solid lines represent the model predictions, while corresponding observed data are shown as symbols (± standard deviation, if available) [13]. Clo: clopidogrel, iv: intravenous, n: number of participants.

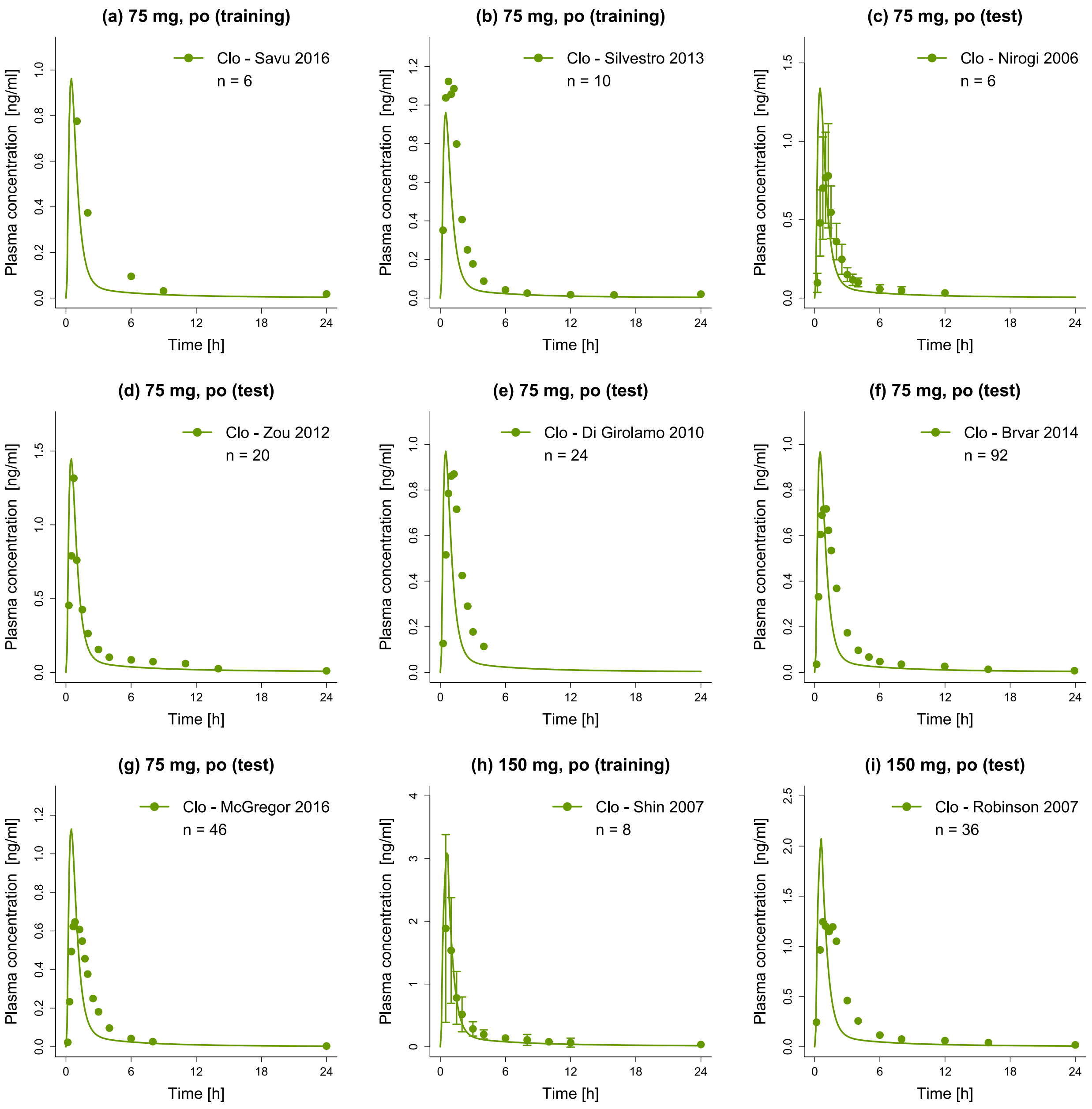

Figure S12: Linear plots of predicted plasma concentration-time profiles of clopidogrel. Solid lines represent the model predictions, while corresponding observed data are shown as symbols (± standard deviation, if available) [14–22]. Clo: clopidogrel, n: number of participants, po: peroral.

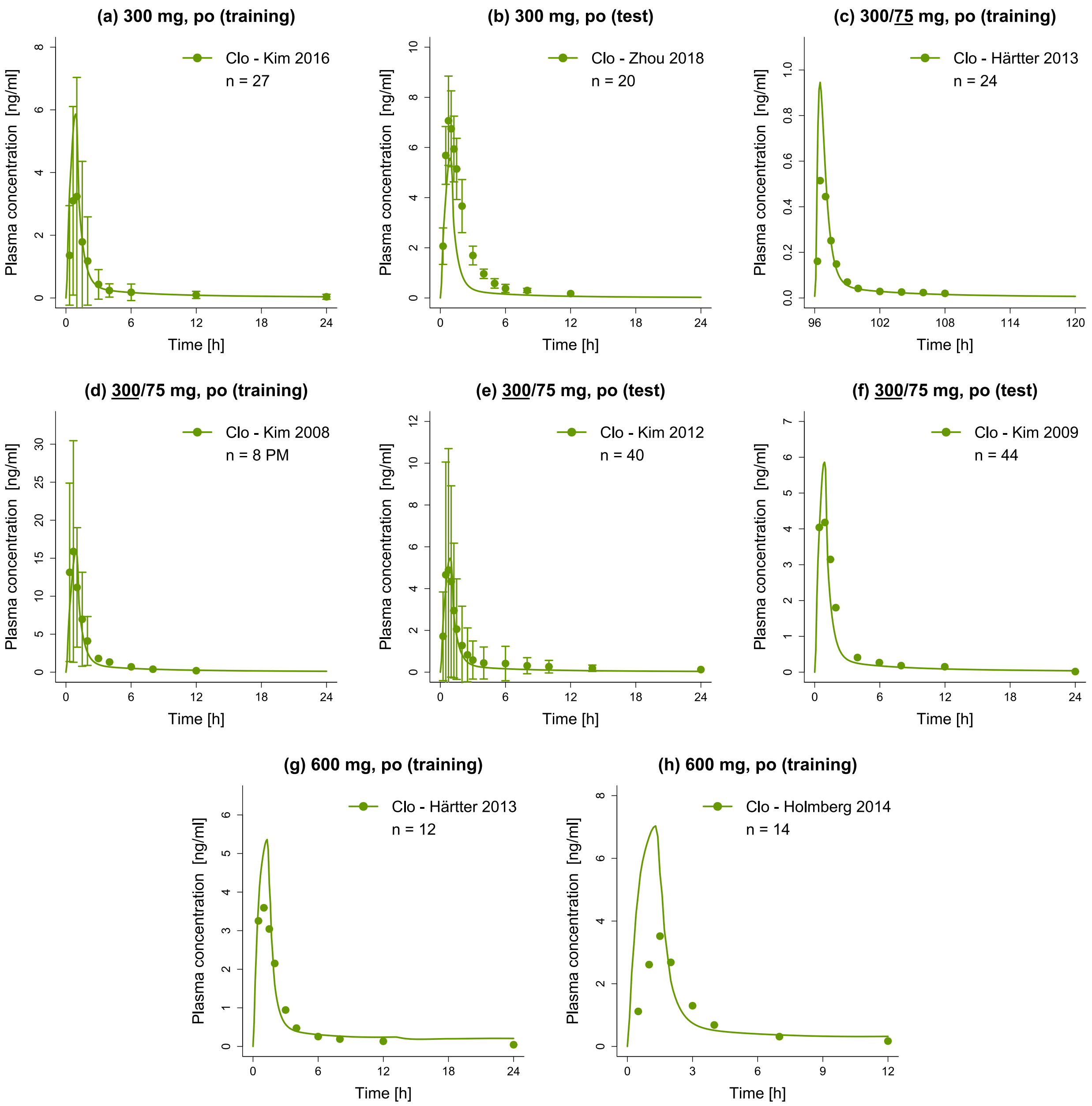

Figure S13: Linear plots of predicted plasma concentration-time profiles of clopidogrel. Solid lines represent the model predictions, while corresponding observed data are shown as symbols (± standard deviation, if available) [23–29]. Clo: clopidogrel, n: number of participants, PM: cytochrome P450 2C19 poor metabolizer, po: peroral.

S2.2.2 Clopidogrel Carboxylic Acid

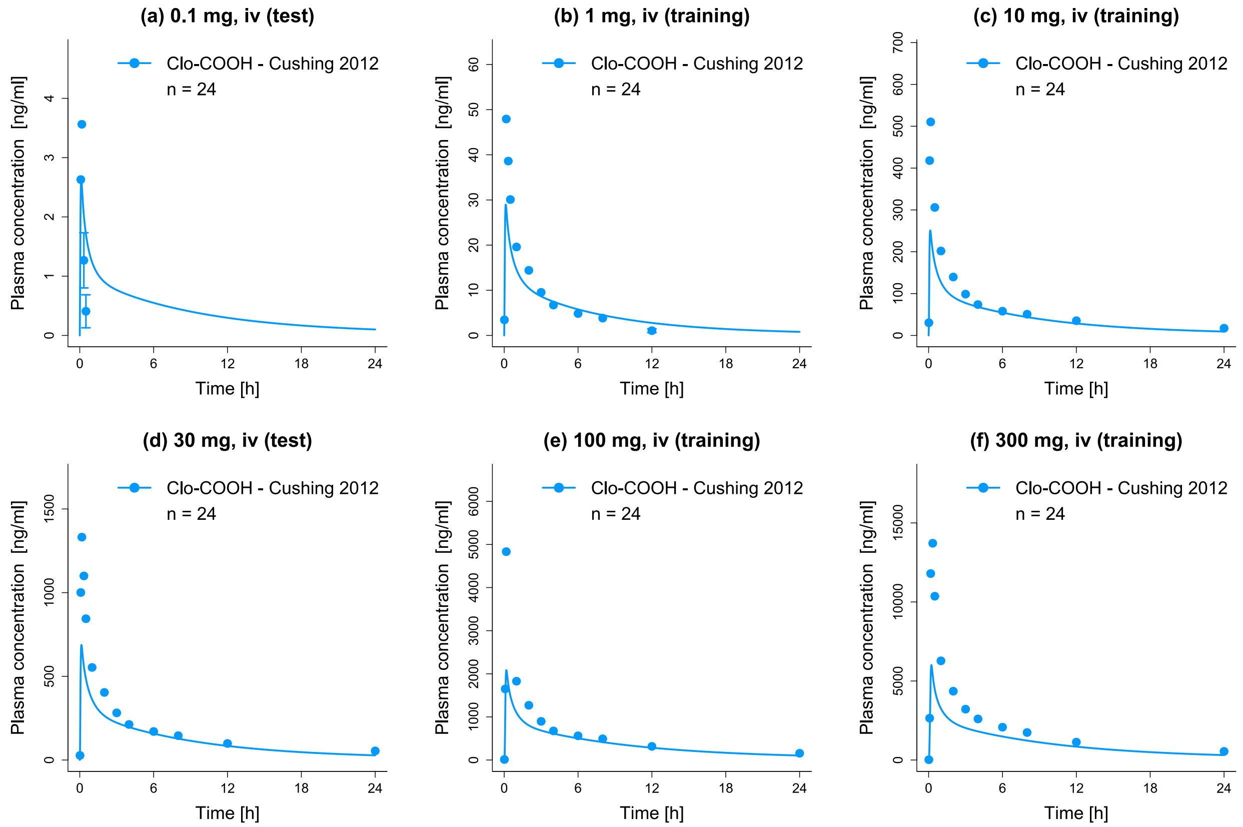

Figure S14: Linear plots of predicted plasma concentration-time profiles of clopidogrel carboxylic acid following administration of clopidogrel. Solid lines represent the model predictions, while corresponding observed data are shown as symbols (± standard deviation, if available) [13]. Clo-COOH: clopidogrel carboxylic acid, iv: intravenous, n: number of participants.

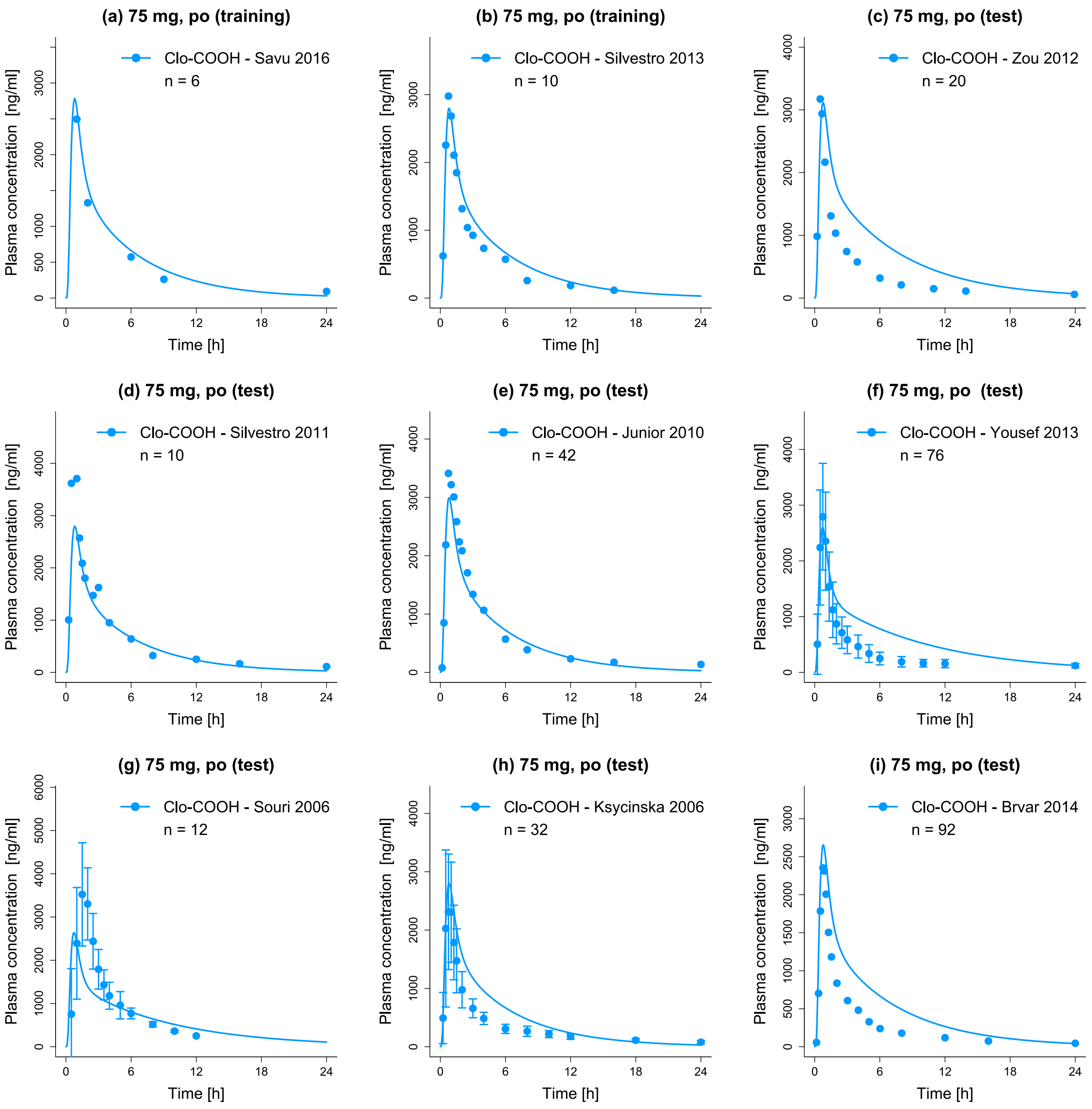

Figure S15: Linear plots of predicted plasma concentration-time profiles of clopidogrel carboxylic acid following administration of clopidogrel. Solid lines represent the model predictions, while corresponding observed data are shown as symbols (± standard deviation, if available) [14, 15, 17, 19, 30–34]. Clo-COOH: clopidogrel carboxylic acid, n: number of participants, po: peroral.

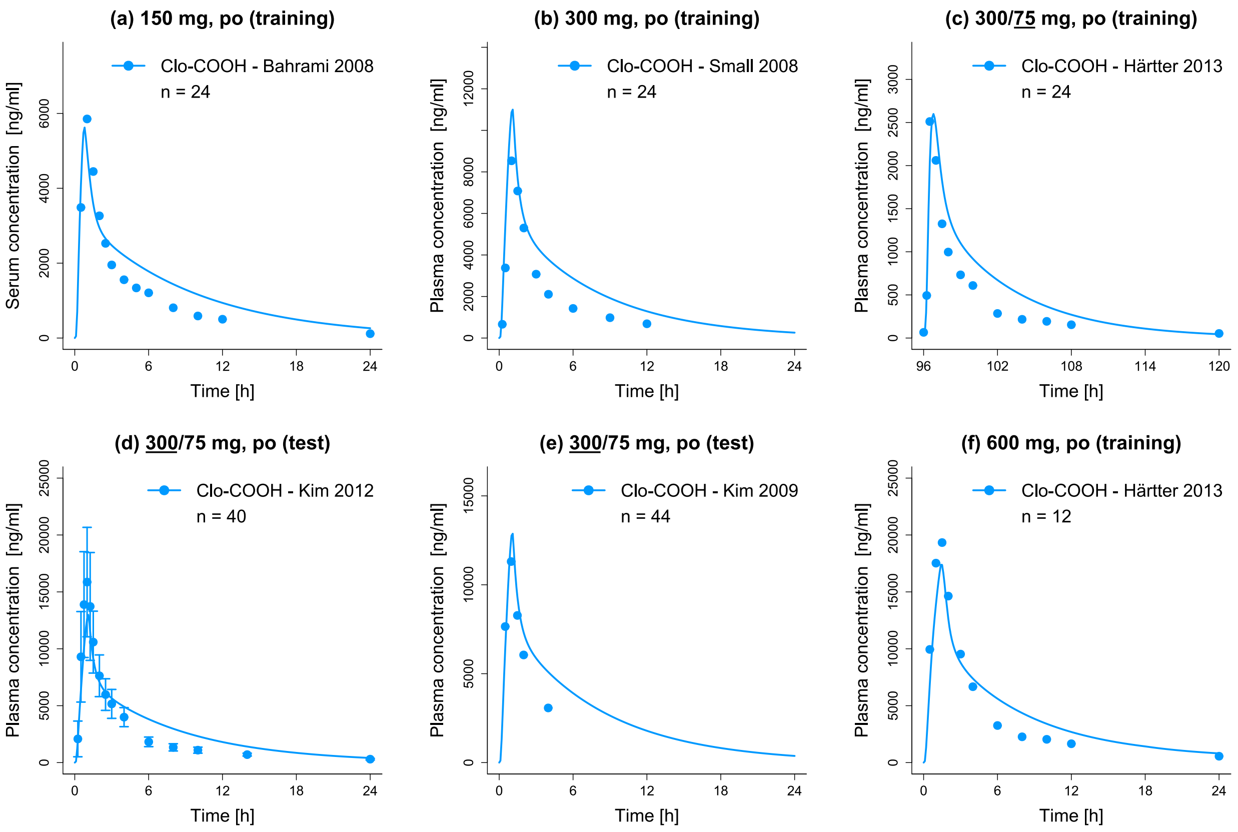

Figure S16: Linear plots of predicted plasma concentration-time profiles of clopidogrel carboxylic acid following administration of clopidogrel. Solid lines represent the model predictions, while corresponding observed data are shown as symbols (± standard deviation, if available) [25, 27, 28, 35, 36]. Clo-COOH: clopidogrel carboxylic acid, n: number of participants, po: peroral.

S2.2.3 Clopidogrel Acyl Glucuronide

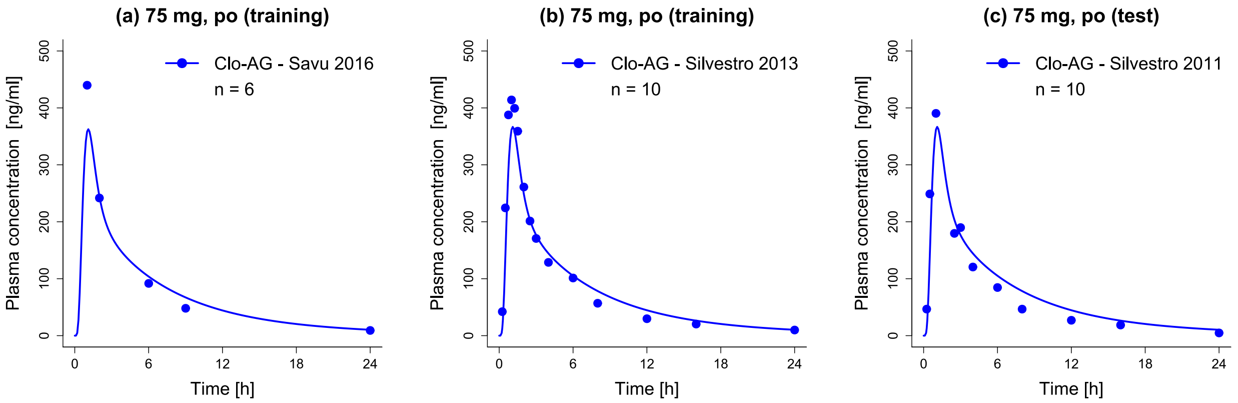

Figure S17: Linear plots of predicted plasma concentration-time profiles of clopidogrel carboxylic acid following administration of clopidogrel. Solid lines represent the model predictions, while corresponding observed data are shown as symbols (± standard deviation, if available) [14, 15, 30]. Clo-AG: clopidogrel acyl glucuronide, n: number of participants, po: peroral.

S2.2.4 2-Oxo-Clopidogrel

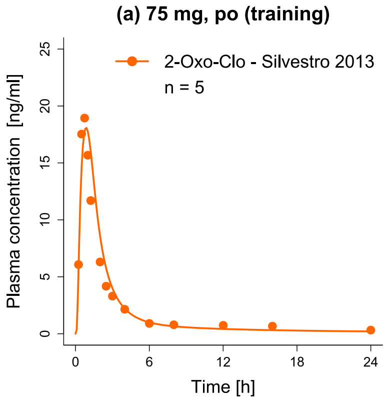

Figure S18: Linear plots of predicted plasma concentration-time profiles of 2-oxo-clopidogrel following administration of clopidogrel. Solid lines represent the model predictions, while corresponding observed data are shown as symbols (± standard deviation, if available) [15]. 2-Oxo-Clo: 2-Oxo-clopidogrel, n: number of participants, po: peroral.

S2.2.5 Clopidogrel Thiol H4

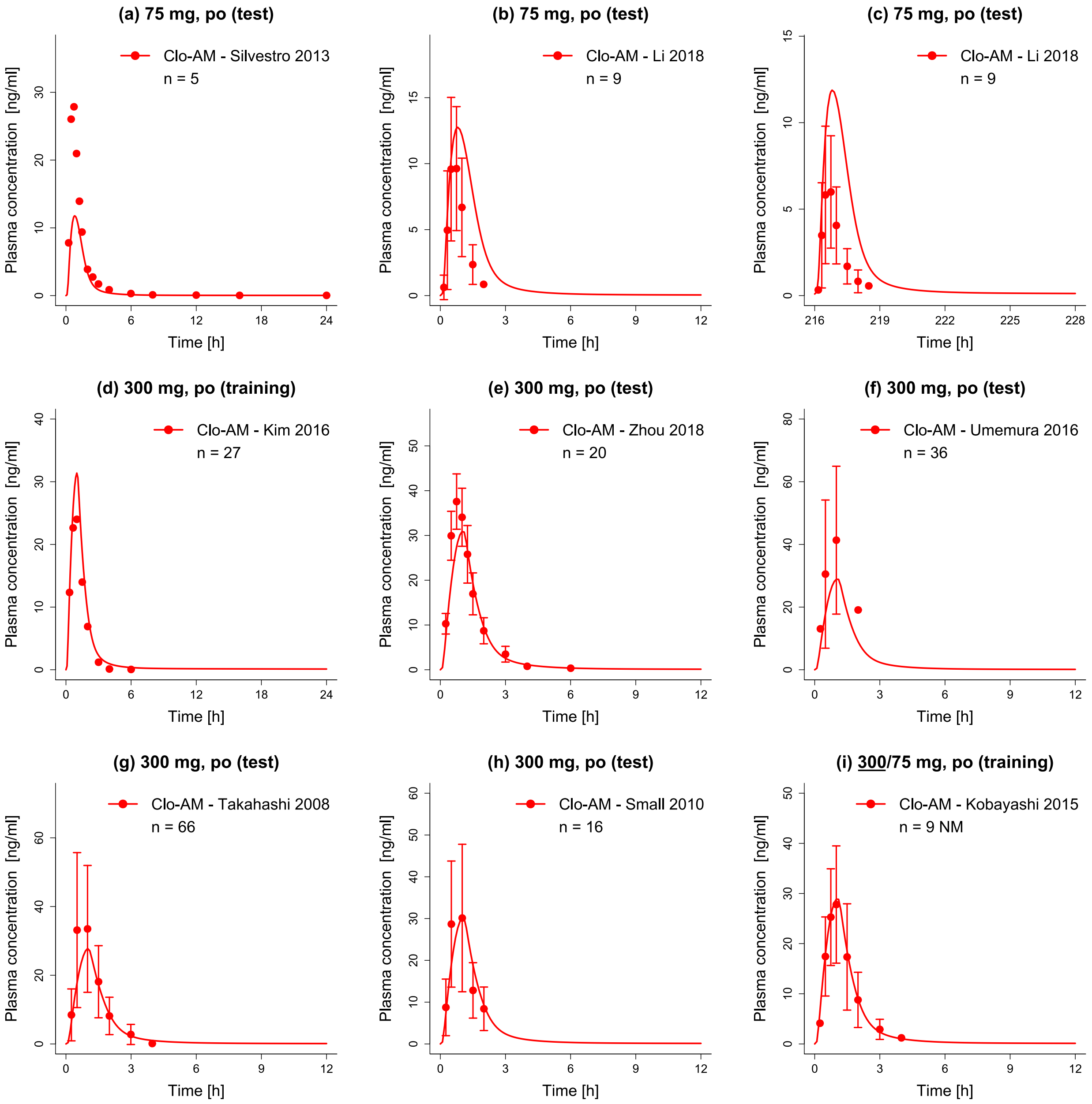

Figure S19: Linear plots of predicted plasma concentration-time profiles of clopidogrel thiol H4 following administration of clopidogrel. Solid lines represent the model predictions, while corresponding observed data are shown as symbols (± standard deviation, if available) [15, 23, 24, 37–41]. Clo-AM: clopidogrel thiol H4, n: number of participants, NM: cytochrome P450 2C19 normal metabolizer, po: peroral.

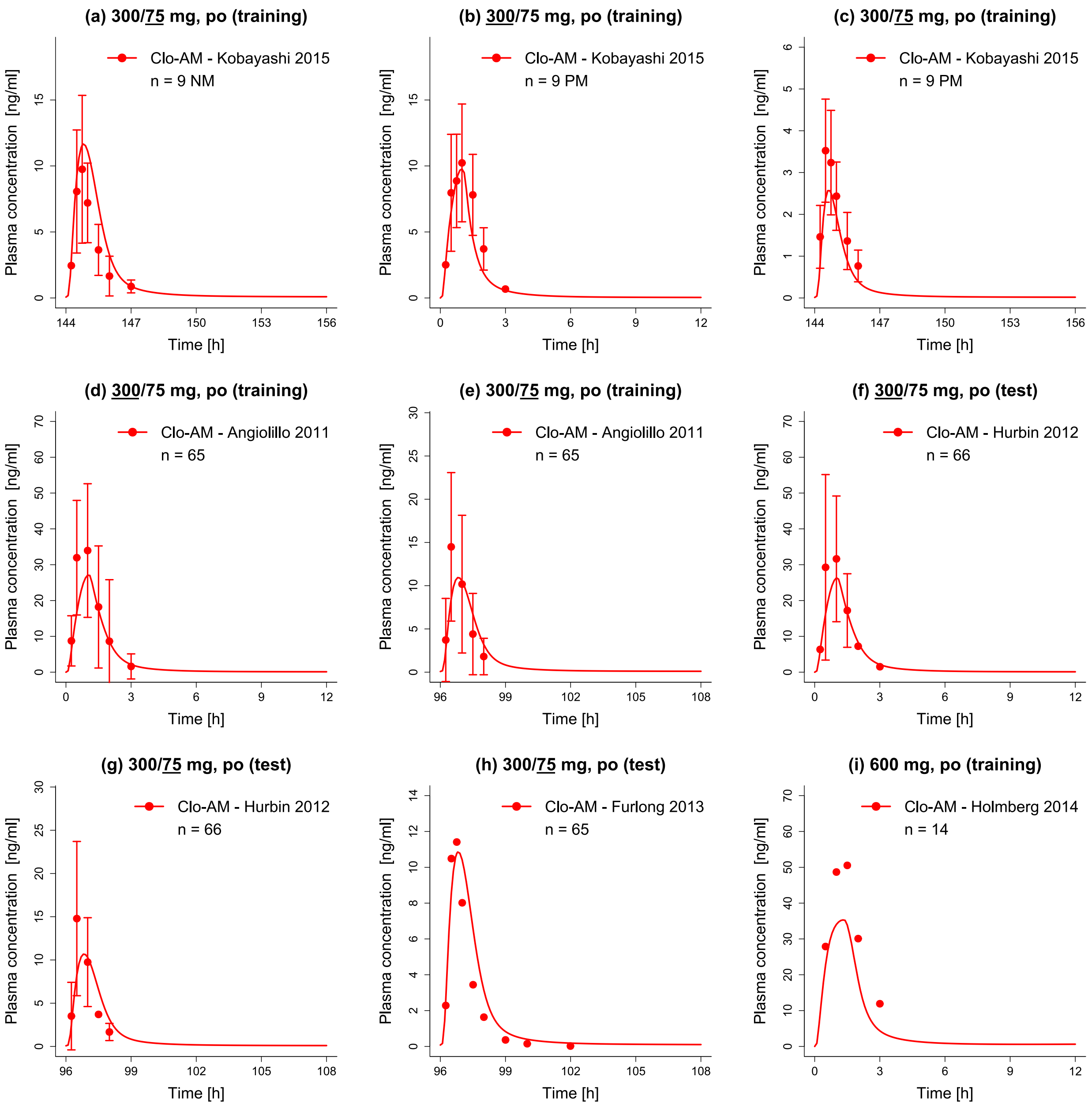

Figure S20: Linear plots of predicted plasma concentration-time profiles of clopidogrel thiol H4 following administration of clopidogrel. Solid lines represent the model predictions, while corresponding observed data are shown as symbols (± standard deviation, if available) [29, 41–44]. Clo-AM: clopidogrel thiol H4, n: number of participants, NM: cytochrome P450 2C19 normal metabolizer, PM: cytochrome P450 2C19 poor metabolizer, po: peroral.

S2.3 Plasma Concentration Goodness-of-Fit Plots

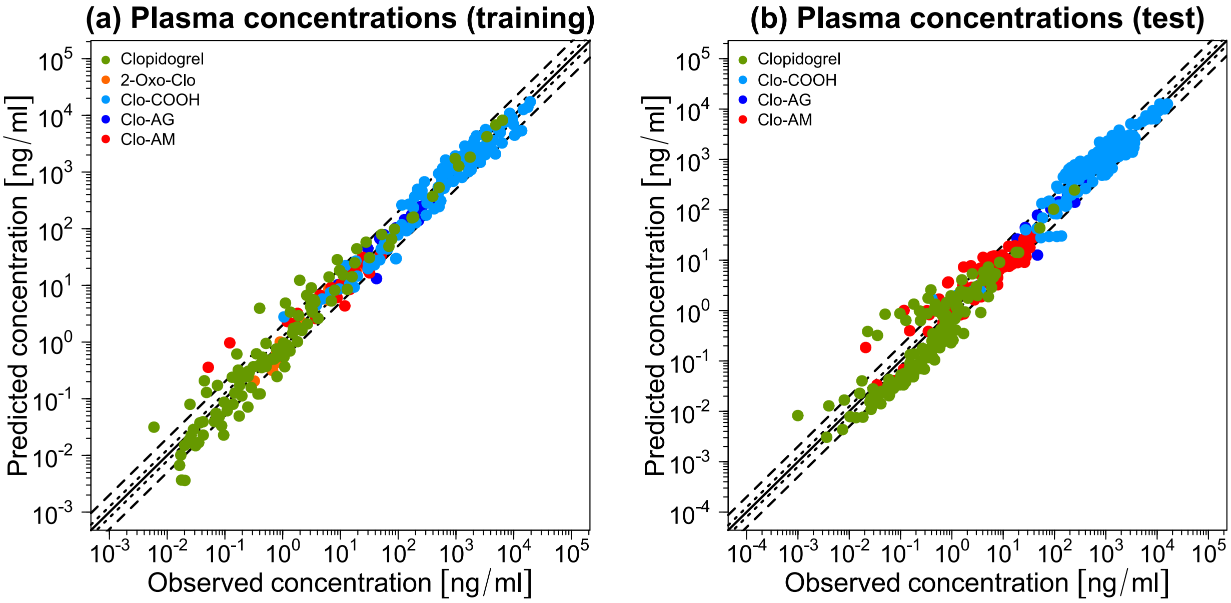

Figure S21: Plasma goodness-of-fit plots of the final clopidogrel parent-metabolite model. Divided by training and test dataset, each predicted plasma concentration measurement point is plotted against the corresponding observed value. The solid line represents the line of identity, while dotted lines indicate 1.25-fold and dashed lines 2-fold deviation from the respective observed value. 2-Oxo-Clo: 2-Oxo-clopidogrel, Clo-AM: clopidogrel thiol H4, Clo-AG: clopidogrel acyl glucuronide, Clo-COOH: clopidogrel carboxylic acid.

S2.4 AUC_{last} and C_{max} Goodness-of-Fit Plots

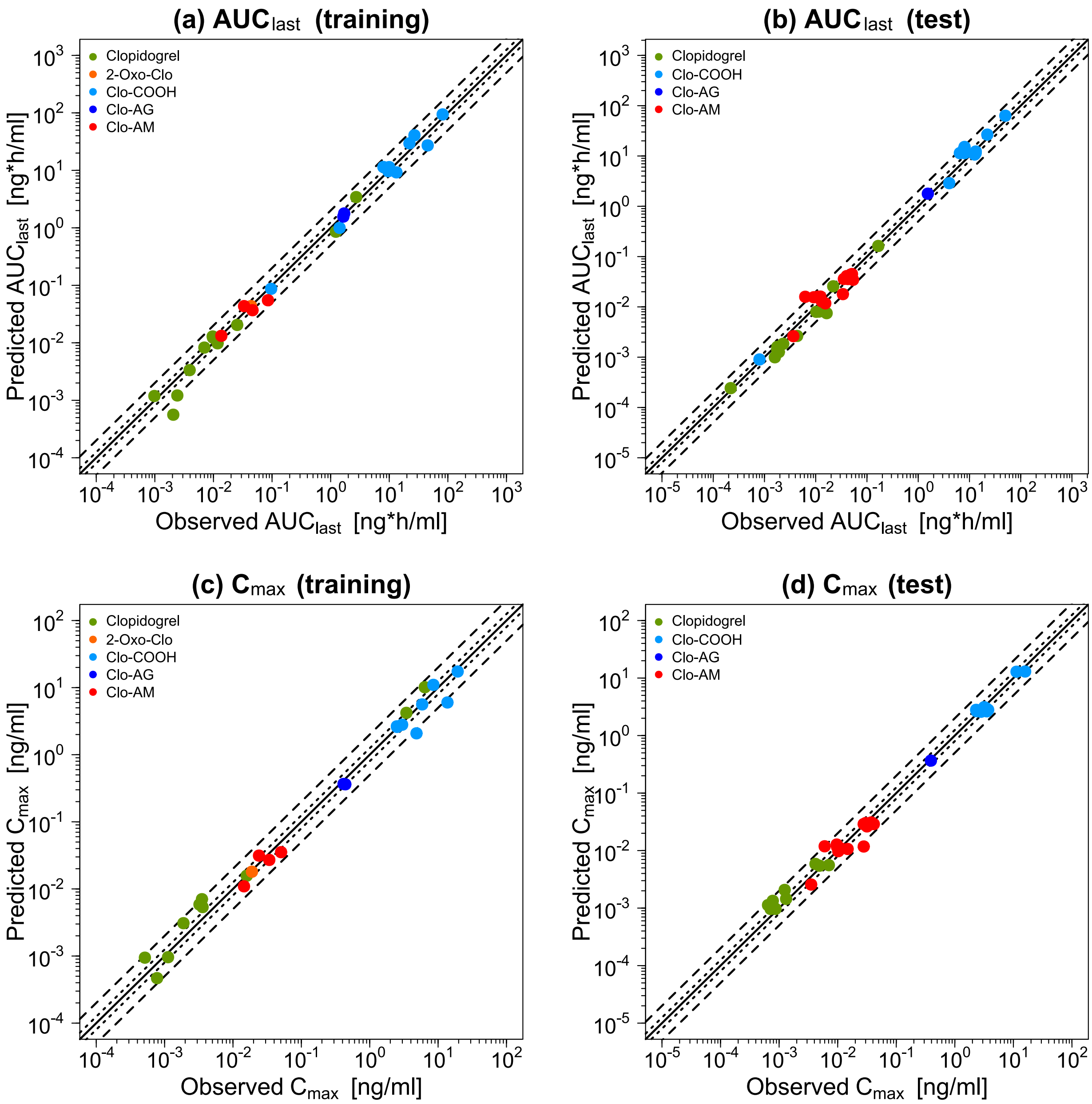

Figure S22: AUC_{last} and C_{max} goodness-of-fit plots of the final clopidogrel parent-metabolite model. Divided by training and test dataset, predicted (a–b) AUC_{last} and (c–d) C_{max} values are plotted against their corresponding observed counterparts. The solid line represents the line of identity, while dotted lines indicate 1.25-fold and dashed lines 2-fold deviation from the respective observed value. 2-Oxo-Clo: 2-Oxo-clopidogrel, AUC_{last}: area under the plasma concentration-time curve determined between first and last concentration measurements, Clo-AM: clopidogrel thiol H4, Clo-AG: clopidogrel acyl glucuronide, Clo-COOH: clopidogrel carboxylic acid, C_{max}: maximum plasma concentration.

S2.5 MRD of Plasma Concentration Predictions

Table S5: MRD values of plasma concentration predictions

Compound	Clopidogrel dosing regimen		n	Dataset	MRD	Reference
	Route	Dose [mg]				
Clo	iv (bolus, s.d.)	0.1	24	test	2.65	Cushing 2012 [13]
Clo	iv (bolus, s.d.)	1	24	training	2.11	Cushing 2012 [13]
Clo	iv (bolus, s.d.)	10	24	test	4.34	Cushing 2012 [13]
Clo	iv (bolus, s.d.)	30	24	test	2.16	Cushing 2012 [13]
Clo	iv (inf, 4 min, s.d.)	100	24	training	2.41	Cushing 2012 [13]
Clo	iv (inf, 8 min, s.d.)	300	24	training	2.05	Cushing 2012 [13]
Clo	po (tab, s.d.)	75	6	training	3.12	Savu 2016 [14]
Clo	po (-, s.d.)	75	10	training	2.59	Silvestro 2013 [15]
Clo	po (-, s.d.)	75	6	test	2.42	Nirogi 2006 [16]
Clo	po (tab, s.d.)	75	20	test	1.90	Zou 2012 [17]
Clo	po (tab, s.d.)	75	24	test	3.00	Di Girolamo 2010 [18]
Clo	po (tab, s.d.)	75	92	test	2.52	Brvar 2014 [19]
Clo	po (tab, s.d.)	75	46	test	2.94	McGregor 2016 [20]
Clo	po (tab, s.d.)	150	8	training	1.64	Shin 2007 [21]
Clo	po (tab, s.d.)	150	36	test	2.63	Robinson 2007 [22]
Clo	po (tab, s.d.)	300	27	training	1.49	Kim 2016 [23]
Clo	po (tab, s.d.)	300	20	test	2.64	Zhou 2018 [24]
Clo	po (-, l.d./m.d., 5d)	300/75	24	training	1.61 (D5)	Härtter 2013 [25]
Clo	po (-, l.d./m.d., 7d)	300/75	8 PM	training	1.48 (D1)	Kim 2008 [26]
Clo	po (-, l.d./m.d., 5d)	300/75	40	test	1.91 (D1)	Kim 2012 [27]
Clo	po (-, l.d./m.d., 6d)	300/75	44	test	1.64 (D1)	Kim 2009 [28]
Clo	po (-, s.d.)	600	12	training	1.80	Härtter 2013 [25]
Clo	po (tab, s.d.)	600	14	training	2.05	Holmberg 2014 [29]
Clo-COOH	iv (bolus, s.d.)	0.1	24	test	2.17	Cushing 2012 [13]
Clo-COOH	iv (bolus, s.d.)	1	24	training	1.54	Cushing 2012 [13]
Clo-COOH	iv (bolus, s.d.)	10	24	training	1.54	Cushing 2012 [13]
Clo-COOH	iv (bolus, s.d.)	30	24	test	1.56	Cushing 2012 [13]
Clo-COOH	iv (inf, 4 min, s.d.)	100	24	training	1.55	Cushing 2012 [13]
Clo-COOH	iv (inf, 8 min, s.d.)	300	24	training	1.80	Cushing 2012 [13]
Clo-COOH	po (tab, s.d.)	75	6	training	1.73	Savu 2016 [14]
Clo-COOH	po (-, s.d.)	75	10	training	1.44	Silvestro 2013 [15]
Clo-COOH	po (tab, s.d.)	75	20	test	2.09	Zou 2012 [17]
Clo-COOH	po (-, s.d.)	75	10	test	1.59	Silvestro 2011 [30]
Clo-COOH	po (tab, s.d.)	75	42	test	1.55	Junior 2010 [31]
Clo-COOH	po (tab, s.d.)	75	76	test	1.98	Yousef 2013 [32]
Clo-COOH	po (tab, s.d.)	75	12	test	1.69	Souri 2006 [33]
Clo-COOH	po (tab, s.d.)	75	32	test	1.62	Ksycinska 2006 [34]
Clo-COOH	po (tab, s.d.)	75	92	test	1.84	Brvar 2014 [19]

-: not given, 2-Oxo-Clo: 2-Oxo-clopidogrel, Clo: clopidogrel, Clo-AG: clopidogrel acyl glucuronide, Clo-AM: clopidogrel thiol H4, Clo-COOH: clopidogrel carboxylic acid, d: dosage period in days, D: day of pharmacokinetic sampling, inf: infusion, iv: intravenous, l.d.: loading dose, m.d.: maintenance dose (once daily), MRD: mean relative deviation, n: number of participants, NM: cytochrome P450 2C19 normal metabolizer, PM: cytochrome P450 2C19 poor metabolizer, po: peroral, s.d.: single dose, tab: tablet.

Table S5: MRD values of plasma concentration predictions *(continued)*

Compound	Clopidogrel dosing regimen		n	Dataset	MRD	Reference
	Route	Dose [mg]				
Clo-COOH	po (tab, s.d.)	150	24	training	1.54	Bahrami 2008 [35]
Clo-COOH	po (tab, s.d.)	300	24	training	1.72	Small 2008 [36]
Clo-COOH	po (-, l.d./m.d., 5d)	300/75	24	training	1.62 (D5)	Härtter 2013 [25]
Clo-COOH	po (-, l.d./m.d., 5d)	300/75	40	test	1.53 (D1)	Kim 2012 [27]
Clo-COOH	po (-, l.d./m.d., 6d)	300/75	44	test	1.30 (D1)	Kim 2009 [28]
Clo-COOH	po (-, s.d.)	600	12	training	1.46	Härtter 2013 [25]
Clo-AG	po (tab, s.d.)	75	6	training	1.21	Savu 2016 [14]
Clo-AG	po (-, s.d.)	75	10	training	1.45	Silvestro 2013 [15]
Clo-AG	po (-, s.d.)	75	10	test	1.74	Silvestro 2011 [30]
2-Oxo-Clo	po (-, s.d.)	75	5	training	1.39	Silvestro 2013 [15]
Clo-AM	po (-, s.d.)	75	5	test	1.89	Silvestro 2013 [15]
Clo-AM	po (-, m.d., 10d)	75	9	test	2.23 (D1)	Li 2018 [37]
Clo-AM	po (-, m.d., 10d)	75	9	test	2.83 (D10)	Li 2018 [37]
Clo-AM	po (tab, s.d.)	300	27	training	2.85	Kim 2016 [23]
Clo-AM	po (tab, s.d.)	300	20	test	1.30	Zhou 2018 [24]
Clo-AM	po (tab, s.d.)	300	36	test	1.76	Umemura 2016 [38]
Clo-AM	po (tab, s.d.)	300	66	test	2.36	Takahashi 2008 [39]
Clo-AM	po (tab, s.d.)	300	16	test	1.31	Small 2010 [40]
Clo-AM	po (-, l.d./m.d., 7d)	300/75	9 NM	training	1.21 (D1)	Kobayashi 2015 [41]
Clo-AM	po (-, l.d./m.d., 7d)	300/75	9 NM	training	1.50 (D7)	Kobayashi 2015 [41]
Clo-AM	po (-, l.d./m.d., 7d)	300/75	9 PM	training	1.41 (D1)	Kobayashi 2015 [41]
Clo-AM	po (-, l.d./m.d., 7d)	300/75	9 PM	training	1.57 (D5)	Kobayashi 2015 [41]
Clo-AM	po (-, l.d./m.d., 5d)	300/75	65	training	1.41 (D1)	Angiolillo 2011 [42]
Clo-AM	po (-, l.d./m.d., 5d)	300/75	65	training	1.54 (D5)	Angiolillo 2011 [42]
Clo-AM	po (-, l.d./m.d., 5d)	300/75	66	test	1.34 (D1)	Hurbin 2012 [43]
Clo-AM	po (-, l.d./m.d., 5d)	300/75	66	test	1.63 (D5)	Hurbin 2012 [43]
Clo-AM	po (-, l.d./m.d., 5d)	300/75	65	test	2.47 (D5)	Furlong 2013 [44]
Clo-AM	po (tab, s.d.)	600	14	training	1.75	Holmberg 2014 [29]
			Overall mean MRD (range)		1.91 (1.21–4.34)	
			MRD ≤ 2		44/66	

-: not given, 2-Oxo-Clo: 2-Oxo-clopidogrel, Clo: clopidogrel, Clo-AG: clopidogrel acyl glucuronide, Clo-AM: clopidogrel thiol H4, Clo-COOH: clopidogrel carboxylic acid, d: dosage period in days, D: day of pharmacokinetic sampling, inf: infusion, iv: intravenous, l.d.: loading dose, m.d.: maintenance dose (once daily), MRD: mean relative deviation, n: number of participants, NM: cytochrome P450 2C19 normal metabolizer, PM: cytochrome P450 2C19 poor metabolizer, po: peroral, s.d.: single dose, tab: tablet.

S2.6 Predicted and Observed AUC_{last} and C_{max} Values with Mean GMFEs

Table S6: Predicted versus observed AUC_{last} and C_{max} values, along with GMFE values

Compound	Clopidogrel dosing regimen		n	Dataset	AUC_{last}			C_{max}			Reference
	Route	Dose [mg]			Pred $[\frac{ng*h}{ml}]$	Obs $[\frac{ng*h}{ml}]$	Pred/Obs	Pred $[\frac{ng}{ml}]$	Obs $[\frac{ng}{ml}]$	Pred/Obs	
Clo	iv (bolus, s.d.)	0.1	24	test	0.24	0.22	1.10	-	-	-	Cushing 2012 [13]
Clo	iv (bolus, s.d.)	1	24	training	9.80	11.78	0.83	-	-	-	Cushing 2012 [13]
Clo	iv (bolus, s.d.)	10	24	test	25.68	21.96	1.17	-	-	-	Cushing 2012 [13]
Clo	iv (bolus, s.d.)	30	24	test	162.68	166.20	0.98	-	-	-	Cushing 2012 [13]
Clo	iv (inf, 4 min, s.d.)	100	24	training	855.37	1226.90	0.70	4210.44	3437.8	1.22	Cushing 2012 [13]
Clo	iv (inf, 8 min, s.d.)	300	24	training	3429.21	2726.75	1.26	10143.06	6385	1.59	Cushing 2012 [13]
Clo	po (tab, s.d.)	75	6	training	0.56	2.06	0.27	0.47	0.78	0.60	Savu 2016 [14]
Clo	po (-, s.d.)	75	10	training	1.21	2.42	0.50	0.96	1.12	0.86	Silvestro 2013 [15]
Clo	po (-, s.d.)	75	6	test	1.60	1.78	0.90	1.34	0.78	1.72	Nirogi 2006 [16]
Clo	po (tab, s.d.)	75	20	test	1.85	2.32	0.80	1.45	1.32	1.10	Zou 2012 [17]
Clo	po (tab, s.d.)	75	24	test	0.99	1.59	0.62	0.97	0.87	1.11	Di Girolamo 2010 [18]
Clo	po (tab, s.d.)	75	92	test	1.27	1.91	0.66	0.97	0.72	1.35	Brvar 2014 [19]
Clo	po (tab, s.d.)	75	46	test	1.42	1.77	0.80	1.13	0.65	1.74	McGregor 2016 [20]
Clo	po (tab, s.d.)	150	8	training	3.35	3.92	0.85	3.09	1.89	1.64	Shin 2007 [21]
Clo	po (tab, s.d.)	150	36	test	2.64	4.34	0.61	2.07	1.25	1.66	Robinson 2007 [22]
Clo	po (tab, s.d.)	300	27	training	8.26	7.00	1.18	5.87	3.23	1.81	Kim 2016 [23]
Clo	po (tab, s.d.)	300	20	test	7.50	16.34	0.46	5.58	7.07	0.79	Zhou 2018 [24]
Clo	po (-, l.d./m.d., 5d)	300/75	24	training	1.19	0.98	1.21 (D5)	0.95	0.51	1.84 (D5)	Härtter 2013 [25]
Clo	po (-, l.d./m.d., 7d)	300/75	8 PM	training	20.46	25.42	0.80 (D1)	15.75	15.88	0.99 (D1)	Kim 2008 [26]
Clo	po (-, l.d./m.d., 5d)	300/75	40	test	8.04	11.53	0.70 (D1)	5.45	4.89	1.12 (D1)	Kim 2012 [27]
Clo	po (-, l.d./m.d., 6d)	300/75	44	test	7.98	10.26	0.78 (D1)	5.86	4.18	1.40 (D1)	Kim 2009 [28]
Clo	po (-, s.d.)	600	12	training	12.31	9.82	1.25	5.36	3.59	1.49	Härtter 2013 [25]
Clo	po (tab, s.d.)	600	14	training	12.90	9.70	1.33	7.03	3.52	2.00	Holmberg 2014 [29]

-: not given, 2-Oxo-Clo: 2-Oxo-clopidogrel, AUC_{last}: area under the plasma concentration-time curve determined between first and last concentration measurements, Clo: clopidogrel, Clo-AG: clopidogrel acyl glucuronide, Clo-AM: clopidogrel thiol H4, Clo-COOH: clopidogrel carboxylic acid, C_{max}: maximum plasma concentration, d: dosage period in days, D: day of pharmacokinetic sampling, inf: infusion, GMFE: geometric mean fold error, iv: intravenous, l.d.: loading dose, m.d.: maintenance dose (once daily), n: number of participants, NM: cytochrome P450 2C19 normal metabolizer, obs: observed, PM: cytochrome P450 2C19 poor metabolizer, po: peroral, pred: predicted, s.d.: single dose, tab: tablet.

Table S6: Predicted versus observed AUC_{last} and C_{max} values, along with GMFE values *(continued)*

Compound	Clopidogrel dosing regimen		n	Dataset	AUC_{last}			C_{max}			Reference
	Route	Dose [mg]			Pred [$\frac{ng*h}{ml}$]	Obs [$\frac{ng*h}{ml}$]	Pred/Obs	Pred [$\frac{ng}{ml}$]	Obs [$\frac{ng}{ml}$]	Pred/Obs	
Clo-COOH	iv (bolus, s.d.)	0.1	24	test	0.90	0.80	1.13	-	-	-	Cushing 2012 [13]
Clo-COOH	iv (bolus, s.d.)	1	24	training	86.99	96.40	0.90	-	-	-	Cushing 2012 [13]
Clo-COOH	iv (bolus, s.d.)	10	24	training	998.68	1419.97	0.70	-	-	-	Cushing 2012 [13]
Clo-COOH	iv (bolus, s.d.)	30	24	test	2882.92	4021.05	0.72	-	-	-	Cushing 2012 [13]
Clo-COOH	iv (inf, 4 min, s.d.)	100	24	training	9227.09	13261.50	0.70	2084.94	4833.6	0.43	Cushing 2012 [13]
Clo-COOH	iv (inf, 8 min, s.d.)	300	24	training	27186.60	45293.51	0.60	6010.82	13714	0.44	Cushing 2012 [13]
Clo-COOH	po (tab, s.d.)	75	6	training	9676.82	9628.72	1.00	2649.93	2495.5	1.06	Savu 2016 [14]
Clo-COOH	po (-, s.d.)	75	10	training	11401.62	9955.33	1.15	2800.56	2978	0.94	Silvestro 2013 [15]
Clo-COOH	po (tab, s.d.)	75	20	test	15133.77	8022.51	1.89	3113.45	3172.96	0.98	Zou 2012 [17]
Clo-COOH	po (-, s.d.)	75	10	test	11401.80	13204.02	0.86	2800.48	3708	0.76	Silvestro 2011 [30]
Clo-COOH	po (tab, s.d.)	75	42	test	12285.91	13277.68	0.93	2992.86	3411.88	0.88	Junior 2010 [31]
Clo-COOH	po (tab, s.d.)	75	76	test	13654.84	7761.20	1.76	2592.13	2793.2	0.93	Yousef 2013 [32]
Clo-COOH	po (tab, s.d.)	75	12	test	10695.22	12636.01	0.85	2637.74	3522.86	0.75	Souri 2006 [33]
Clo-COOH	po (tab, s.d.)	75	32	test	11401.88	8027.59	1.42	2800.54	2313.16	1.21	Ksycinska 2006 [34]
Clo-COOH	po (tab, s.d.)	75	92	test	11394.77	6490.65	1.76	2655.54	2353.38	1.13	Brvar 2014 [19]
Clo-COOH	po (tab, s.d.)	150	24	training	29536.93	22093.33	1.34	5622.88	5852.1	0.96	Bahrami 2008 [35]
Clo-COOH	po (tab, s.d.)	300	24	training	40760.28	26893.08	1.52	10998.85	8534	1.29	Small 2008 [36]
Clo-COOH	po (-, l.d./m.d., 5d)	300/75	24	training	11441.36	7943.94	1.44 (D5)	2598.70	2511.04	1.03 (D5)	Härtter 2013 [25]
Clo-COOH	po (-, l.d./m.d., 5d)	300/75	40	test	63226.49	50100.02	1.26 (D1)	12982.35	15863	0.82 (D1)	Kim 2012 [27]
Clo-COOH	po (-, l.d./m.d., 6d)	300/75	44	test	26601.46	22289.85	1.19 (D1)	12872.45	11306.5	1.14 (D1)	Kim 2009 [28]
Clo-COOH	po (-, s.d.)	600	12	training	94049.45	81518.43	1.15	17387.22	19333.5	0.90	Härtter 2013 [25]
Clo-AG	po (tab, s.d.)	75	6	training	1569.80	1646.22	0.95	362.58	439.8	0.82	Savu 2016 [14]
Clo-AG	po (-, s.d.)	75	10	training	1780.04	1702.59	1.05	366.71	414	0.89	Silvestro 2013 [15]
Clo-AG	po (-, s.d.)	75	10	test	1780.40	1540.48	1.16	366.74	390.4	0.94	Silvestro 2011 [30]

-: not given, 2-Oxo-Clo: 2-Oxo-clopidogrel, AUC_{last}: area under the plasma concentration-time curve determined between first and last concentration measurements, Clo: clopidogrel, Clo-AG: clopidogrel acyl glucuronide, Clo-AM: clopidogrel thiol H4, Clo-COOH: clopidogrel carboxylic acid, C_{max}: maximum plasma concentration, d: dosage period in days, D: day of pharmacokinetic sampling, inf: infusion, GMFE: geometric mean fold error, iv: intravenous, l.d.: loading dose, m.d.: maintenance dose (once daily), n: number of participants, NM: cytochrome P450 2C19 normal metabolizer, obs: observed, PM: cytochrome P450 2C19 poor metabolizer, po: peroral, pred: predicted, s.d.: single dose, tab: tablet.

Table S6: Predicted versus observed AUC_{last} and C_{max} values, along with GMFE values *(continued)*

Compound	Clopidogrel dosing regimen		n	Dataset	AUC_{last}			C_{max}			Reference
	Route	Dose [mg]			Pred [$\frac{ng*h}{ml}$]	Obs [$\frac{ng*h}{ml}$]	Pred/Obs	Pred [$\frac{ng}{ml}$]	Obs [$\frac{ng}{ml}$]	Pred/Obs	
2-Oxo-Clo	po (-, s.d.)	75	5	training	43.55	43.83	0.99	18.07	18.94	0.95	Silvestro 2013 [15]
Clo-AM	po (-, s.d.)	75	5	test	17.94	33.91	0.53	11.77	27.86	0.42	Silvestro 2013 [15]
Clo-AM	po (-, m.d., 10d)	75	9	test	15.56	9.17	1.70 (D1)	12.75	9.62	1.33 (D1)	Li 2018 [37]
Clo-AM	po (-, m.d., 10d)	75	9	test	15.88	6.24	2.54 (D10)	11.88	5.99	1.98 (D10)	Li 2018 [37]
Clo-AM	po (tab, s.d.)	300	27	training	43.51	33.31	1.31	31.37	24.00	1.31	Kim 2016 [23]
Clo-AM	po (tab, s.d.)	300	20	test	45.02	50.99	0.88	30.87	37.57	0.82	Zhou 2018 [24]
Clo-AM	po (tab, s.d.)	300	36	test	34.53	53.40	0.65	28.87	41.35	0.70	Umemura 2016 [38]
Clo-AM	po (tab, s.d.)	300	66	test	38.94	47.93	0.81	27.77	33.49	0.83	Takahashi 2008 [39]
Clo-AM	po (tab, s.d.)	300	16	test	35.87	35.41	1.01	30.08	30.13	1.00	Small 2010 [40]
Clo-AM	po (-, l.d./m.d., 7d)	300/75	9 NM	training	40.99	40.41	1.01 (D1)	28.88	27.80	1.04 (D1)	Kobayashi 2015 [41]
Clo-AM	po (-, l.d./m.d., 7d)	300/75	9 NM	training	15.95	10.97	1.45 (D7)	11.66	9.75	1.20 (D7)	Kobayashi 2015 [41]
Clo-AM	po (-, l.d./m.d., 7d)	300/75	9 PM	training	11.75	15.37	0.76 (D1)	9.76	10.23	0.95 (D1)	Kobayashi 2015 [41]
Clo-AM	po (-, l.d./m.d., 7d)	300/75	9 PM	training	2.62	3.66	0.72 (D7)	2.57	3.52	0.73 (D7)	Kobayashi 2015 [41]
Clo-AM	po (-, l.d./m.d., 5d)	300/75	65	training	36.92	46.44	0.79 (D1)	27.07	33.95	0.80 (D1)	Angiolillo 2011 [42]
Clo-AM	po (-, l.d./m.d., 5d)	300/75	65	training	13.24	13.64	0.97 (D5)	10.94	14.49	0.75 (D5)	Angiolillo 2011 [42]
Clo-AM	po (-, l.d./m.d., 5d)	300/75	66	test	35.77	42.40	0.84 (D1)	26.33	31.63	0.83 (D1)	Hurbin 2012 [43]
Clo-AM	po (-, l.d./m.d., 5d)	300/75	66	test	12.93	13.12	0.99 (D5)	10.67	14.78	0.72 (D5)	Hurbin 2012 [43]
Clo-AM	po (-, l.d./m.d., 5d)	300/75	65	test	16.02	12.35	1.30 (D5)	10.85	11.41	0.95 (D5)	Furlong 2013 [44]
Clo-AM	po (tab, s.d.)	600	14	training	55.01	85.13	0.65	35.30	50.54	0.70	Holmberg 2014 [29]
			Overall mean GMFE (range)				1.38 (1.00–3.68)			1.35 (1.00–2.37)	
			GMFE ≤ 2				63/66			55/58	

-: not given, 2-Oxo-Clo: 2-Oxo-clopidogrel, AUC_{last}: area under the plasma concentration-time curve determined between first and last concentration measurements, Clo: clopidogrel, Clo-AG: clopidogrel acyl glucuronide, Clo-AM: clopidogrel thiol H4, Clo-COOH: clopidogrel carboxylic acid, C_{max}: maximum plasma concentration, d: dosage period in days, D: day of pharmacokinetic sampling, inf: infusion, GMFE: geometric mean fold error, iv: intravenous, l.d.: loading dose, m.d.: maintenance dose (once daily), n: number of participants, NM: cytochrome P450 2C19 normal metabolizer, obs: observed, PM: cytochrome P450 2C19 poor metabolizer, po: peroral, pred: predicted, s.d.: single dose, tab: tablet.

S2.7 Local Sensitivity Analysis

S2.7.1 Method

Local sensitivity of the final model to single parameter changes was calculated according to Equation S1 as ratio between the relative change of simulated area under the plasma concentration-time curve determined between first and last concentration measurements (AUC_{last}) and the relative variation of the respective parameter value used in the model. A relative perturbation of 1000% was applied (variation range 10.0, maximum number of 9 steps) and parameters included were either optimized or assumed to affect AUC_{last}.

$$S = \frac{\Delta AUC_{last}}{\Delta p} \cdot \frac{p}{AUC_{last}} \tag{S1}$$

where S = sensitivity of AUC_{last} to the examined model parameter, ΔAUC_{last} = change of AUC_{last}, Δp = change of the examined parameter value, p = original parameter value, and AUC_{last} = simulated AUC_{last} with the original parameter value.

The threshold for sensitivity was set at 0.5, which corresponds to a 50% change in simulated AUC_{last} given a 100% change in the parameter value examined.

S2.7.2 Results

Local sensitivity analysis of a multiple dose simulation over ten days involving administration of 75 mg clopidogrel daily was conducted. Table S7 lists all parameters evaluated for their influence on AUC_{last} of clopidogrel or its metabolites. Separate results are shown in Figures S23, S24, S25, S26, and S27 for clopidogrel and each metabolite, respectively.

Table S7: Parameters evaluated during the local sensitivity analysis of clopidogrel and its metabolites

Compound	Parameter	Source	Compound	Parameter	Source
Clo	CES1 k_{cat}	Optimized	Clo-AG	CL_{ren}	Optimized
Clo	CES1 K_M	Literature	Clo-AG	CYP3A4 K_i CI	Literature
Clo	CES2 k_{cat}	Optimized	Clo-AG	f_u	Literature
Clo	CES2 K_M	Literature	Clo-AG	lipophilicity	Optimized
Clo	CYP2C19 NM k_{cat}	Optimized	Clo-AG	pKa, acid	Literature
Clo	CYP2C19 K_i CI	Literature	Clo-AG	pKa, base	Literature
Clo	CYP2C19 K_i MBI	Literature	Clo-AG	Solubility	Literature
Clo	CYP2C19 K_I MBI	Literature	Clo-AG	Intestinal permeability	Calculated
Clo	CYP2C19 k_{inact} MBI	Literature	2-Oxo-Clo	CL_{hep}	Optimized
Clo	CYP2C19 K_M	Literature	2-Oxo-Clo	CYP2C19 NM k_{cat}	Optimized
Clo	CYP3A4 k_{cat}	Optimized	2-Oxo-Clo	CYP2C19 K_i CI	Literature
Clo	CYP3A4 K_i CI	Literature	2-Oxo-Clo	CYP2C19 K_M	Literature
Clo	CYP3A4 K_i MBI	Literature	2-Oxo-Clo	CYP3A4 k_{cat}	Optimized
Clo	CYP3A4 K_I MBI	Literature	2-Oxo-Clo	CYP3A4 K_i CI	Literature
Clo	CYP3A4 k_{inact} MBI	Literature	2-Oxo-Clo	CYP3A4 K_M	Literature
Clo	CYP3A4 K_M	Literature	2-Oxo-Clo	f_u	Literature
Clo	Dissolution shape	Optimized	2-Oxo-Clo	GFR fraction	Assumed
Clo	Dissolution time	Optimized	2-Oxo-Clo	lipophilicity	Optimized
Clo	f_u	Literature	2-Oxo-Clo	pKa, acid	Literature
Clo	GFR fraction	Assumed	2-Oxo-Clo	pKa, base	Literature
Clo	lipophilicity	Optimized	2-Oxo-Clo	Solubility	Literature
Clo	pKa, base	Literature	2-Oxo-Clo	Intestinal permeability	Calculated
Clo	Solubility	Literature	Clo-AM	CL_{hep}	Optimized
Clo	Intestinal permeability	Optimized	Clo-AM	f_u	Optimized
Clo-COOH	CYP3A4 K_i CI	Literature	Clo-AM	GFR fraction	Assumed
Clo-COOH	f_u	Optimized	Clo-AM	lipophilicity	Optimized
Clo-COOH	GFR fraction	Assumed	Clo-AM	pKa1, acid	Literature
Clo-COOH	lipophilicity	Optimized	Clo-AM	pKa2, acid	Literature
Clo-COOH	pKa, acid	Literature	Clo-AM	pKa, base	Literature
Clo-COOH	pKa, base	Literature	Clo-AM	Solubility	Literature
Clo-COOH	Solubility	Literature	Clo-AM	Intestinal permeability	Calculated
Clo-COOH	Intestinal permeability	Calculated			
Clo-COOH	UGT2B7 k_{cat}	Optimized			
Clo-COOH	UGT2B7 K_M	Literature			

2-Oxo-Clo: 2-Oxo-clopidogrel, CES: carboxylesterase, CI: competitive inhibition, Clo: clopidogrel, CL_{hep}: unspecific hepatic clearance, Clo-AG: clopidogrel acyl glucuronide, Clo-AM: clopidogrel thiol H4, Clo-COOH: clopidogrel carboxylic acid, CL_{ren}: unspecific renal clearance, CYP: cytochrome P450, f_u: fraction unbound, GFR: glomerular filtration rate, k_{cat}: catalytic rate constant, K_i: concentration for half-maximal inhibition, K_I: concentration for half-maximal inactivation, k_{inact}: maximum inactivation rate constant, K_M: Michaelis-Menten constant, MBI: mechanism-based inactivation, NM: normal metabolizer, pKa: acid dissociation constant, UGT: uridine 5'-diphospho-glucuronosyltransferase.

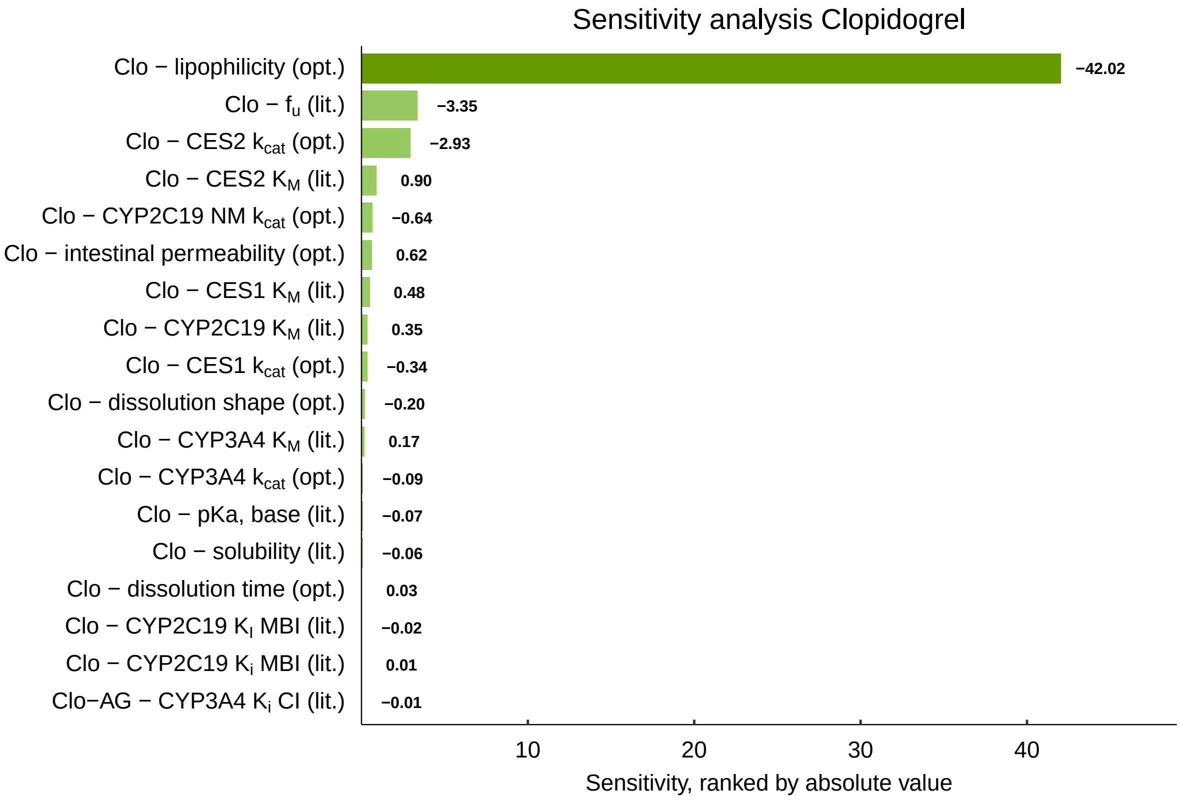

Figure S23: Clopidogrel model sensitivity analysis - clopidogrel. CES: carboxylesterase, CI: competitive inhibition, Clo: clopidogrel, Clo-AG: clopidogrel acyl glucuronide, CYP: cytochrome P450, f_u: fraction unbound, k_{cat}: catalytic rate constant, K_i: concentration for half-maximal inhibition, K_I: concentration for half-maximal inactivation, k_{inact}: maximum inactivation rate constant, K_M: Michaelis-Menten constant, lit.: literature, MBI: mechanism-based inactivation, NM: cytochrome P450 2C19 normal metabolizer, opt.: optimized, pKa: acid dissociation constant.

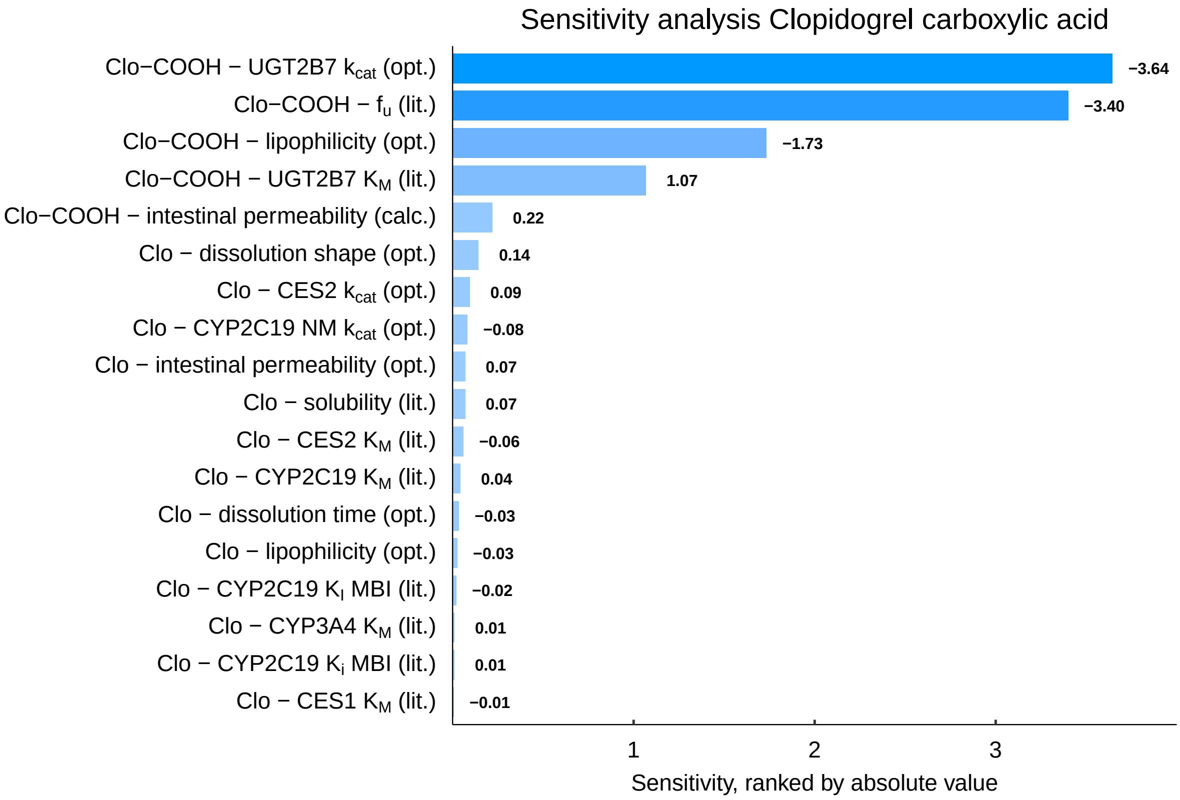

Figure S24: Clopidogrel model sensitivity analysis - clopidogrel carboxylic acid. Calc.: calculated, CES: carboxylesterase, Clo: clopidogrel, Clo-COOH: clopidogrel carboxylic acid, CYP: cytochrome P450, f_u: fraction unbound, k_{cat}: catalytic rate constant, K_I: concentration for half-maximal inactivation, k_{inact}: maximum inactivation rate constant, K_M: Michaelis-Menten constant, lit.: literature, MBI: mechanism-based inactivation, NM: cytochrome P450 2C19 normal metabolizer, opt.: optimized, UGT: uridine 5'-diphospho-glucuronosyltransferase.

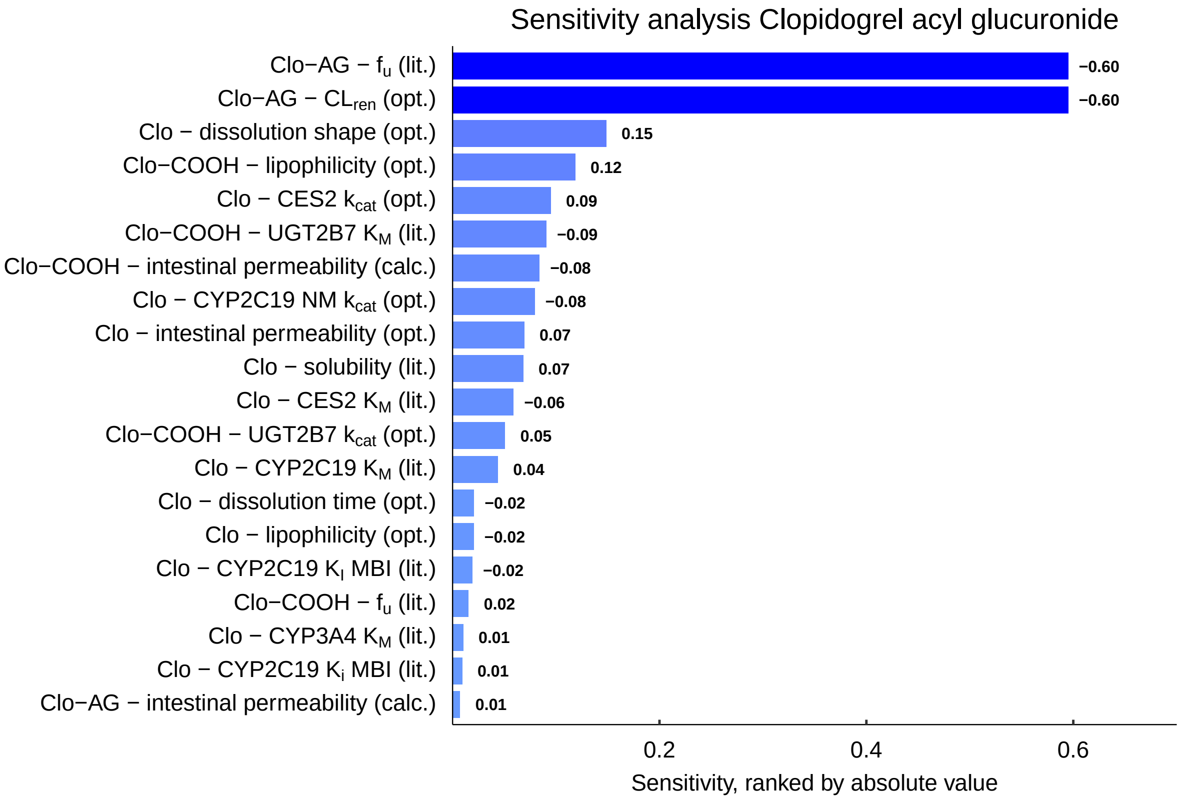

Figure S25: Clopidogrel model sensitivity analysis - clopidogrel acyl glucuronide. Calc.: calculated, CES: carboxylesterase, Clo: clopidogrel, Clo-AG: clopidogrel acyl glucuronide, Clo-COOH: clopidogrel carboxylic acid, CL_{ren}: unspecific renal clearance, CYP: cytochrome P450, f_u: fraction unbound, k_{cat}: catalytic rate constant, K_I: concentration for half-maximal inactivation, k_{inact}: maximum inactivation rate constant, K_M: Michaelis-Menten constant, lit.: literature, MBI: mechanism-based inactivation, NM: cytochrome P450 2C19 normal metabolizer, opt.: optimized, UGT: uridine 5'-diphospho-glucuronosyltransferase.

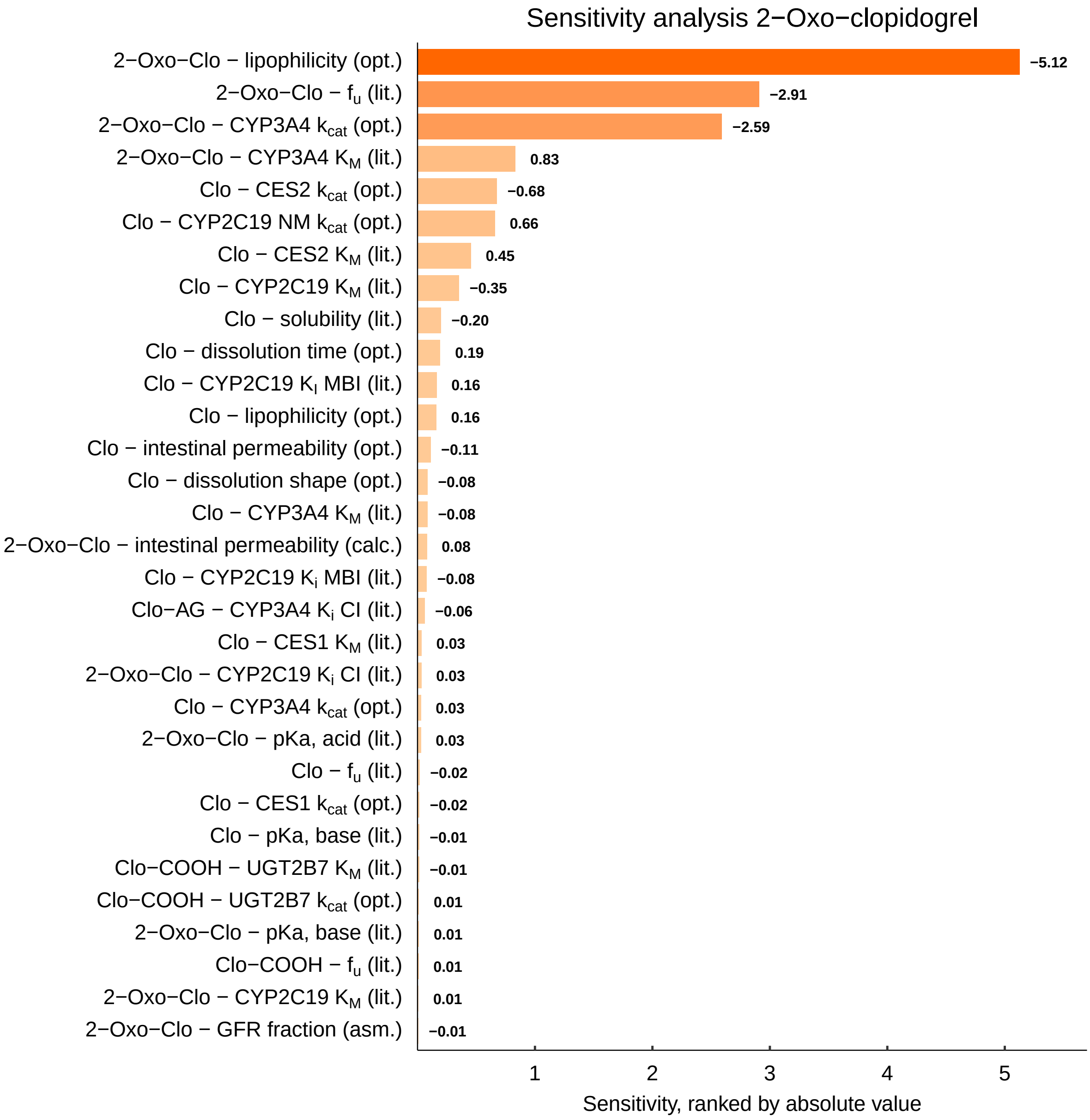

Figure S26: Clopidogrel model sensitivity analysis - 2-oxo-clopidogrel. 2-Oxo-Clo: 2-Oxo-clopidogrel, asm.: assumed, calc.: calculated, CES: carboxylesterase, CI: competitive inhibition, Clo: clopidogrel, Clo-AG: clopidogrel acyl glucuronide, Clo-COOH: clopidogrel carboxylic acid, CYP: cytochrome P450, f_u: fraction unbound, GFR: glomerular filtration rate, k_{cat}: catalytic rate constant, K_i: concentration for half-maximal inhibition, K_I: concentration for half-maximal inactivation, k_{inact}: maximum inactivation rate constant, K_M: Michaelis-Menten constant, lit.: literature, MBI: mechanism-based inactivation, NM: cytochrome P450 2C19 normal metabolizer, pKa: acid dissociation constant, opt.: optimized, UGT: uridine 5'-diphospho-glucuronosyltransferase.

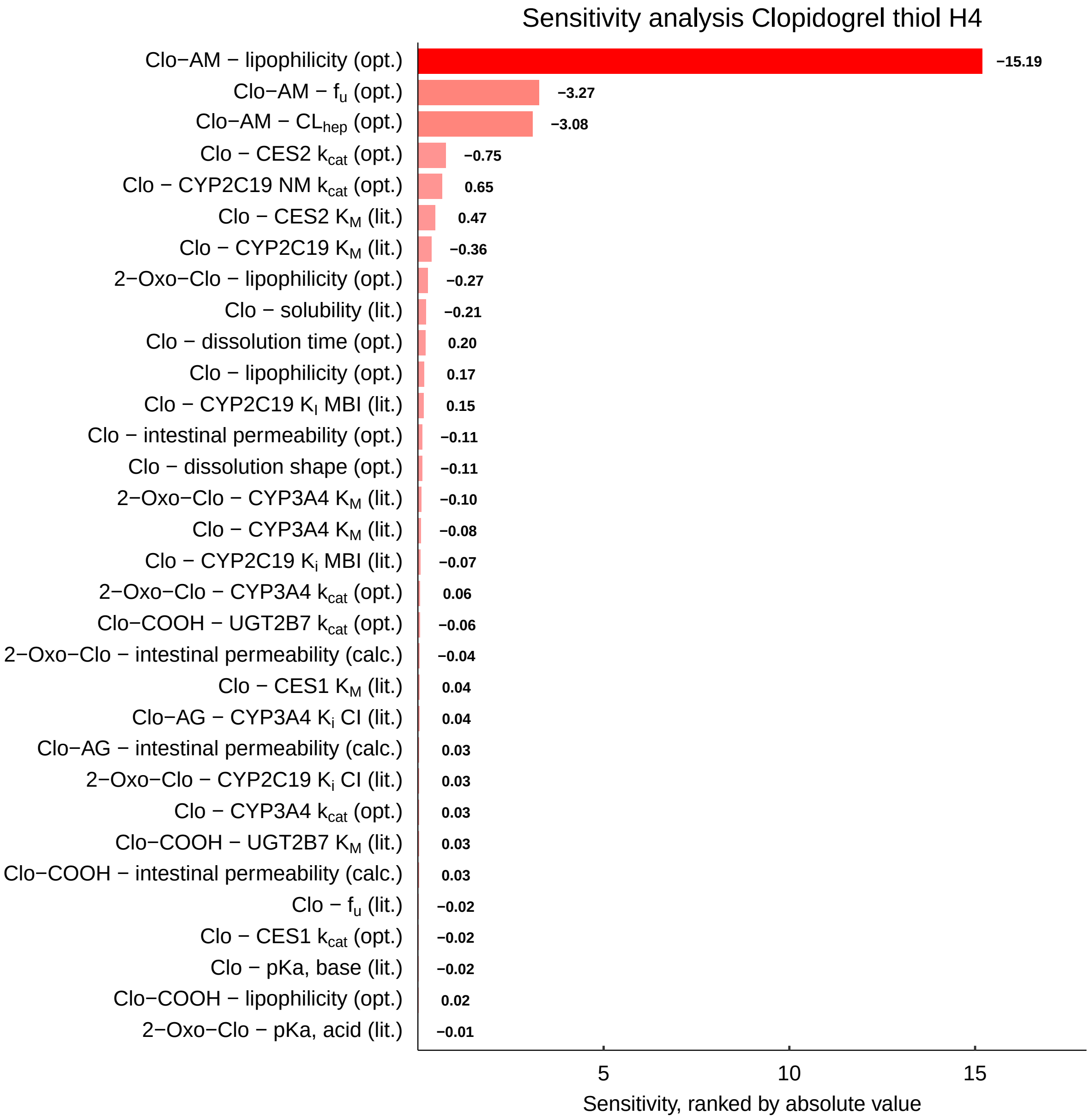

Figure S27: Clopidogrel model sensitivity analysis - clopidogrel thiol H4. 2-Oxo-Clo: 2-Oxo-clopidogrel, calc.: calculated, CES: carboxylesterase, CI: competitive inhibition, Clo: clopidogrel, CL_{hep}: unspecific hepatic clearance, Clo-AG: clopidogrel acyl glucuronide, Clo-AM: clopidogrel thiol H4, Clo-COOH: clopidogrel carboxylic acid, CYP: cytochrome P450, f_u: fraction unbound, k_{cat}: catalytic rate constant, K_i: concentration for half-maximal inhibition, K_I: concentration for half-maximal inactivation, k_{inact}: maximum inactivation rate constant, K_M: Michaelis-Menten constant, lit.: literature, MBI: mechanism-based inactivation, NM: cytochrome P450 2C19 normal metabolizer, pKa: acid dissociation constant, opt.: optimized, UGT: uridine 5'-diphospho-glucuronosyltransferase.

S3 DGI Modeling

S3.1 Clinical Study Data

Table S8: Clopidogrel DGI model study table

Compound measured	Clopidogrel dosing regimen: Route	Clopidogrel dosing regimen: Dose [mg]	n	Females [%]	Ethnicity implemented	Age [years]	Weight [kg]	Height [cm]	Reference
Clo	po (tab, s.d.)	300	8 NM	-	Asian	22.3±1.6	64.8±4.7	172.9±3.3	Song 2018 [79]
Clo	po (tab, s.d.)	300	10 IM	-	Asian	21.6±1.1	65.0±5.1	173.6±3.4	Song 2018 [79]
Clo	po (tab, s.d.)	300	2 PM	-	Asian	20.0±0.0	58.5±0.7	169.0±1.4	Song 2018 [79]
Clo	po (-, l.d./m.d., 7d)	300/75	8 NM	0	Asian	23.5±2.9	66.6±5.6	-	Kim 2008 [26]
Clo	po (-, l.d./m.d., 7d)	300/75	8 IM	0	Asian	24.3±1.7	73.0±6.3	-	Kim 2008 [26]
Clo	po (-, l.d./m.d., 7d)	300/75	8 PM	0	Asian	24.1±2.8	67.3±5.6	-	Kim 2008 [26]
Clo-AM	po (-, m.d., 10d)	75	5 NM	33	Asian	30.3±7.5	63.8±8.5	165.4±7.2	Li 2018 [37]
Clo-AM	po (-, m.d., 10d)	75	4 IM	33	Asian	30.3±7.5	63.8±8.5	165.4±7.2	Li 2018 [37]
Clo-AM	po (tab, s.d.)	300	8 NM	-	Asian	22.3±1.6	64.8±4.7	172.9±3.3	Song 2018 [79]
Clo-AM	po (tab, s.d.)	300	10 IM	-	Asian	21.6±1.1	65.0±5.1	173.6±3.4	Song 2018 [79]
Clo-AM	po (tab, s.d.)	300	2 PM	-	Asian	20.0±0.0	58.5±0.7	169.0±1.4	Song 2018 [79]
Clo-AM	po (-, l.d./m.d., 7d)	300/75	16 NM	56	Asian	36.7±5.6<br>(24–43)	64.8±8.4<br>(51.3–79.8)	-	Zhang 2020 [80]
Clo-AM	po (-, l.d./m.d., 7d)	300/75	16 IM	50	Asian	33.7±6.8<br>(19–42)	62.8±6.5<br>(51.4–78.6)	-	Zhang 2020 [80]
Clo-AM	po (-, l.d./m.d., 7d)	300/75	16 PM	44	Asian	35.0±4.8<br>(29–44)	63.7±6.6<br>(49.6–76.8)	-	Zhang 2020 [80]

-: not given, Clo: clopidogrel, Clo-AM: clopidogrel thiol H4, d: dosage period in days, IM: cytochrome P450 2C19 intermediate metabolizer, l.d.: loading dose, m.d.: maintenance dose (once daily), n: number of participants, NM: cytochrome P450 2C19 normal metabolizer, PM: cytochrome P450 2C19 poor metabolizer, po: peroral, s.d.: single dose, tab: tablet; values for age, weight and height are presented as mean ± standard deviation (range).

Table S8: Clopidogrel DGI model study table *(continued)*

Compound	Clopidogrel dosing regimen		n	Females	Ethnicity	Age	Weight	Height	Reference
measured	Route	Dose [mg]		[%]	implemented	[years]	[kg]	[cm]	
Clo-AM	po (-, l.d./m.d., 7d)	300/75	9 NM	0	Japanese	(20–35)	(50.4–87.4)	-	Kobayashi 2015 [41]
Clo-AM	po (-, l.d./m.d., 7d)	300/75	9 IM	0	Japanese	(20–35)	(50.4–87.4)	-	Kobayashi 2015 [41]
Clo-AM	po (-, l.d./m.d., 7d)	300/75	9 PM	0	Japanese	(20–35)	(50.4–87.4)	-	Kobayashi 2015 [41]
Clo-AM	po (-, l.d./m.d., 7d)	600/150	9 NM	0	Japanese	(20–35)	(50.4–87.4)	-	Kobayashi 2015 [41]
Clo-AM	po (-, l.d./m.d., 7d)	600/150	9 IM	0	Japanese	(20–35)	(50.4–87.4)	-	Kobayashi 2015 [41]
Clo-AM	po (-, l.d./m.d., 7d)	600/150	9 PM	0	Japanese	(20–35)	(50.4–87.4)	-	Kobayashi 2015 [41]

-: not given, Clo: clopidogrel, Clo-AM: clopidogrel thiol H4, d: dosage period in days, IM: cytochrome P450 2C19 intermediate metabolizer, l.d.: loading dose, m.d.: maintenance dose (once daily), n: number of participants, NM: cytochrome P450 2C19 normal metabolizer, PM: cytochrome P450 2C19 poor metabolizer, po: peroral, s.d.: single dose, tab: tablet; values for age, weight and height are presented as mean ± standard deviation (range).

S3.2 Plasma Concentration-Time Profiles (Semilogarithmic)

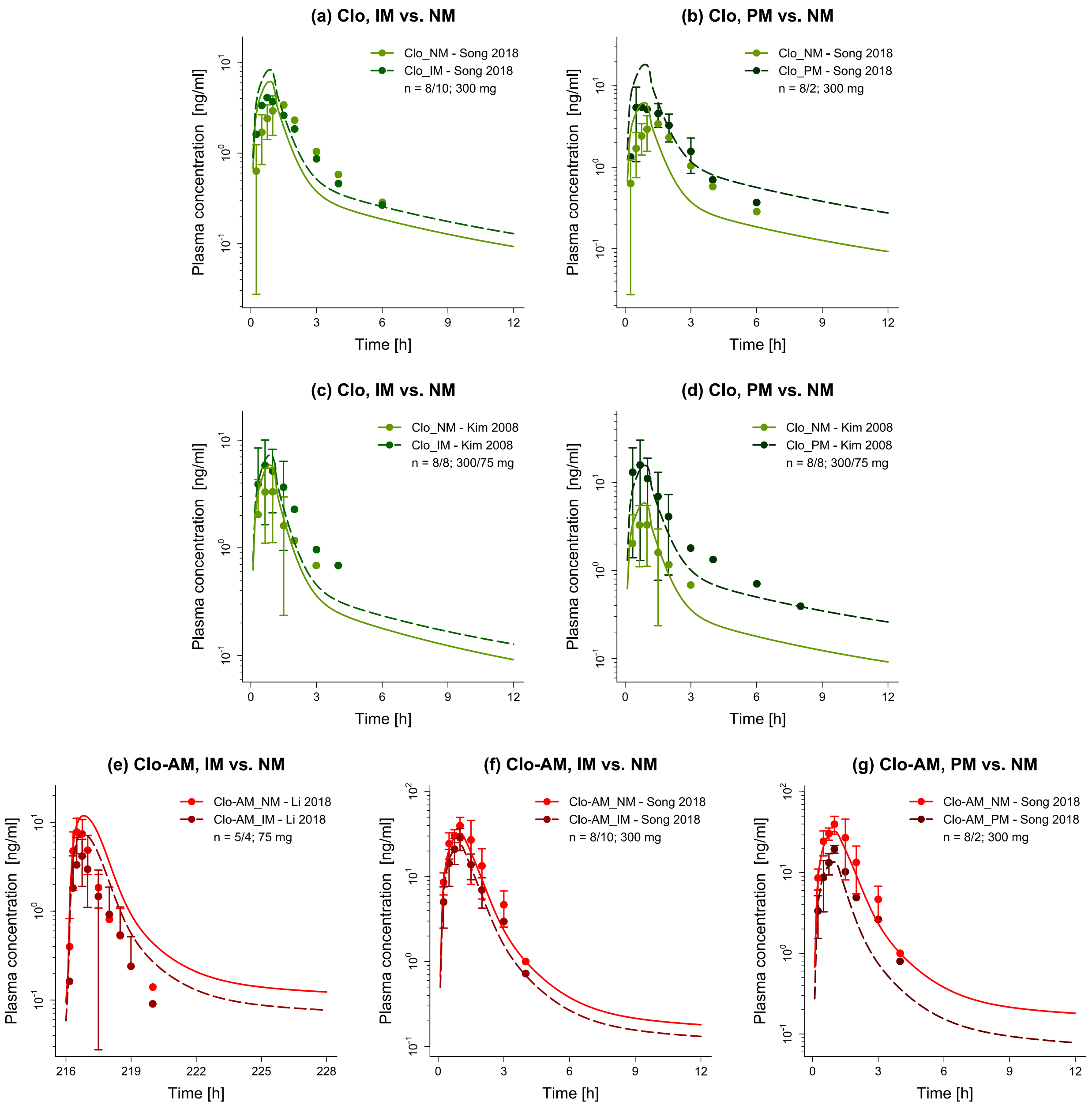

Figure S28: Drug–gene interaction model evaluation. Presented are predicted plasma concentration-time profiles (semilogarithmic plots) of (a–d) clopidogrel and (e–g) Clo-AM for IM and PM compared separately to NM phenotypes, alongside corresponding observed data [26, 37, 79]. Dashed (IM or PM) and solid (NM) lines represent the model predictions, while corresponding observed data are shown as symbols (± standard deviation, if available). Clo: clopidogrel, Clo-AM: clopidogrel thiol H4, IM: cytochrome P450 (CYP) 2C19 intermediate metabolizer, n: number of participants, NM: CYP2C19 normal metabolizer, PM: CYP2C19 poor metabolizer.

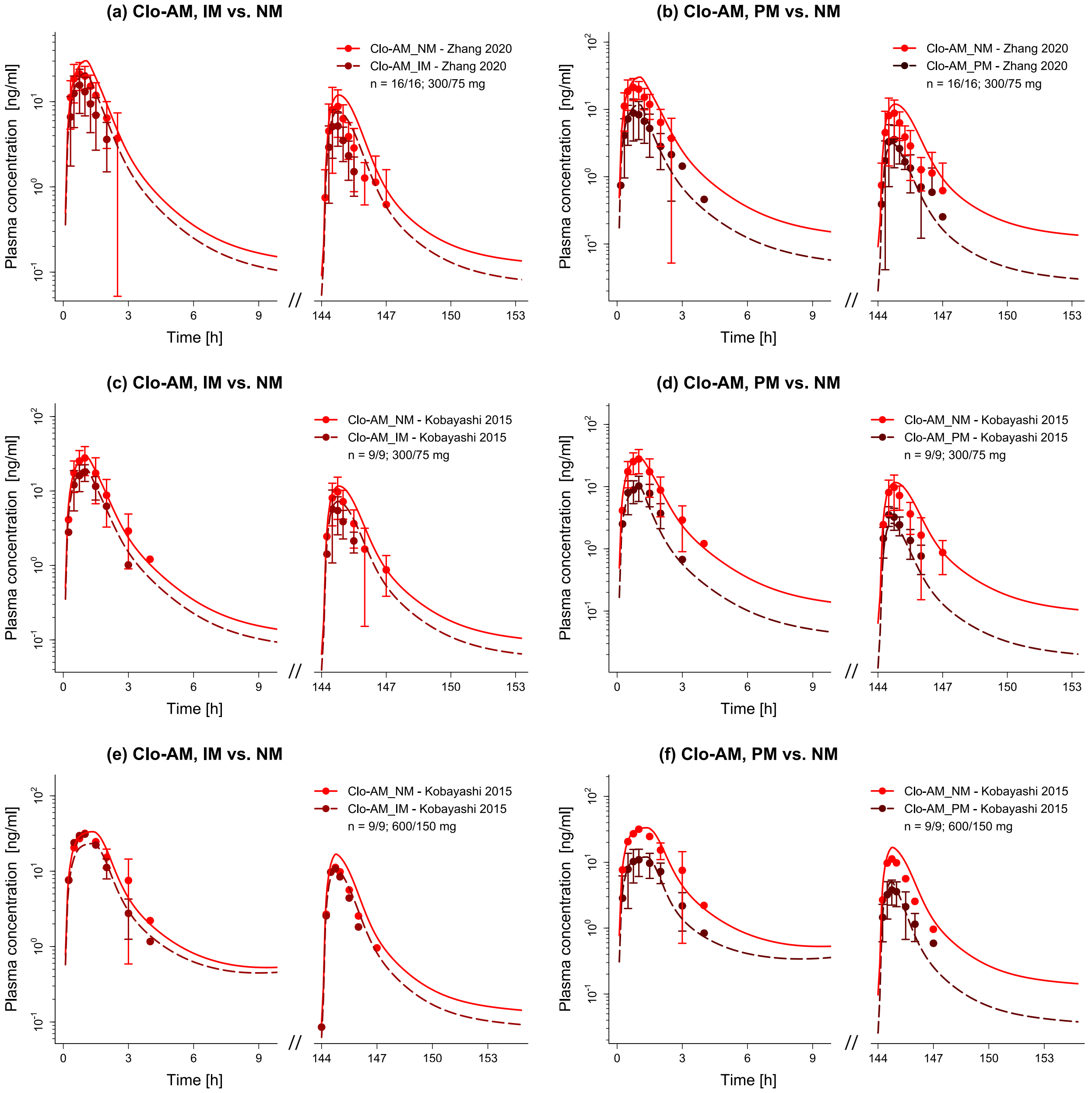

Figure S29: Drug–gene interaction model evaluation. Presented are predicted plasma concentration-time profiles (semilogarithmic plots) of Clo-AM for IM and PM compared separately to NM phenotypes, alongside corresponding observed data [41, 80]. Dashed (IM or PM) and solid (NM) lines represent the model predictions, while corresponding observed data are shown as symbols (± standard deviation, if available). Clo-AM: clopidogrel thiol H4, IM: cytochrome P450 (CYP) 2C19 intermediate metabolizer, n: number of participants, NM: CYP2C19 normal metabolizer, PM: CYP2C19 poor metabolizer.

S3.3 Plasma Concentration-Time Profiles (Linear)

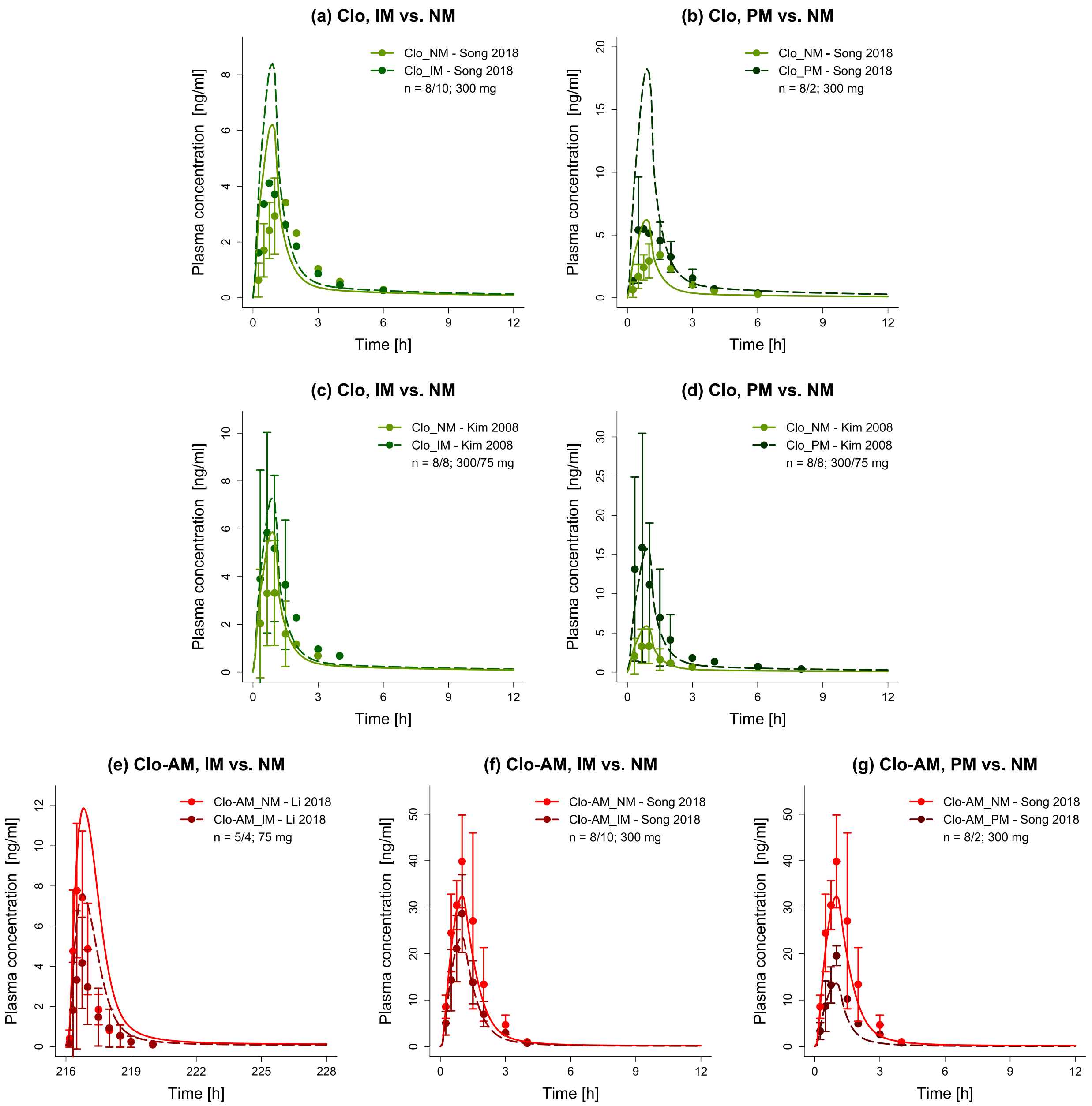

Figure S30: Drug–gene interaction model evaluation. Presented are predicted plasma concentration-time profiles (linear plots) of (a–d) clopidogrel and (e–g) Clo-AM for IM and PM compared separately to NM phenotypes, alongside corresponding observed data [26, 37, 79]. Dashed (IM or PM) and solid (NM) lines represent the model predictions, while corresponding observed data are shown as symbols (± standard deviation, if available). Clo: clopidogrel, Clo-AM: clopidogrel thiol H4, IM: cytochrome P450 (CYP) 2C19 intermediate metabolizer, n: number of participants, NM: CYP2C19 normal metabolizer, PM: CYP2C19 poor metabolizer.

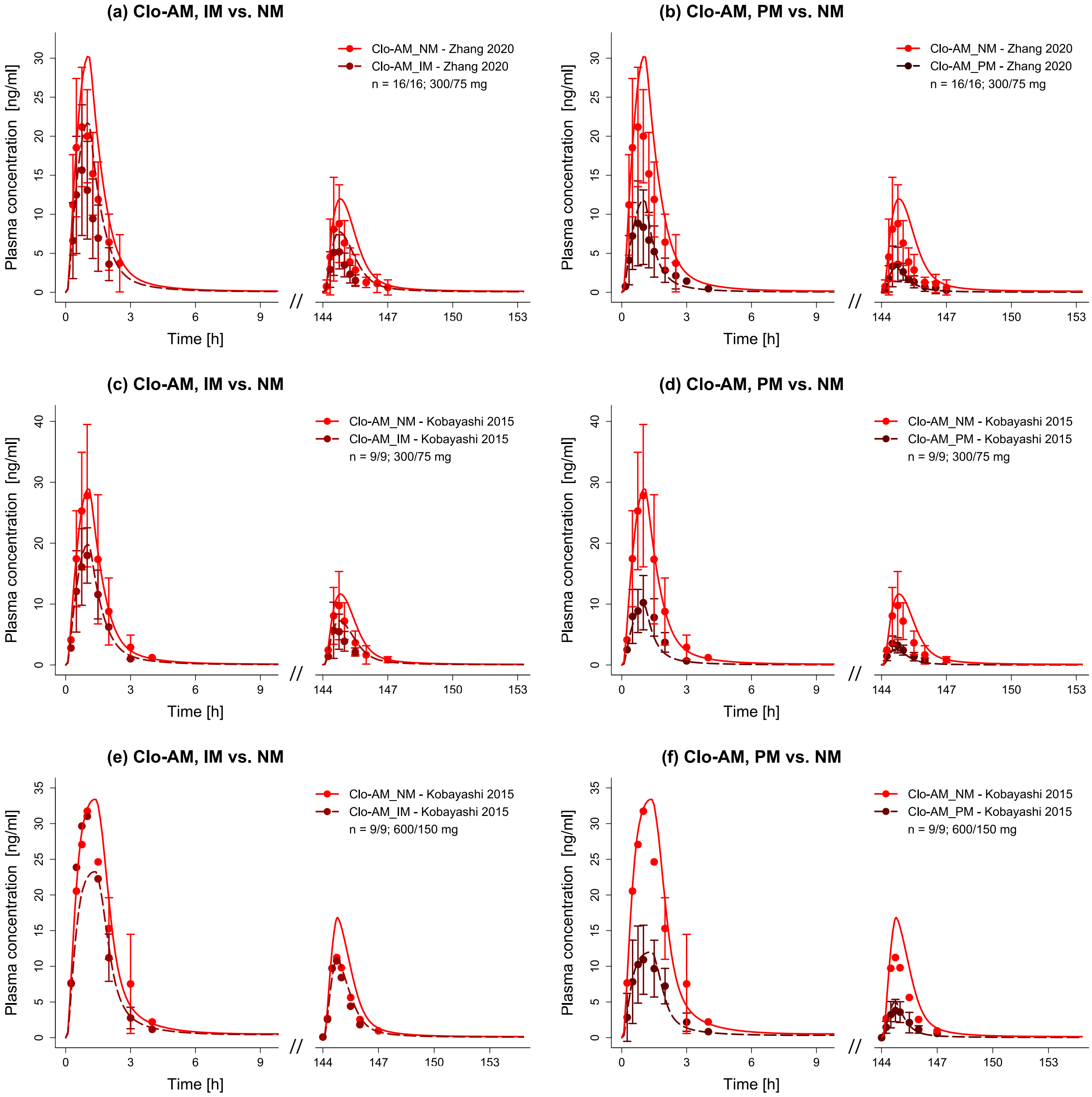

Figure S31: Drug–gene interaction model evaluation. Presented are predicted plasma concentration-time profiles (linear plots) of Clo-AM for IM and PM compared separately to NM phenotypes, alongside corresponding observed data [41, 80]. Dashed (IM or PM) and solid (NM) lines represent the model predictions, while corresponding observed data are shown as symbols (± standard deviation, if available). Clo-AM: clopidogrel thiol H4, IM: cytochrome P450 (CYP) 2C19 intermediate metabolizer, n: number of participants, NM: CYP2C19 normal metabolizer, PM: CYP2C19 poor metabolizer.

S3.4 DGI AUC_{last} and DGI C_{max} Ratio Goodness-of-Fit Plots

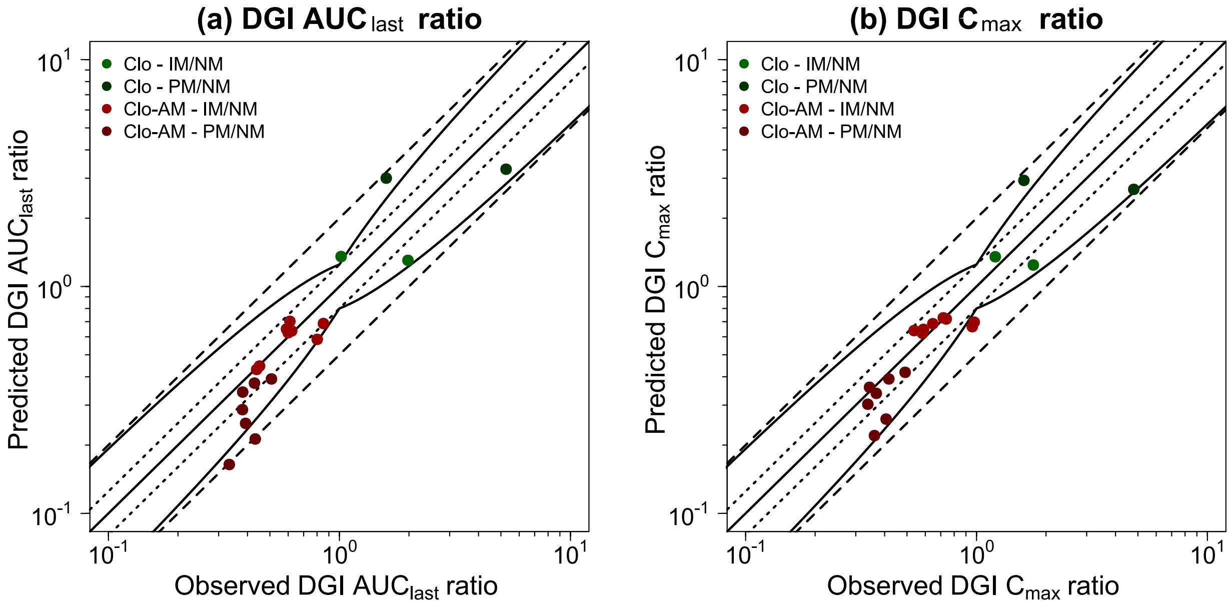

Figure S32: Drug–gene interaction model evaluation. Predicted versus observed (a) DGI AUC_{last} and (b) DGI C_{max} ratios are shown with the solid line representing the line of identity, dotted lines indicating 1.25-fold and dashed lines 2-fold deviation from the respective observed value [26, 37, 41, 79, 80], along with the curved lines marking the prediction success limits proposed by Guest et al. [81] (including 20% variability to account for uncertainties in observed ratios). AUC_{last}: area under the plasma concentration-time curve determined between first and last concentration measurements, Clo: clopidogrel, Clo-AM: clopidogrel thiol H4, C_{max}: maximum plasma concentration, DGI: drug–gene interaction, IM: cytochrome P450 (CYP) 2C19 intermediate metabolizer, n: number of participants, NM: CYP2C19 normal metabolizer, PM: CYP2C19 poor metabolizer.

S3.5 Predicted and Observed DGI AUC_{last} and DGI C_{max} Ratios with Mean GMFEs

Table S9: Predicted versus observed DGI AUC_{last} and DGI C_{max} ratios, along with GMFE values

Compound	Clopidogrel dosing regimen		n	DGI AUC_{last} ratio			DGI C_{max} ratio			Reference
	Route	Dose [mg]		Pred	Obs	Pred/Obs	Pred	Obs	Pred/Obs	
Clo	po (tab, s.d.)	300	10 IM/8 NM	1.36	1.02	1.33	1.35	1.21	1.12	Song 2018 [79]
Clo	po (tab, s.d.)	300	2 PM/8 NM	3.00	1.59	1.88	2.93	1.60	1.83	Song 2018 [79]
Clo	po (-, l.d./m.d., 7d)	300/75	8 IM/8 NM	1.30	1.98	0.66 (D1)	1.24	1.76	0.71 (D1)	Kim 2008 [26]
Clo	po (-, l.d./m.d., 7d)	300/75	8 PM/8 NM	3.29	5.26	0.62 (D1)	2.68	4.80	0.56 (D1)	Kim 2008 [26]
Clo-AM	po (-, m.d., 10d)	75	4 IM/5 NM	0.63	0.60	1.04 (D10)	0.64	0.54	1.19 (D10)	Li 2018 [37]
Clo-AM	po (tab, s.d.)	300	10 IM/8 NM	0.70	0.61	1.15	0.73	0.72	1.01	Song 2018 [79]
Clo-AM	po (tab, s.d.)	300	2 PM/8 NM	0.38	0.43	0.87	0.42	0.49	0.85	Song 2018 [79]
Clo-AM	po (-, l.d./m.d., 7d)	300/75	16 IM/16 NM	0.65	0.59	1.10 (D1)	0.72	0.74	0.97 (D1)	Zhang 2020 [80]
Clo-AM	po (-, l.d./m.d., 7d)	300/75	16 IM/16 NM	0.43	0.44	0.98 (D7)	0.65	0.59	1.10 (D7)	Zhang 2020 [80]
Clo-AM	po (-, l.d./m.d., 7d)	300/75	16 PM/16 NM	0.39	0.51	0.77 (D1)	0.39	0.42	0.94 (D1)	Zhang 2020 [80]
Clo-AM	po (-, l.d./m.d., 7d)	300/75	16 PM/16 NM	0.21	0.43	0.49 (D7)	0.26	0.41	0.64 (D7)	Zhang 2020 [80]
Clo-AM	po (-, l.d./m.d., 7d)	300/75	9 IM/9 NM	0.64	0.62	1.03 (D1)	0.69	0.65	1.06 (D1)	Kobayashi 2015 [41]
Clo-AM	po (-, l.d./m.d., 7d)	300/75	9 IM/9 NM	0.45	0.45	0.99 (D7)	0.62	0.58	1.07 (D7)	Kobayashi 2015 [41]
Clo-AM	po (-, l.d./m.d., 7d)	300/75	9 PM/9 NM	0.29	0.38	0.75 (D1)	0.34	0.37	0.92 (D1)	Kobayashi 2015 [41]
Clo-AM	po (-, l.d./m.d., 7d)	300/75	9 PM/9 NM	0.16	0.33	0.49 (D7)	0.22	0.36	0.61 (D7)	Kobayashi 2015 [41]
Clo-AM	po (-, l.d./m.d., 7d)	600/150	9 IM/9 NM	0.69	0.85	0.81 (D1)	0.70	0.98	0.71 (D1)	Kobayashi 2015 [41]
Clo-AM	po (-, l.d./m.d., 7d)	600/150	9 IM/9 NM	0.58	0.80	0.73 (D7)	0.67	0.96	0.69 (D7)	Kobayashi 2015 [41]
Clo-AM	po (-, l.d./m.d., 7d)	600/150	9 PM/9 NM	0.34	0.38	0.90 (D1)	0.36	0.34	1.05 (D1)	Kobayashi 2015 [41]
Clo-AM	po (-, l.d./m.d., 7d)	600/150	9 PM/9 NM	0.25	0.39	0.64 (D7)	0.30	0.34	0.89 (D7)	Kobayashi 2015 [41]
			Overall mean GMFE (range)			1.36 (1.01–2.03)			1.27 (1.01–1.83)	
			GMFE ≤ 2			17/19			19/19	

-: not given, AUC_{last}: area under the plasma concentration-time curve determined between first and last concentration measurements, Clo: clopidogrel, Clo-AM: clopidogrel thiol H4, d: dosage period in days, C_{max}: maximum plasma concentration, D: day of pharmacokinetic sampling, IM: cytochrome P450 2C19 intermediate metabolizer, l.d.: loading dose, m.d.: maintenance dose (once daily), n: number of participants, NM: cytochrome P450 2C19 normal metabolizer, obs: observed, PM: cytochrome P450 2C19 poor metabolizer, po: peroral, pred: predicted, s.d.: single dose, tab: tablet.

S4 DDI Network Modeling

S4.1 Types of Interaction Implemented

S4.1.1 Competitive Inhibition

Competitive inhibition (CI) involves reversible binding of an inhibitor to the respective enzyme or transporter, thus competing with substrates for the binding site, increasing the apparent Michaelis-Menten constant ($K_{M,app}$) while leaving the maximum reaction velocity (v_{max}) unaltered (Equations S2 and S3) [55]. Due to its reversibility, the inhibition can be overcome through an increase in substrate concentration (concentration-dependent).

$$v = \frac{v_{max} \cdot [S]}{K_{M,app} + [S]} \tag{S2}$$

$$K_{M,app} = K_M \cdot (1 + \frac{[I]}{K_i}) \tag{S3}$$

where v = reaction velocity, v_{max} = maximum reaction velocity, $[S]$ = free substrate concentration, $K_{M,app}$ = apparent Michaelis-Menten constant (inhibitor present), K_M = Michaelis-Menten constant (inhibitor absent), $[I]$ = free inhibitor concentration, and K_i = dissociation constant of the inhibitor-enzyme/ -transporter complex.

S4.1.2 Mechanism-Based Inactivation

In the case of mechanism-based inactivation (MBI), the inactivator, in addition to reversibly binding to the enzyme or transporter in question, is irreversibly converted into a reactive species that forms a covalent complex with the respective target (time-dependent inhibition). Due to the irreversibility, MBI can only be reversed by *de novo* synthesis of the relevant enzyme or transporter. In PK-Sim®, MBI and its resulting impact on enzyme turnover is implemented according to Equation S4 [55].

$$\frac{d[T]}{dt} = k_{deg} \cdot [T]_0 - (k_{deg} + \frac{k_{inact} \cdot [I]}{K_I + [I]}) \cdot [T] \tag{S4}$$

where $d[T]/dt$ = enzyme or transporter turnover, k_{deg} = degradation rate constant, $[T]_0$ = initial enzyme or transporter concentration at time 0, k_{inact} = maximum inactivation rate constant, K_I = concentration for half-maximal inactivation, $[I]$ = free inactivator concentration, and $[T]$ = enzyme or transporter concentration.

S4.1.3 Induction

Induction of enzymes or transporters is usually caused by activation of specific nuclear receptors through binding of an inducer, resulting in increased *de novo* synthesis of the enzyme or transporter of interest. Equation S5 describes the correlation between maximum induction effect (E_{max}), concentration for half-maximal induction (EC_{50}) and the magnitude of induction, with the first two parameters used for implementation of an induction process in PK-Sim® [55, 82].

$$E = \frac{E_{max} \cdot [Ind]}{EC_{50} + [Ind]} \tag{S5}$$

where E = magnitude of induction, E_{max} = maximum induction effect, $[Ind]$ = free inducer concentration (steady-state), and EC_{50} = concentration for half-maximal induction.

S4.2 Clinical Study Data

Table S10: Clopidogrel DDI network study table

Perpe-trator	Perpetrator application [mg]	Victim	Victim application [mg]	Compound measured	n	F [%]	Ethnicity implemented	Age [years]	Weight [kg]	Height [cm]	Dataset	Reference
Omep	d1–29: 80<br>po (cap, m.d.)	Clo	d1: 300<br>d2–29: 75<br>po (tab, l.d./m.d.)	Clo-AM	81	42	White American	30±9	73.9±11.8	171.5±9.8	test	Andersson 2014 [83]
Omep	d1–7: 20<br>po (-, m.d.)	Clo	d1–7: 75<br>po (-, m.d.)	Clo-AM	36	0	European	33.6±7.9	74.1±8.7	-	test	Funck-Brentano 2013 [84]
Rifa	d1–15: 300<br>po (-, b.i.d.)	Clo	d8: 600<br>d9–15: 75<br>po (-, l.d./m.d.)	Clo-AM	12	0	European	24<br>(19–42)	-	-	test	Judge 2010 [85]
Clo	d1–4: 75<br>po (tab, m.d.)	Bupro	d4+1h: 150<br>po (tab SR, s.d.)	(OH-)Bupro	12	0	European	(22–27)	(67–95)	-	test	Turpeinen 2005 [86]
Clo	d1: 300<br>d2: 75<br>po (tab, l.d./m.d.)	Monte	d1+1h: 10<br>po (tab, s.d.)	Monte	12	58	European	(19–31)	-	-	test	Itkonen 2018 [87]
Clo	d1: 300<br>d2–4: 75<br>po (tab, l.d./m.d.)	Omep	d4+1h: 40<br>po (-, s.d.)	Omep	6	0	Asian	(20–24)	-	-	test	Chen 2009 [88]
Clo	d1: 300<br>d2–3: 75<br>po (tab, l.d./m.d.)	Pio	d1+1h: 15<br>po (tab, s.d.)	Pio	10	40	European	(20–35)	-	-	test	Itkonen 2016 [89]
Clo	d1: 300<br>d2–3: 75<br>po (tab, l.d./m.d.)	Repa	d1+1h: 0.25<br>d3+1h: 0.25<br>po (tab, s.d.)	Repa	9	44	European	24±4<br>(19–30)	68±14<br>(49–100)	175±10<br>(156–192)	training	Tornio 2014 [48]

-: not given, b.i.d.: maintenance dose (twice daily), Bupro: bupropion, cap: capsule, Clo: clopidogrel, Clo-AM: clopidogrel thiol H4, d: day, F: females, l.d.: loading dose, m.d.: maintenance dose (once daily), Monte: montelukast, n: number of participants, OH-Bupro: hydroxybupropion, Omep: omeprazole, Pio: pioglitazone, po: peroral, Repa: repaglinide, Rifa: rifampicin, s.d.: single dose, SR: sustained release formualtion, tab: tablet; values for age, weight and height are presented as mean ± standard deviation (range).

S4.3 Implemented Interaction Processes

Sections S4.3.1 to S4.3.6 provide an overview per drug–drug interaction (DDI) partner of the respective metabolic and transport pathways described in the literature as well as of enzymes/transporters inhibited/induced by said DDI partner. Table S11 summarizes the interaction processes implemented in the final DDI network. The precise values of the incorporated interaction parameters can be found for the DDI partners in Section S4.4 and for clopidogrel and its metabolites in Table S3.

S4.3.1 Bupropion

The antidepressant and smoking cessation agent bupropion is primarily hydroxylated to hydroxybupropion (OH-Bupro) via cytochrome P450 (CYP) 2B6, with additional metabolization to threo-/erythrohydrobupropion via 11β-hydroxysteroid dehydrogenase [90–92]. Bupropion is listed by the United States Food and Drug Administration (FDA) as sensitive clinical substrate of CYP2B6 and strong clinical inhibitor of CYP2D6 [93]. Table S12 lists the drug-dependent parameters used in the bupropion parent-metabolite physiologically based pharmacokinetic (PBPK) model.

S4.3.2 Montelukast

Regarding the metabolism of the antiasthmatic agent montelukast, CYP2C8 showed by far the greatest influence *in vitro*, followed by CYP3A4, CYP2C9, and CYP3A5 [94]. The FDA lists montelukast as moderate sensitive clinical substrate of CYP2C8 [93]. Table S13 lists the drug-dependent parameters used in the montelukast PBPK model.

S4.3.3 Omeprazole

CYP2C19 and CYP3A4 are mainly associated with the conversion of the proton pump inhibitor omeprazole [95, 96]. The FDA lists omeprazole (R-/S-omeprazole) as sensitive clinical substrate of CYP2C19 as well as weak clinical inhibitor of CYP2C19 [93]. Table S14 lists the drug-dependent parameters for R-omeprazole and S-omeprazole used in the omeprazole PBPK model.

S4.3.4 Pioglitazone

The antidiabetic agent pioglitazone is metabolized primarily via CYP2C8, being listed by the FDA as moderate sensitive clinical substrate of CYP2C8 [93, 97]. Table S15 lists the drug-dependent parameters used in the pioglitazone PBPK model.

S4.3.5 Repaglinide

The antidiabetic agent repaglinide is listed by the FDA as sensitive clinical substrate of CYP2C8 and clinical substrate of organic-anion-transporting polypeptide (OATP) 1B1/OATP1B3 [93], while also being metabolized via CYP3A4 in addition to CYP2C8 [98]. Table S16 lists the drug-dependent parameters used in the repaglinide PBPK model.

S4.3.6 Rifampicin

The antibiotic agent rifampicin demonstrates a high DDI potential being listed by the FDA as strong clinical index inducer of CYP2C19 and CYP3A4, moderate clinical index inducer of CYP2B6, CYP2C8, and CYP2C9, moderate clinical inducer of CYP1A2, and clinical inhibitor of OATP1B1/ OATP1B3 [93]. In addition, it affects unlisted enzymes/transporters like arylacetamide deacetylase (AADAC), an enzyme responsible for rifampicin deacetylation [99]. Table S17 lists the drug-dependent parameters used in the rifampicin PBPK model.

Table S11: Implemented interaction processes

		PERPETRATOR					
VICTIM	Enzyme/ Transporter	Clo	Clo-COOH	Clo-AG	2-Oxo-Clo	R-/S-Omep	Rifa
Bupro	**CYP2B6**	**MBI**			MBI		
Monte	**CYP2C8**	CI	CI	**MBI**	CI		
	CYP2C9	CI					
	CYP3A4	MBI	CI	CI	CI		
Omep	**CYP2C19**	**MBI**			CI		
	CYP3A4	MBI	CI	CI	CI		
Pio	**CYP2C8**	CI	CI	**MBI**	CI		
Repa	**CYP2C8**	CI	CI	**MBI**	CI		
	CYP3A4	MBI	CI	CI	CI		
	OATP1B1	CI		CI	CI		
Clo	**CYP2C19**					**MBI**	
Clo	**CYP2C19**						**IND**
	**CYP3A4**						**CI, IND**
	UGT2B7						CI, IND

2-Oxo-Clo: 2-Oxo-clopidogrel, Bupro: bupropion, CI: competitive inhibition, Clo: clopidogrel, Clo-AG: clopidogrel acyl glucuronide, Clo-AM: clopidogrel thiol H4, Clo-COOH: clopidogrel carboxylic acid, CYP: cytochrome P450, IND: induction, MBI: mechanism-based inactivation, Monte: montelukast, OATP: organic-anion-transporting polypeptide, Omep: omeprazole, Pio: pioglitazone, Repa: repaglinide, Rifa: rifampicin, R-Omep: R-omeprazole, S-Omep: S-omeprazole, UGT: uridine 5'-diphospho-glucuronosyltransferase; the respective main interaction processes are highlighted in bold print.

S4.4 Drug-Dependent Parameters DDI Partner

S4.4.1 Bupropion

Table S12: Drug-dependent parameters of the bupropion PBPK model [100]

Parameter	Unit	Value	Source	Description
		**Bupropion**		
Molecular weight	g/mol	239.74	Lit.	Molecular weight
TPSA*	Å^2	29.1	Lit.	Topological polar surface area
pKa, base		8.75	Lit.	Acid dissociation constant
Solubility (pH)	mg/ml	364.56 (7.4)	Lit.	Solubility
Lipophilicity		2.70	Opt.	Lipophilicity
f_u	%	16	Lit.	Fraction unbound
11β-HSD K_M → E-BU/T-BU	µmol/l	39.10	Lit.	Michaelis-Menten constant
11β-HSD k_{cat} → E-BU	1/min	2.15	Opt.	Catalytic rate constant
11β-HSD k_{cat} → T-BU	1/min	8.18	Opt.	Catalytic rate constant
CYP2B6 K_M → OH-Bupro	µmol/l	25.80^a	Lit.	Michaelis-Menten constant
CYP2B6 k_{cat} → OH-Bupro	1/min	21.74	Opt.	Catalytic rate constant
CYP2C19 K_M → sink	µmol/l	8.30	Lit.	Michaelis-Menten constant
CYP2C19 k_{cat} → sink	1/min	2.59	Opt.	Catalytic rate constant
Binding partner K_D	µmol/l	0.44	Opt.	Dissociation constant for binding
Binding partner k_{off}	1/min	0.05	Opt.	Dissociation rate constant for binding
GFR fraction		1	Asm.	Filtered drug in the urine
EHC continuous fraction		1	Asm.	Bile fraction continously released
Intestinal permeability	cm/min	$3.30 \cdot 10^{-5}$	Opt.	Transcellular intestinal permeability
Cellular permeability	cm/min	Charge dependent Schmitt, 0.14	Calc. [54]	permeability into the cellular space
Partition coefficients		PK-Sim Standard	Calc. [55]	Organ-plasma partition coefficients
Dissolution time (Weibull)	min	100.00 (SR)	Opt.	Dissolution time (Weibull)
Dissolution shape (Weibull)		1.00 (SR)	Opt.	Dissolution shape

-: not available, *: no model parameter, listed for additional information, ^{a}: *in vitro* values corrected for binding in the assay ($f_{u,mic}$), ^{b}: adjusted due to different reference concentrations in the original model and clopidogrel model, asm.: assumed, calc.: calculated, CYP: cytochrome P450, E-BU: erythrohydrobupropion, EHC: enterohepatic circulation, GFR: glomerular filtration rate, HSD: hydroxysteroid dehydrogenase, lit.: literature, OH-Bupro: hydroxybupropion, opt.: optimized, SR: sustained release formulation, T-BU: threohydrobupropion, UGT: uridine 5'-diphospho-glucuronosyltransferase.

Table S12: Drug-dependent parameters of the bupropion PBPK model [100] *(continued)*

Parameter	Unit	Value	Source	Description
**Hydroxybupropion**				
Molecular weight	g/mol	255.74	Lit.	Molecular weight
TPSA*	Å^2	49.33	Lit.	Topological polar surface area
pKa, base		7.65	Lit.	Acid dissociation constant
Solubility (pH)	mg/ml	0.91 (7.4)	Lit.	Solubility
Lipophilicity		1.90	Opt.	Lipophilicity
f_u	%	23	Lit.	Fraction unbound
UGT2B7 K_M → sink	µmol/l	14.64^a	Lit.	Michaelis-Menten constant
UGT2B7 k_{cat} → sink	1/min	40.40^b	Opt.	Catalytic rate constant
GFR fraction		1	Asm.	Filtered drug in the urine
EHC continuous fraction		1	Asm.	Bile fraction continously released
Intestinal permeability	cm/min	$2.78 \cdot 10^{-5}$	Calc.	Transcellular intestinal permeability
Cellular permeability	cm/min	Charge dependent Schmitt, 0.01	Calc. [54]	permeability into the cellular space
Partition coefficients		Berezhkovskiy	Calc. [101]	Organ-plasma partition coefficients

-: not available, *: no model parameter, listed for additional information, ^{a}: *in vitro* values corrected for binding in the assay ($f_{u,mic}$), ^{b}: adjusted due to different reference concentrations in the original model and clopidogrel model, asm.: assumed, calc.: calculated, CYP: cytochrome P450, E-BU: erythrohydrobupropion, EHC: enterohepatic circulation, GFR: glomerular filtration rate, HSD: hydroxysteroid dehydrogenase, lit.: literature, OH-Bupro: hydroxybupropion, opt.: optimized, SR: sustained release formulation, T-BU: threohydrobupropion, UGT: uridine 5'-diphospho-glucuronosyltransferase.

Table S12: Drug-dependent parameters of the bupropion PBPK model [100] *(continued)*

Parameter	Unit	Value	Source	Description
**Erythro-/Threohydrobupropion**				
Molecular weight	g/mol	241.76	Lit.	Molecular weight
TPSA*	Å^2	32.26	Lit.	Topological polar surface area
pKa, base		9.71	Lit.	Acid dissociation constant
Solubility (pH)	mg/ml	82.98 (7.4)	Lit.	Solubility
Lipophilicity		1.76	Opt.	Lipophilicity
f_u	%	58.00	Lit.	Fraction unbound
UGT2B7 K_M → sink	µmol/l	9.33[a] (E-BU), 6.22[a] (T-BU)	Lit.	Michaelis-Menten constant
UGT2B7 k_{cat} → sink	1/min	11.03[b] (E-BU), 2.94[b] (T-BU)	Opt.	Catalytic rate constant
GFR fraction		1	Asm.	Filtered drug in the urine
EHC continuous fraction		1	Asm.	Bile fraction continously released
Intestinal permeability	cm/min	$2.64 \cdot 10^{-5}$	Calc.	Transcellular intestinal permeability
Cellular permeability	cm/min	Charge dependent Schmitt, 0.01	Calc. [54]	permeability into the cellular space
Partition coefficients		Berezhkovskiy	Calc. [101]	Organ-plasma partition coefficients

-: not available, *: no model parameter, listed for additional information, [a]: *in vitro* values corrected for binding in the assay ($f_{u,mic}$), [b]: adjusted due to different reference concentrations in the original model and clopidogrel model, asm.: assumed, calc.: calculated, CYP: cytochrome P450, E-BU: erythrohydrobupropion, EHC: enterohepatic circulation, GFR: glomerular filtration rate, HSD: hydroxysteroid dehydrogenase, lit.: literature, OH-Bupro: hydroxybupropion, opt.: optimized, SR: sustained release formulation, T-BU: threohydrobupropion, UGT: uridine 5'-diphospho-glucuronosyltransferase.

S4.4.2 Montelukast

Table S13: Drug-dependent parameters of the montelukast PBPK model [102]

Parameter	Unit	Value	Source	Description
		**Montelukast**		
Molecular weight	g/mol	586.20	Lit.	Molecular weight
TPSA*	$Å^2$	70.42	Lit.	Topological polar surface area
pKa, acid		4.40	Lit.	Acid dissociation constant
Solubility (pH)	mg/ml	$8.2 \cdot 10^{-6}$ (7.0)	Lit.	Solubility
Lipophilicity		3.32	Opt.	Lipophilicity
f_u	%	$1.8 \cdot 10^{-3}$	Lit.	Fraction unbound
CYP2C8 CL → sink	l/µmol/min	3.60	Lit.	Specific clearance
CYP2C9 CL → sink	l/µmol/min	0.48	Lit.	Specific clearance
CYP3A4 CL → sink	l/µmol/min	1.80	Lit.	Specific clearance
CYP3A5 CL → sink	l/µmol/min	0.16	Lit.	Specific clearance
GFR fraction		1	Lit.	Filtered drug in the urine
EHC continuous fraction		1	Asm.	Bile fraction continously released
Intestinal permeability	cm/min	0.08	Opt.	Transcellular intestinal permeability
Cellular permeability	cm/min	PK-Sim Standard, $1.84 \cdot 10^{-3}$	Calc. [55]	permeability into the cellular space
Partition coefficients		Rodgers + Rowland	Calc. [77, 78]	Organ-plasma partition coefficients
Dissolution time (Weibull)	min	130.79	Opt.	Dissolution time (50%)
Dissolution shape (Weibull)		1.31	Opt.	Dissolution shape

*: no model parameter, listed for additional information, asm.: assumed, calc.: calculated, CYP: cytochrome P450, EHC: enterohepatic circulation, GFR: glomerular filtration rate, lit.: literature, opt.: optimized.

S4.4.3 Omeprazole

Table S14: Drug-dependent parameters of the omeprazole PBPK model [103]

Parameter	Unit	Value	Value	Source	Description
		**S-Omeprazole**	**R-Omeprazole**		
Molecular weight	g/mol	345.42	345.42	Lit.	Molecular weight
TPSA*	Å^2	77.1	77.1	Lit.	Topological polar surface area
pKa, base		4.77	4.77	Lit.	Acid dissociation constant
pKa, acid		9.29	9.29	Lit.	Acid dissociation constant
Solubility (pH)	mg/ml	0.36 (7.0)	0.36 (7.0)	Lit.	Solubility
Lipophilicity		1.68	1.68	Opt.	Lipophilicity
f_u	%	3	4	Lit.	Fraction unbound
CYP3A4 CL → sink	1/min	0.37	0.16	Opt.	Specific clearance
CYP2C19 CL → sink	1/min	9.08^a	33.28^a	Opt.	Specific clearance
CL_{hep} → sink	1/min	0.03	0.03	Lit.	Unspecific hepatic clearance
EHC continuous fraction		1	1	Asm.	Bile fraction continously released
Intestinal permeability	cm/min	$9.79 \cdot 10^{-5}$	$9.79 \cdot 10^{-5}$	Opt.	Transcellular intestinal permeability
Cellular permeability	cm/min	PK-Sim Standard, $8.18 \cdot 10^{-4}$	PK-Sim Standard, $8.18 \cdot 10^{-4}$	Calc. [55]	permeability into the cellular space
Partition coefficients		Rodgers + Rowland	Rodgers + Rowland	Calc. [77, 78]	Organ-plasma partition coefficients
Dissolution time (Weibull)	min	41.65	41.65	Opt.	Dissolution time (50%)
Dissolution shape (Weibull)		1.02	1.02	Opt.	Dissolution shape
Lag time (Weibull)	min	30	30	Asm.	Dissolution lag time
CYP2C19 K_I	µmol/l	0.3	1.6	Lit.	Conc. for half-maximal inactivation (MBI)
CYP2C19 k_{inact}	1/h	5	4	Lit.	Maximum inactivation rate constant (MBI)

*: no model parameter, listed for additional information, ^{a}: subsequently adjusted due to modification of intestinal cytochrome P450 2C19 expression, asm.: assumed, calc.: calculated, conc.: concentration, CYP: cytochrome P450, lit.: literature, MBI: mechanism-based inactivation, opt.: optimized.

S4.4.4 Pioglitazone

Table S15: Drug-dependent parameters of the pioglitazone PBPK model [105]

Parameter	Unit	Value	Source	Description
		**Pioglitazone**		
Molecular weight	g/mol	356.40	Lit.	Molecular weight
TPSA*	Å^2	68.29	Lit.	Topological polar surface area
pKa, base		5.80	Lit.	Acid dissociation constant
pKa, acid		6.40	Lit.	Acid dissociation constant
Solubility (pH)	g/l	0.02 (6.5)	Lit.	Solubility
Lipophilicity		2.81	Opt.	Lipophilicity
f_u	%	0.21	Opt.	Fraction unbound
CYP2C8 $K_M \rightarrow$ sink	µmol/l	21.00	Lit.	Michaelis-Menten constant
CYP2C8 $k_{cat} \rightarrow$ sink	1/min	68.09	Opt.	Catalytic rate constant
$CL_{hep} \rightarrow$ sink	1/min	2.14	Opt.	Unspecific hepatic clearance
GFR fraction		1	Asm.	Filtered drug in the urine
EHC continuous fraction		1	Asm.	Bile fraction continously released
Intestinal permeability	cm/min	$4.38 \cdot 10^{-5}$	Opt.	Transcellular intestinal permeability
Cellular permeability	cm/min	PK-Sim Standard, $9.07 \cdot 10^{-3}$	Calc. [55]	Permeability into the cellular space
Partition coefficients		Berezhkovskiy	Calc. [101]	Organ-plasma partition coefficients
Formulation		Tablet^a	Lit.	Formulation used in predictions

-: not available, *: no model parameter, listed for additional information, ^{a}: tablet dissolution profile from literature [104], asm.: assumed, calc.: calculated, CYP: cytochrome P450, EHC: enterohepatic circulation, GFR: glomerular filtration rate, opt.: optimized.

S4.4.5 Repaglinide

Table S16: Drug-dependent parameters of the repaglinide PBPK model [105]

Parameter	Unit	Value	Source	Description
		**Repaglinide**		
Molecular weight	g/mol	452.60	Lit.	Molecular weight
TPSA*	$Å^2$	78.87	Lit.	Topological polar surface area
pKa, base		6.01	Lit.	Acid dissociation constant
pKa, acid		4.16	Lit.	Acid dissociation constant
Solubility (pH)	g/l	0.14 (7.4)	Lit.	Solubility
Lipophilicity		2.72	Opt.	Lipophilicity
f_u	%	2.9	Opt.	Fraction unbound
CYP2C8 K_M → sink	µmol/l	2.8	Lit.	Michaelis-Menten constant
CYP2C8 k_{cat} → sink	1/min	4.56	Opt.	Catalytic rate constant
CYP3A4 K_M → sink	µmol/l	15.6	Lit.	Michaelis-Menten constant
CYP3A4 k_{cat} → sink	1/min	0.86	Opt.	Catalytic rate constant
OATP1B1 K_M	µmol/l	12.8	Lit.	Michaelis-Menten constant
OATP1B1 k_{cat}	1/min	22860.57[a]	Opt.	Transport rate constant
OATP1B3 K_M	µmol/l	12.8	Lit.	Michaelis-Menten constant
OATP1B3 k_{cat}	1/min	551.24	Opt.	Transport rate constant
GFR fraction		1	Asm.	Filtered drug in the urine
EHC continuous fraction		1	Asm.	Bile fraction continously released
Intestinal permeability	cm/min	$2.02 \cdot 10^{-5}$	Opt.	Transcellular intestinal permeability
Cellular permeability	cm/min	Charge dependent Schmitt, 0.04	Opt. [54]	Permeability into the cellular space
Partition coefficients		Schmitt	Calc. [75]	Organ-plasma partition coefficients
Formulation		Tablet[b]	Lit.	Formulation used in predictions

-: not available, *: no model parameter, listed for additional information, [a]: adjusted due to different reference concentrations in the original model and clopidogrel model, [b]: tablet dissolution profile from literature [106], asm.: assumed, calc.: calculated, CYP: cytochrome P450, EHC: enterohepatic circulation, GFR: glomerular filtration rate, lit.: literature, OATP: organic-anion-transporting polypeptide, opt.: optimized.

S4.4.6 Rifampicin

Table S17: Drug-dependent parameters of the rifampicin PBPK model [9]

Parameter	Unit	Value	Source	Description
		**Rifampicin**		
Molecular weight	g/mol	822.94	Lit.	Molecular weight
TPSA*	Å^2	220.15	Lit.	Topological polar surface area
pKa, base		7.90	Lit.	Acid dissociation constant
pKa, acid		1.70	Lit.	Acid dissociation constant
Solubility (pH)	g/l	2.80 (7.5)	Lit.	Solubility
Lipophilicity		2.50	Opt.	Lipophilicity
f_u	%	17.00	Lit.	Fraction unbound
Blood/plasma ratio		0.89	Calc.	Blood/plasma ratio
AADAC K_M → sink	µmol/l	195.10	Lit.	Michaelis-Menten constant
AADAC k_{cat} → sink	1/min	9.87	Opt.	Catalytic rate constant
OATP1B1 K_M	µmol/l	1.50	Lit.	Michaelis-Menten constant
OATP1B1 k_{cat}	1/min	111.37[a]	Opt.	Transport rate constant
P-gp K_M	µmol/l	55.00	Lit.	Michaelis-Menten constant
P-gp k_{cat}	1/min	0.61	Opt.	Transport rate constant
GFR fraction		1	Asm.	Filtered drug in the urine
EHC continuous fraction		1	Asm.	Bile fraction continously released
Intestinal permeability	cm/min	$1.24 \cdot 10^{-5}$	Opt.	Transcellular intestinal permeability
Cellular permeability	cm/min	PK-Sim Standard, $2.93 \cdot 10^{-5}$	Calc. [55]	Permeability into the cellular space
Partition coefficients		Rodgers + Rowland	Calc. [77, 78]	Organ-plasma partition coefficients
Formulation		Solution		Formulation used in predictions

-: not available, *: no model parameter, listed for additional information, [a]: adjusted due to different reference concentrations in the original model and clopidogrel model, AADAC: arylacetamide deacetylase, asm.: assumed, calc.: calculated, CI: competitive inhibition, conc.: concentration, CYP: cytochrome P450, EHC: enterohepatic circulation, GFR: glomerular filtration rate, lit.: literature, OATP: organic-anion-transporting polypeptide, opt.: optimized, P-gp: P-glycoprotein, UGT: uridine 5'-diphospho-glucuronosyltransferase.

Table S17: Drug-dependent parameters of the rifampicin PBPK model [9] *(continued)*

Parameter	Unit	Value	Source	Description
Induction EC_{50}	µmol/l	0.34	Lit.	Conc. for half-maximal induction
AADAC E_{max}		0.99	Opt.	Maximum in vivo induction effect
CYP2C19 E_{max}		2.10	Lit.	Maximum in vivo induction effect
CYP3A4 E_{max}		9.00	Lit.	Maximum in vivo induction effect
CYP3A4 K_i	µmol/l	18.50	Lit.	Conc. for half-maximal inhibition (CI)
OATP1B1 E_{max}		0.38	Opt.	Maximum in vivo induction effect
OATP1B1 K_i	µmol/l	0.48	Lit.	Conc. for half-maximal inhibition (CI)
P-gp E_{max}		2.50	Lit.	Maximum in vivo induction effect
P-gp K_i	µmol/l	169.00	Lit.	Conc. for half-maximal inhibition (CI)
UGT2B7 E_{max}		1.79	Lit.	Maximum in vivo induction effect
UGT2B7 K_i	µmol/l	554.87	Lit.	Conc. for half-maximal inhibition (CI)

-: not available, *: no model parameter, listed for additional information, ^{a}: adjusted due to different reference concentrations in the original model and clopidogrel model, AADAC: arylacetamide deacetylase, asm.: assumed, calc.: calculated, CI: competitive inhibition, conc.: concentration, CYP: cytochrome P450, EHC: enterohepatic circulation, GFR: glomerular filtration rate, lit.: literature, OATP: organic-anion-transporting polypeptide, opt.: optimized, P-gp: P-glycoprotein, UGT: uridine 5'-diphospho-glucuronosyltransferase.

S4.4.7 Partition Coefficients Intracellular : Plasma DDI Partner

Table S18: Partition coefficients between intracellular space and plasma of the DDI partners' PBPK models

Tissue	Bupro[a]	OH-Bupro[b]	E-BU/T-BU[b]	Monte[c]	R-Omep[c]	S-Omep[c]	Pio[b]	Repa[d]	Rifa[c]
Bone	22.15	1.59	2.59	0.10	0.15	0.13	0.34	0.14	0.53
Brain	9.14	1.69	2.66	0.05	0.15	0.13	0.50	0.93	0.36
Fat	64.58	0.45	0.12	0.05	0.17	0.14	0.08	1.09	0.32
Gonads	2.84	0.65	0.86	0.05	0.10	0.08	0.43	0.46	1.87
Heart	8.48	0.75	1.06	0.16	0.20	0.19	0.42	0.89	1.73
Kidney	4.62	0.93	1.36	0.13	0.19	0.17	0.45	0.68	3.79
Stomach	5.36	1.39	2.17	0.16	0.26	0.23	0.45	0.39	1.86
Small intestine	5.36	1.39	2.17	0.16	0.26	0.23	0.45	0.39	1.86
Large intestine	5.36	1.39	2.17	0.16	0.26	0.23	0.45	0.39	1.86
Liver periportal	6.01	1.21	1.85	0.09	0.15	0.13	0.46	0.84	3.43
Liver pericentral	6.01	1.21	1.85	0.09	0.15	0.13	0.46	0.84	3.43
Lung	1.27	0.59	0.77	0.21	0.27	0.26	0.44	0.37	2.96
Muscle	1.50	0.92	1.35	0.06	0.14	0.12	0.44	0.16	1.85
Pancreas	6.79	1.21	1.85	0.06	0.16	0.14	0.44	0.23	1.29
Skin	8.68	1.00	1.50	0.28	0.41	0.37	0.42	0.51	1.06
Spleen	1.76	0.94	1.38	0.10	0.13	0.13	0.45	0.28	2.41
Saliva	0.16	0.23	0.58	$1.80 \cdot 10^{-3}$	0.04	0.03	$2.07 \cdot 10^{-3}$	0.03	0.17

[a]: estimated via PK-Sim Standard [55], [b]: estimated via Berezhkovskiy [101], [c]: estimated via Rodgers + Rowland [77, 78], [d]: estimated via Schmitt [75], Bupro: bupropion, E-BU: erythrohydrobupropion, Monte: montelukast, OH-Bupro: hydroxy-bupropion, Pio: pioglitazone, Repa: repaglinide, Rifa: rifampicin, R-Omep: R-omeprazole, S-Omep: S-omeprazole, T-BU: threohydrobupropion.

S4.5 Plasma Concentration-Time Profiles (Semilogarithmic)

S4.5.1 Clopidogrel as Victim

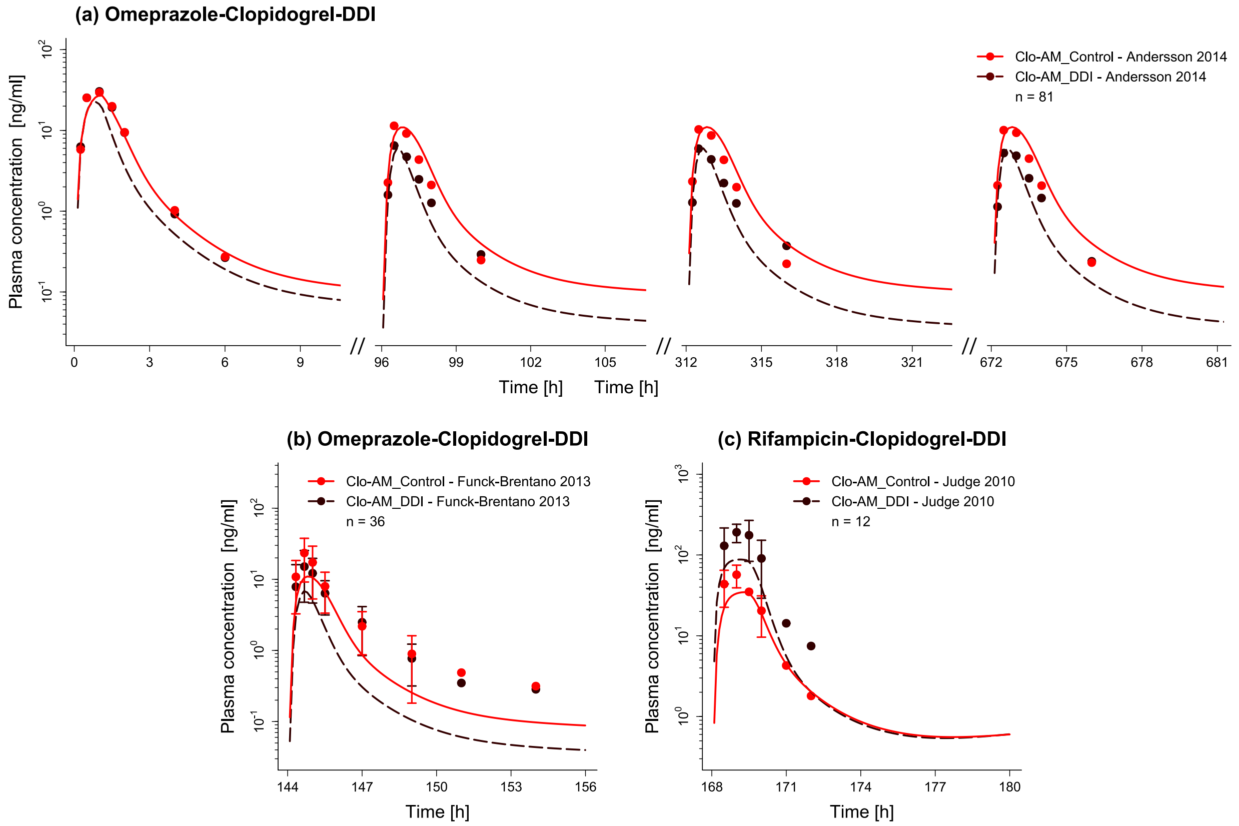

Figure S33: Drug–drug interaction network evaluation with clopidogrel as victim. Presented are predicted plasma concentration-time profiles (semilogarithmic plots) of Clo-AM with (DDI) and without (Control) intake of the respective perpetrator drug ((a–b) omeprazole, (c) rifampicin), alongside corresponding observed data [83–85]. Dashed (DDI) and solid (Control) lines represent the model predictions, while corresponding observed data are shown as symbols (± standard deviation, if available). Clo-AM: clopidogrel thiol H4, DDI: drug–drug interaction, n: number of participants.

S4.5.2 Clopidogrel as Perpetrator

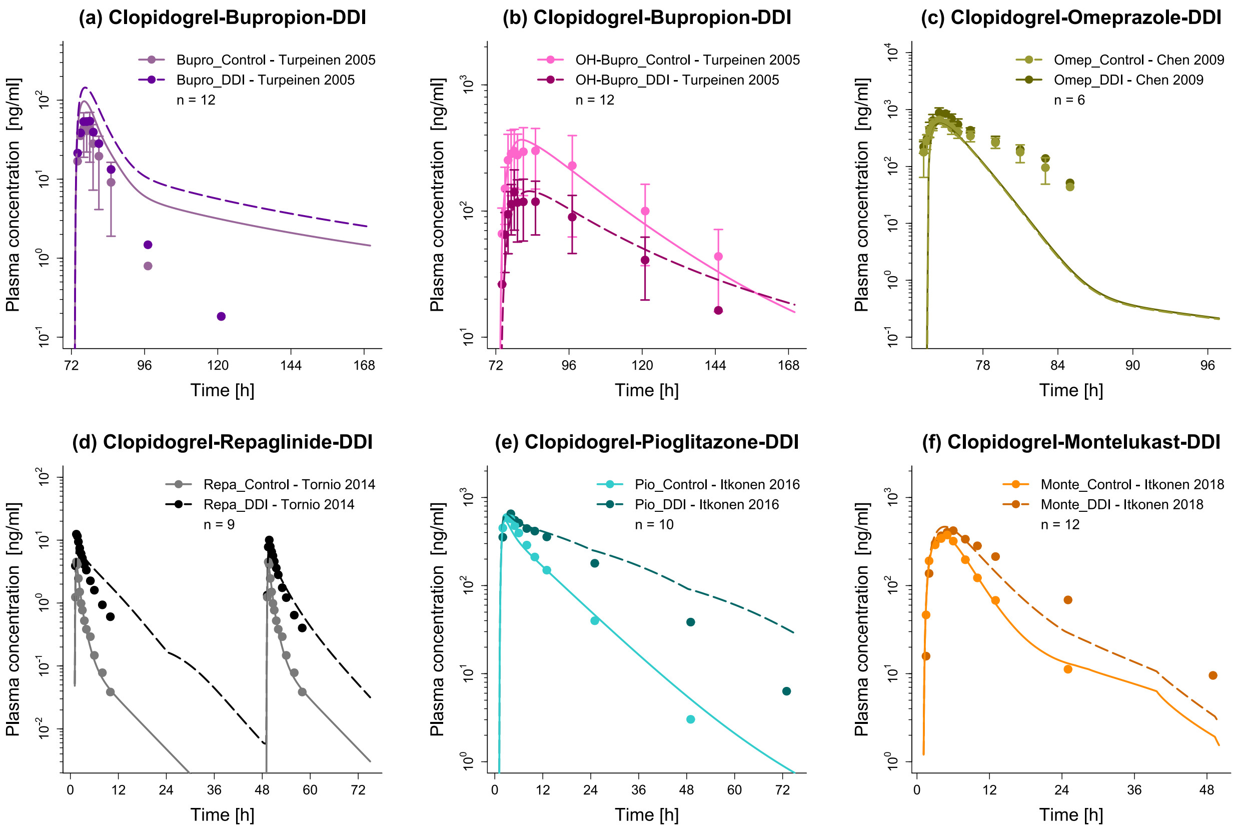

Figure S34: Drug–drug interaction network evaluation with clopidogrel as perpetrator. Presented are predicted plasma concentration-time profiles (semilogarithmic plots) of the respective victim with (DDI) and without (Control) intake of clopidogrel ((a) bupropion, (b) hydroxybupropion, (c) omeprazole, (d) repaglinide, (e) pioglitazone, (f) montelukast), alongside corresponding observed data [48, 86–89]. Dashed (DDI) and solid (Control) lines represent the model predictions, while corresponding observed data are shown as symbols (± standard deviation, if available). Bupro: bupropion, DDI: drug–drug interaction, Monte: montelukast, n: number of participants, OH-Bupro: hydroxybupropion, Omep: omeprazole, Pio: pioglitazone, Repa: repaglinide.

S4.6 Plasma Concentration-Time Profiles (Linear)

S4.6.1 Clopidogrel as Victim

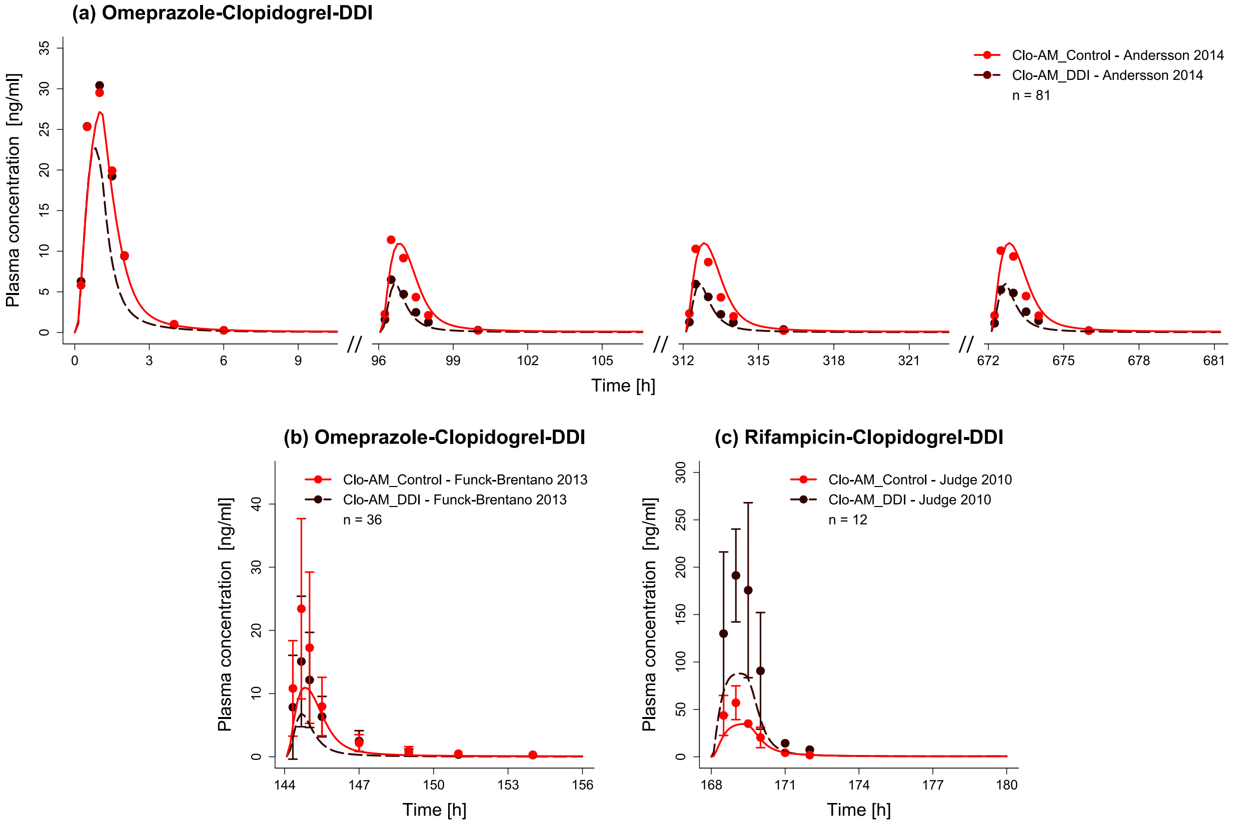

Figure S35: Drug–drug interaction network evaluation with clopidogrel as victim. Presented are predicted plasma concentration-time profiles (linear plots) of Clo-AM with (DDI) and without (Control) intake of the respective perpetrator drug ((a–b) omeprazole, (c) rifampicin), alongside corresponding observed data [83–85]. Dashed (DDI) and solid (Control) lines represent the model predictions, while corresponding observed data are shown as symbols (± standard deviation, if available). Clo-AM: clopidogrel thiol H4, DDI: drug–drug interaction, n: number of participants.

S4.6.2 Clopidogrel as Perpetrator

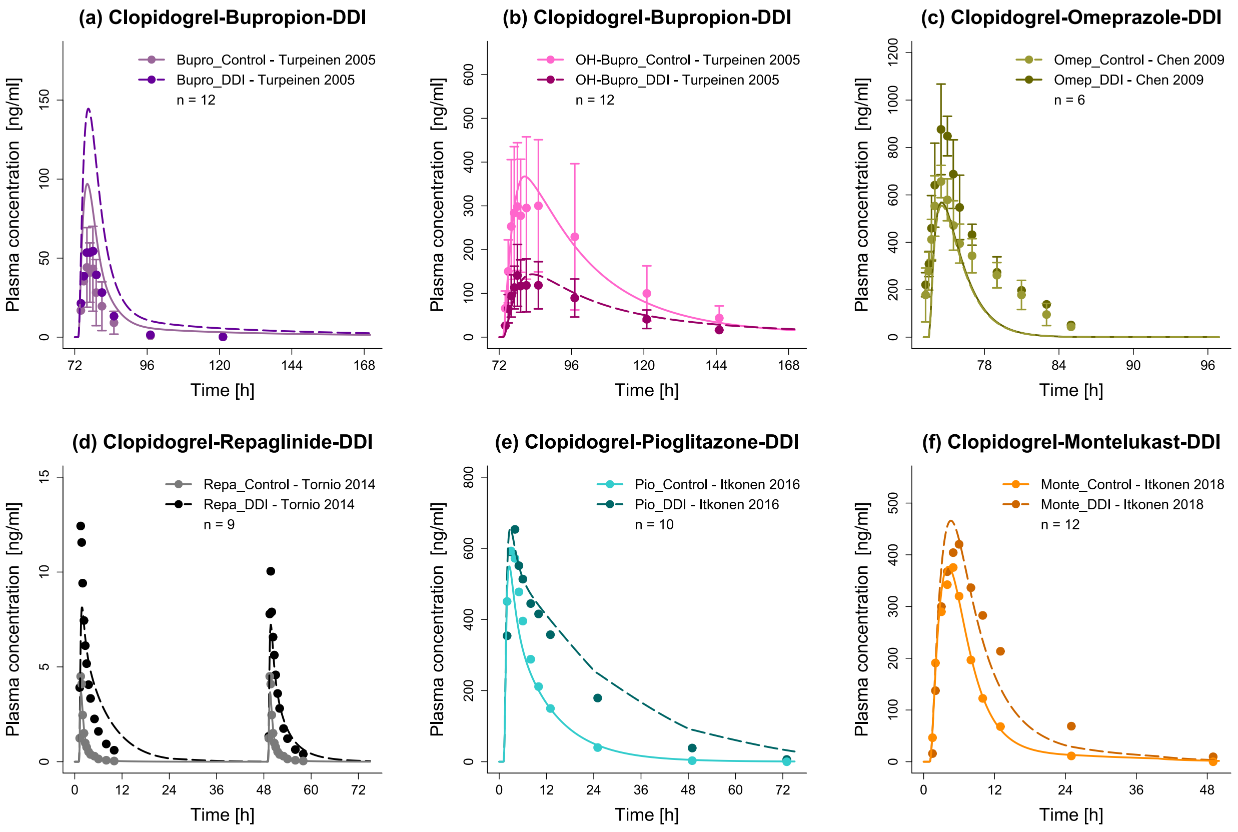

Figure S36: Drug–drug interaction network evaluation with clopidogrel as perpetrator. Presented are predicted plasma concentration-time profiles (linear plots) of the respective victim with (DDI) and without (Control) intake of clopidogrel ((a) bupropion, (b) hydroxybupropion, (c) omeprazole, (d) repaglinide, (e) pioglitazone, (f) montelukast), alongside corresponding observed data [48, 86–89]. Dashed (DDI) and solid (Control) lines represent the model predictions, while corresponding observed data are shown as symbols (± standard deviation, if available). Bupro: bupropion, DDI: drug–drug interaction, Monte: montelukast, n: number of participants, OH-Bupro: hydroxybupropion, Omep: omeprazole, Pio: pioglitazone, Repa: repaglinide.

S4.7 DDI AUC_{last} and DDI C_{max} Ratio Goodness-of-Fit Plots

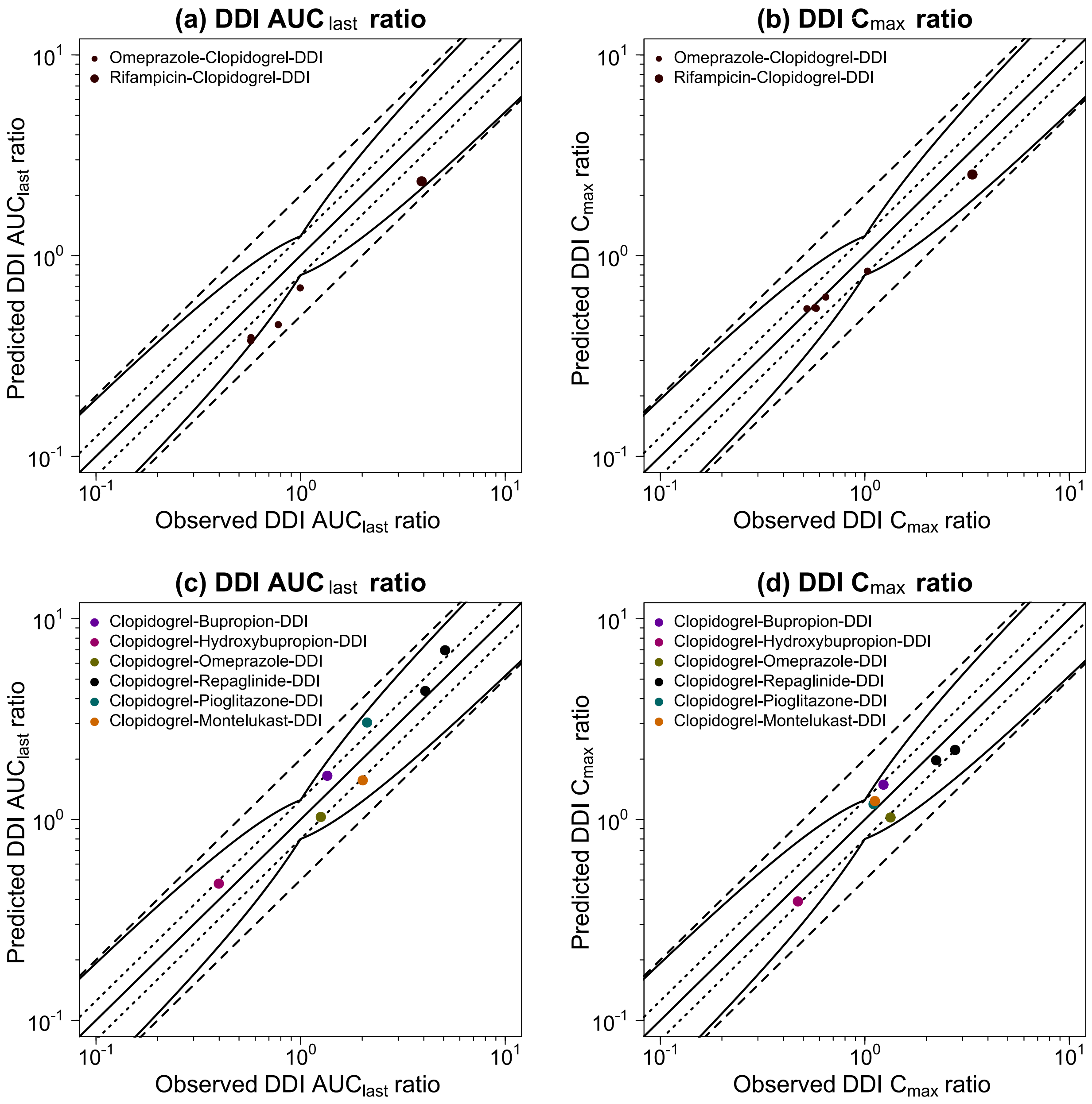

Figure S37: Drug–drug interaction network evaluation with clopidogrel as (a–b) victim and (c–d) perpetrator. Predicted versus observed (a,c) DDI AUC_{last} and (b,d) DDI C_{max} ratios of the respective victim are shown with the solid line representing the line of identity, dotted lines indicating 1.25-fold and dashed lines 2-fold deviation from the respective observed value [83–85], along with the curved lines marking the prediction success limits proposed by Guest et al. [81] (including 20% variability to account for uncertainties in observed ratios). AUC_{last}: area under the plasma concentration-time curve determined between first and last concentration measurements, C_{max}: maximum plasma concentration, DDI: drug–drug interaction.

S4.8 Predicted and Observed DDI AUC_{last} and DDI C_{max} Ratios with Mean GMFEs

Table S19: Predicted versus observed DDI AUC_{last} and DDI C_{max} ratios, along with GMFE values

Perpetrator	Perpetrator application [mg]	Victim	Victim application [mg]	Compound measured	n	Dataset	DDI AUC_{last} ratio			DDI C_{max} ratio			Reference
							Pred	Obs	Pred/Obs	Pred	Obs	Pred/Obs	
Omep	d1–29: 80<br>po (cap, m.d.)	Clo	d1: 300<br>d2–29: 75<br>po (tab, l.d./m.d.)	Clo-AM	81	test	0.69<br>0.39<br>0.38<br>0.38	1.00<br>0.57<br>0.57<br>0.57	0.69 (D1)<br>0.68 (D5)<br>0.66 (D14)<br>0.66 (D29)	0.84<br>0.55<br>0.55<br>0.54	1.03<br>0.57<br>0.58<br>0.52	0.81 (D1)<br>0.97 (D5)<br>0.94 (D14)<br>1.04 (D29)	Andersson 2014 [83]
Omep	d1–7: 20<br>po (-, m.d.)	Clo	d1–7: 75<br>po (-, m.d.)	Clo-AM	36	test	0.45	0.78	0.58 (D7)	0.62	0.64	0.97 (D7)	Funck-Brentano 2013 [84]
Rifa	d1–15: 300<br>po (-, b.i.d.)	Clo	d8: 600<br>d9–15: 75<br>po (-, l.d./m.d.)	Clo-AM	12	test	2.35	3.90	0.60 (D8)	2.54	3.36	0.76 (D8)	Judge 2010 [85]
				Mean GMFE (range)					1.55 (1.44–1.72)			1.12 (1.03–1.32)	
				GMFE $\leq$ 2					6/6			6/6	

-: not given, AUC_{last}: area under the plasma concentration-time curve determined between first and last concentration measurements, b.i.d.: maintenance dose (twice daily), Bupro: bupropion, cap: capsule, Clo: clopidogrel, Clo-AM: clopidogrel thiol H4, C_{max}: maximum plasma concentration, d: day, D: day of pharmacokinetic sampling, l.d.: loading dose, m.d.: maintenance dose (once daily), Monte: montelukast, n: number of participants, obs: observed, OH-Bupro: hydroxybupropion, Omep: omeprazole, Pio: pioglitazone, po: peroral, pred: predicted, Repa: repaglinide, Rifa: rifampicin, s.d.: single dose, SR: sustained release formualtion, tab: tablet.

Table S19: Predicted versus observed DDI AUC_{last} and DDI C_{max} ratios, along with GMFE values *(continued)*

Perpetrator	Perpetrator application [mg]	Victim	Victim application [mg]	Compound measured	n	Dataset	DDI AUC_{last} ratio			DDI C_{max} ratio			Reference
							Pred	Obs	Pred/Obs	Pred	Obs	Pred/Obs	
Clo	d1–4: 75<br>po (tab, m.d.)	Bupro	d4+1h: 150<br>po (tab SR, s.d.)	Bupro<br>OH-Bupro	12	test	1.65<br>0.48	1.35<br>0.40	1.22 (D4)<br>1.20 (D4)	1.49<br>0.39	1.23<br>0.47	1.21 (D4)<br>0.83 (D4)	Turpeinen 2005 [86]
Clo	d1: 300<br>d2: 75<br>po (tab, l.d./m.d.)	Monte	d1+1h: 10<br>po (tab, s.d.)	Monte	12	test	1.57	2.01	0.78 (D1)	1.24	1.12	1.10 (D1)	Itkonen 2018 [87]
Clo	d1: 300<br>d2–4: 75<br>po (tab, l.d./m.d.)	Omep	d4+1h: 40<br>po (-, s.d.)	Omep	6	test	1.03	1.26	0.82 (D4)	1.02	1.34	0.77 (D4)	Chen 2009 [88]
Clo	d1: 300<br>d2–3: 75<br>po (tab, l.d./m.d.)	Pio	d1+1h: 15<br>po (tab, s.d.)	Pio	10	test	3.04	2.11	1.44 (D1)	1.19	1.10	1.08 (D1)	Itkonen 2016 [89]
Clo	d1: 300<br>d2–3: 75<br>po (tab, l.d./m.d.)	Repa	d1+1h: 0.25<br>d3+1h: 0.25<br>po (tab, s.d.)	Repa	9	training	6.97<br>4.36	5.08<br>4.07	1.37 (D1)<br>1.07 (D3)	2.22<br>1.97	2.76<br>2.23	0.80 (D1)<br>0.88 (D3)	Tornio 2014 [48]
				Mean GMFE (range)					1.26 (1.07–1.44)			1.18 (1.08–1.30)	
				GMFE $\leq$ 2					7/7			7/7	
				Overall mean GMFE (range)					1.39 (1.07–1.72)			1.15 (1.03–1.32)	
				GMFE $\leq$ 2					13/13			13/13	

-: not given, AUC_{last}: area under the plasma concentration-time curve determined between first and last concentration measurements, b.i.d.: maintenance dose (twice daily), Bupro: bupropion, cap: capsule, Clo: clopidogrel, Clo-AM: clopidogrel thiol H4, C_{max}: maximum plasma concentration, d: day, D: day of pharmacokinetic sampling, l.d.: loading dose, m.d.: maintenance dose (once daily), Monte: montelukast, n: number of participants, obs: observed, OH-Bupro: hydroxybupropion, Omep: omeprazole, Pio: pioglitazone, po: peroral, pred: predicted, Repa: repaglinide, Rifa: rifampicin, s.d.: single dose, SR: sustained release formualtion, tab: tablet.

Bibliography

[1] Nikolay Kolesnikov, Emma Hastings, Maria Keays, Olga Melnichuk, Y. Amy Tang, Eleanor Williams, Miroslaw Dylag, Natalja Kurbatova, Marco Brandizi, Tony Burdett, Karyn Megy, Ekaterina Pilicheva, Gabriella Rustici, Andrew Tikhonov, Helen Parkinson, Robert Petryszak, Ugis Sarkans, and Alvis Brazma. ArrayExpress update-simplifying data submissions. *Nucleic Acids Research*, 43(D1):D1113–D1116, 2015. ISSN 13624962.

[2] Masuhiro Nishimura and Shinsaku Naito. Tissue-specific mRNA expression profiles of human phase I metabolizing enzymes except for cytochrome P450 and phase II metabolizing enzymes. *Drug metabolism and pharmacokinetics*, 21(5):357–374, 2006. ISSN 13474367. doi: 10.2133/dmpk.21.357.

[3] Stephen J. Godin, J. Allen Crow, Edward J. Scollon, Michael F. Hughes, Michael J. DeVito, and Matthew K. Ross. Identification of rat and human cytochrome P450 isoforms and a rat serum esterase that metabolize the pyrethroid insecticides deltamethrin and esfenvalerate. *Drug Metabolism and Disposition*, 35(9):1664–1671, 2007. ISSN 00909556. doi: 10.1124/dmd.107.015388.

[4] A. David Rodrigues. Integrated cytochrome P450 reaction phenotyping. Attempting to bridge the gap between cDNA-expressed cytochromes P450 and native human liver microsomes. *Biochemical Pharmacology*, 57(5):465–480, 1999. ISSN 00062952. doi: 10.1016/S0006-2952(98)00268-8.

[5] Masuhiro Nishimura, Hiroshi Yaguti, Hiroki Yoshitsugu, and Shinsaku Naito. Tissue distribution of mRNA expression of human cytochrome P450 isoforms assessed by high-sensitivity real-time reverse transcription PCR. *Journal of the Pharmaceutical Society of Japan*, 123(5):369 – 375, 2003.

[6] National Center for Biotechnology Information (NCBI) (2010) Expressed Sequence Tags (EST) from UniGene.

[7] Bhagwat Prasad, Raymond Evers, Anshul Gupta, Cornelis E C a Hop, Laurent Salphati, Suneet Shukla, Suresh Ambudkar, and Jashvant D Unadkat. Interindividual variability in hepatic OATPs and P-glycoprotein (ABCB1) protein expression: Quantification by LC-MS/MS and influence of genotype, age and sex. *Drug metabolism and disposition: the biological fate of chemicals*, 42(1):78–88, 2014. ISSN 1521-009X.

[8] Masuhiro N Nishimura and Shinsaku N Aito. Tissue-specific mRNA expression profiles of human ATP-binding cassette and solute carrier transporter superfamilies. *Drug metabolism and pharmacokinetics*, 20 (6):452–477, 2005.

[9] Nina Hanke, Sebastian Frechen, Daniel Moj, Hannah Britz, Thomas Eissing, Thomas Wendl, and Thorsten Lehr. PBPK models for CYP3A4 and P-gp DDI prediction: A modeling network of rifampicin, itraconazole, clarithromycin, midazolam, alfentanil, and digoxin. *CPT: Pharmacometrics and Systems Pharmacology*, 7(10):647–659, oct 2018. ISSN 21638306. doi: 10.1002/psp4.12343.

[10] Guillaume Margaillan, Michèle Rouleau, John K. Fallon, Patrick Caron, Lyne Villeneuve, Véronique Turcotte, Philip C. Smith, Melanie S. Joy, and Chantal Guillemette. Quantitative profiling of Human renal UDP-glucuronosyltransferases and glucuronidation activity: A comparison of normal and tumoral kidney tissues. *Drug Metabolism and Disposition*, 43(4):611–619, 2015. ISSN 1521009X. doi: 10.1124/dmd.114.062877.

[11] Michaela Meyer, Sebastian Schneckener, Bernd Ludewig, Lars Kuepfer, and Joerg Lippert. Using expression data for quantification of active processes in physiologically based pharmacokinetic modeling. *Drug metabolism and disposition: the biological fate of chemicals*, 40(5):892–901, may 2012.

[12] Daniel Scotcher, Sarah Billington, Jay Brown, Christopher R. Jones, Colin D.A. Brown, Amin Rostami-Hodjegan, and Aleksandra Galetin. Microsomal and cytosolic scaling factors in dog and human kidney cortex and application for in vitro-in vivo extrapolation of renal metabolic clearance. *Drug Metabolism and Disposition*, 45(5):556–568, 2017. ISSN 1521009X. doi: 10.1124/dmd.117.075242.

[13] Daniel J. Cushing, Paul F. Souney, Warren D. Cooper, Gerald L. Mosher, Michael P. Adams, Stephen Machatha, Bing Zhang, and Peter R. Kowey. Pharmacokinetics and platelet aggregation inhibitory effects of a novel intravenous formulation of clopidogrel in humans. *Clinical and Experimental Pharmacology and Physiology*, 39(1):3–8, 2012. ISSN 14401681. doi: 10.1111/j.1440-1681.2011.05616.x.

[14] Simona Nicoleta Savu, Luigi Silvestro, Mariana Surmeian, Lina Remis, Yuksel Rasit, Simona Rizea Savu, and Constantin Mircioiu. Evaluation of clopidogrel conjugation metabolism: PK studies in man and mice of clopidogrel acyl glucuronide. *Drug Metabolism and Disposition*, 44(9):1490–1497, sep 2016. ISSN 1521009X. doi: 10.1124/dmd.116.071092.

[15] L. Silvestro, S. N. Savu, S. Rizea Savu, and I. Tarcomnicu. Clopidogrel pharmacokinetic: Review of early studies and novel experimental results. In James P. Alesci and Alexander Victorino, editors, *Clopidogrel: pharmacology, clinical uses and adverse effects*, pages 85 – 120. Nova Science Publishers: New York, USA, 2013. ISBN 9781629483368.

[16] Ramakrishna V.S. Nirogi, Vishwottam N. Kandikere, Manoj Shukla, Koteshwara Mudigonda, Santosh Maurya, and Ravikumar Boosi. Quantification of clopidogrel in human plasma by sensitive liquid chromatography/tandem mass spectrometry. *Rapid Communications in Mass Spectrometry*, 20(11):1695–1700, 2006. ISSN 09514198. doi: 10.1002/rcm.2497.

[17] Jian Jun Zou, Jie Tan, Hong Wei Fan, and Shao Liang Chen. Bioequivalence study of clopidogrel 75 mg tablets in healthy male volunteers. *Journal of Bioequivalence and Bioavailability*, 4(1):006–009, 2012. ISSN 09750851. doi: 10.4172/jbb.1000102.

[18] Guillermo Di Girolamo, Paola Czerniuk, Roberto Bertuola, and Guillermo A. Keller. Bioequivalence of two tablet formulations of clopidogrel in healthy argentinian volunteers: A single-dose, randomized-sequence, open-label crossover study. *Clinical Therapeutics*, 32(1):161–170, jan 2010. ISSN 1879114X. doi: 10.1016/j.clinthera.2010.01.010.

[19] Nina Brvar, Sylvain Lachance, Ann Lévesque, Marjanca Breznik, Lea Cvitkovič-Maričič, Mateja Merslavič, Iztok Grabnar, and Tatjana Mateović-Rojnik. Comparative bioavailability of two oral formulations of clopidogrel: Determination of clopidogrel and its carboxylic acid metabolite (SR26334) under fasting and fed conditions in healthy subjects. *Acta Pharmaceutica*, 64(1):45–62, 2014. ISSN 18469558. doi: 10.2478/acph-2014-0001.

[20] Gerard Patrick McGregor. Pivotal bioequivalence study of clopacin®, a generic formulation of clopidogrel 75 mg film-coated tablets. *Advances in Therapy*, 33(2):186–198, 2016. ISSN 18658652. doi: 10.1007/s12325-016-0290-0.

[21] Beom Soo Shin and Sun Dong Yoo. Determination of clopidogrel in human plasma by liquid chromatography/tandem mass spectrometry: application to a clinical pharmacokinetic study. *Biomedical Chromatography*, 21(9):883–889, 2007. doi: 10.1002/bmc.

[22] A. Robinson, J. Hillis, C. Neal, and A. C. Leary. The validation of a bioanalytical method for the determination of clopidogrel in human plasma. *Journal of Chromatography B: Analytical Technologies in the Biomedical and Life Sciences*, 848(2):344–354, apr 2007. ISSN 15700232. doi: 10.1016/j.jchromb.2006.10.076.

[23] Ho Sook Kim, Younghae Lim, Minkyung Oh, Jong Lyul Ghim, Eun Young Kim, Dong Hyun Kim, and Jae Gook Shin. The pharmacokinetic and pharmacodynamic interaction of clopidogrel and cilostazol in relation to CYP2C19 and CYP3A5 genotypes. *British Journal of Clinical Pharmacology*, 81(2):301–312, feb 2016. ISSN 13652125. doi: 10.1111/bcp.12794.

[24] Chun-hua Zhou, Meng Xu, Hai-bing Yu, Xiao-Ting Zheng, Zhang-Feng Zhong, and Lan-ton Zhang. Effects of danshen capsules on the pharmacokinetics and pharmacodynamics of clopidogrel in healthy volunteers. *Food and Chemical Toxicology*, 119(February):302–308, 2018. ISSN 18736351. doi: 10.1016/j.fct.2018.02.051.

[25] Sebastian Härtter, Regina Sennewald, Cornelia Schepers, Sybille Baumann, Holger Fritsch, and Jeffrey Friedman. Pharmacokinetic and pharmacodynamic effects of comedication of clopidogrel and dabigatran etexilate in healthy male volunteers. *European Journal of Clinical Pharmacology*, 69(3):327–339, mar 2013. ISSN 00316970. doi: 10.1007/s00228-012-1304-8.

[26] K. A. Kim, P. W. Park, S. J. Hong, and J. Y. Park. The effect of CYP2C19 polymorphism on the pharmacokinetics and pharmacodynamics of clopidogrel: A possible mechanism for clopidogrel resistance. *Clinical Pharmacology and Therapeutics*, 84(2):236–242, aug 2008. ISSN 00099236. doi: 10.1038/clpt.2008.20.

[27] Bo Hyung Kim, Jung Ryul Kim, Kyoung Soo Lim, Hyun Suk Shin, Seo Hyun Yoon, Joo Youn Cho, In Jin Jang, Sang Goo Shin, and Kyung Sang Yu. Comparative pharmacokinetics/pharmacodynamics of clopidogrel besylate and clopidogrel bisulfate in healthy Korean subjects. *Clinical Drug Investigation*, 32 (12):817–826, dec 2012. ISSN 11732563. doi: 10.1007/s40261-012-0007-3.

[28] Sung Doo Kim, Wonku Kang, Hae Won Lee, Dae Jin Park, Ju Hee Ahn, Mi Jin Kim, Eun Young Kim, Sung Wuk Kim, Hee Sook Nam, Hye Jung Na, and Young Ran Yoon. Bioequivalence and tolerability of two clopidogrel salt preparations, besylate and bisulfate: A randomized, open-label, crossover study in healthy Korean male subjects. *Clinical Therapeutics*, 31(4):793–803, 2009. ISSN 01492918. doi: 10.1016/j.clinthera.2009.04.017.

[29] M. T. Holmberg, A. Tornio, M. Neuvonen, P. J. Neuvonen, J. T. Backman, and M. Niemi. Grapefruit juice inhibits the metabolic activation of clopidogrel. *Clinical Pharmacology and Therapeutics*, 95(3): 307–313, mar 2014. ISSN 00099236. doi: 10.1038/clpt.2013.192.

[30] Luigi Silvestro, Mihaela Gheorghe, Adriana Iordachescu, Valentin Ciuca, Ariana Tudoroniu, Simona Rizea Savu, and Isabela Tarcomnicu. Development and validation of an HPLC-MS/MS method to quantify clopidogrel acyl glucuronide, clopidogrel acid metabolite, and clopidogrel in plasma samples avoiding analyte back-conversion. *Analytical and Bioanalytical Chemistry*, 401(3):1023–1034, aug 2011. ISSN 16182642. doi: 10.1007/s00216-011-5147-4.

[31] Eduardo Abib Junior, Luciana Fernandes Duarte, Moisés Luís Pirasol Vanunci, Daniela Aparecida de Oliveira, Tatiane Antonelli Stein, Renata Pereira, Antonio Ricardo Amarante, Eunice Mayumi Suenaga, and Alessandro de Carvalho Cruz. Comparative biological availability of clopidogrel formulation in healthy volunteers after a single dose administration. *Journal of Bioequivalence & Bioavailability*, 02(02): 045 – 049, 2010. doi: 10.4172/jbb.1000029.

[32] A. M. Yousef, M. Melhem, B. Xue, T. Arafat, D. K. Reynolds, and S. A. Van Wart. Population pharmacokinetic analysis of clopidogrel in healthy Jordanian subjects with emphasis optimal sampling strategy. *Biopharmaceutics and Drug Disposition*, 34(4):215–226, may 2013. ISSN 01422782. doi: 10.1002/bdd.1839.

[33] Effat Souri, Hassan Jalalizadeh, Abbas Kebriaee-Zadeh, Maral Shekarchi, Afshin Dalvandi, and : E Souri. Validated HPLC method for determination of carboxylic acid metabolite of clopidogrel in human plasma and its application to a pharmacokinetic study. *Biomedical Chromatography*, 20:1309–1314, 2006. doi: 10.1002/bmc.

[34] Hanna Ksycinska, Piotr Rudzki, and Mirosława Bukowska-Kiliszek. Determination of clopidogrel metabolite (SR26334) in human plasma by LC-MS. *Journal of Pharmaceutical and Biomedical Analysis*, 41(2): 533–539, may 2006. ISSN 07317085. doi: 10.1016/j.jpba.2005.11.035.

[35] Gholamreza Bahrami, Bahareh Mohammadi, and Sajjad Sisakhtnezhad. High-performance liquid chromatographic determination of inactive carboxylic acid metabolite of clopidogrel in human serum: Application to a bioequivalence study. *Journal of Chromatography B: Analytical Technologies in the Biomedical and Life Sciences*, 864(1-2):168–172, mar 2008. ISSN 15700232. doi: 10.1016/j.jchromb.2008.01.049.

[36] David S. Small, Nagy A. Farid, Christopher D. Payne, Govinda J. Weerakkody, Ying G. Li, John T. Brandt, Daniel E. Salazar, and Kenneth J. Winters. Effects of the proton pump inhibitor lansoprazole on the pharmacokinetics and pharmacodynamics of prasugrel and clopidogrel. *Journal of Clinical Pharmacology*, 48(4):475–484, apr 2008. ISSN 00912700. doi: 10.1177/0091270008315310.

[37] Xiaojiao Li, Cai Liu, Xiaoxue Zhu, Haijing Wei, Hong Zhang, Hong Chen, Guiling Chen, Deming Yang, Hongbin Sun, Zhenwei Shen, Yifan Zhang, Wei Li, Jin Yang, Yongqiang Liu, Xiaojuan Lai, Yanchun Gong, Xuefang Liu, Yongguo Li, Dafang Zhong, Junqi Niu, Bin Liu, and Yanhua Ding. Evaluation of tolerability, pharmacokinetics and pharmacodynamics of vicagrel, a novel P2Y12 antagonist, in healthy Chinese volunteers. *Frontiers in Pharmacology*, 9(JUN):1–12, 2018. ISSN 16639812. doi: 10.3389/fphar.2018.00643.

[38] Kazuo Umemura and Takayuki Iwaki. The pharmacokinetics and pharmacodynamics of prasugrel and clopidogrel in healthy Japanese volunteers. *Clinical Pharmacology in Drug Development*, 5(6):480–487, nov 2016. ISSN 21607648. doi: 10.1002/cpdd.259.

[39] Makoto Takahashi, Henrianna Pang, Kiyoshi Kawabata, Nagy A. Farid, and Atsushi Kurihara. Quantitative determination of clopidogrel active metabolite in human plasma by LC-MS/MS. *Journal of Pharmaceutical and Biomedical Analysis*, 48(4):1219–1224, 2008. ISSN 07317085. doi: 10.1016/j.jpba.2008.08.020.

[40] David S. Small, Christopher D. Payne, Prajakti Kothare, Eunice Yuen, Fanni Natanegara, Mei Teng Loh, Joseph A. Jakubowski, D. Richard Lachno, Ying G. Li, Kenneth J. Winters, Nagy A. Farid, Lan Ni, Daniel E. Salazar, Molly Tomlin, and Ronan Kelly. Pharmacodynamics and pharmacokinetics of single doses of prasugrel 30 mg and clopidogrel 300 mg in healthy Chinese and white volunteers: An open-label trial. *Clinical Therapeutics*, 32(2):365–379, 2010. ISSN 01492918. doi: 10.1016/j.clinthera.2010.02.015. URL `http://dx.doi.org/10.1016/j.clinthera.2010.02.015`.

[41] Masahiko Kobayashi, Miyuki Kajiwara, and Setsuo Hasegawa. A randomized study of the safety, tolerability, pharmacodynamics, and pharmacokinetics of clopidogrel in three different CYP2C19 genotype groups of healthy Japanese subjects. *J Atheroscler Thromb*, 22(11):1186–1196, 2015.

[42] D. J. Angiolillo, C. M. Gibson, S. Cheng, C. Ollier, O. Nicolas, L. Bergougnan, L. Perrin, F. P. Lacreta, F. Hurbin, and M. Dubar. Differential effects of omeprazole and pantoprazole on the pharmacodynamics and pharmacokinetics of clopidogrel in healthy subjects: Randomized, placebo-controlled, crossover comparison studies. *Clinical Pharmacology and Therapeutics*, 89(1):65–74, jan 2011. ISSN 00099236. doi: 10.1038/clpt.2010.219.

[43] Fabrice Hurbin, Xavier Boulenc, Nikki Daskalakis, Christine Farenc, Theresa Taylor, Damien Bonneau, Frank Lacreta, Sue Cheng, and Eric Sultan. Clopidogrel pharmacodynamics and pharmacokinetics in the fed and fasted state: A randomized crossover study of healthy men. *Journal of Clinical Pharmacology*, 52 (10):1506–1515, 2012. ISSN 00912700. doi: 10.1177/0091270011419852.

[44] Michael T. Furlong, Ishani Savant, Moucun Yuan, Laura Scott, William Mylott, Thomas Mariannino, Pathanjali Kadiyala, Vikram Roongta, and Mark E. Arnold. A validated HPLC-MS/MS assay for quantifying unstable pharmacologically active metabolites of clopidogrel in human plasma: Application to a clinical pharmacokinetic study. *Journal of Chromatography B: Analytical Technologies in the Biomedical and Life Sciences*, 926:36–41, 2013. ISSN 15700232. doi: 10.1016/j.jchromb.2013.02.031.

[45] Rupert P. Austin, Patrick Barton, Scott L. Cockroft, Mark C. Wenlock, and Robert J. Riley. The influence of nonspecific microsomal binding on apparent intrinsic clearance, and its prediction from physicochemical properties. *Drug Metabolism and Disposition*, 30(12):1497–1503, 2002. ISSN 00909556. doi: 10.1124/dmd.30.12.1497.

[46] ChemAxon Clopidogrel, Available online (accessed on 19 February 2021). URL `https://chemicalize.com/app/calculation`.

[47] Milan Remko, Anna Remková, and Ria Broer. A comparative study of molecular structure, pKa, lipophilicity, solubility, absorption and polar surface area of some antiplatelet drugs. *International Journal of Molecular Sciences*, 17(3):388, mar 2016. ISSN 14220067. doi: 10.3390/ijms17030388.

[48] A. Tornio, A. M. Filppula, O. Kailari, M. Neuvonen, T. H. Nyrönen, T. Tapaninen, P. J. Neuvonen, M. Niemi, and J. T. Backman. Glucuronidation converts clopidogrel to a strong time-dependent inhibitor of CYP2C8: A phase II metabolite as a perpetrator of drug-drug interactions. *Clinical Pharmacology and Therapeutics*, 96(4):498–507, oct 2014. ISSN 15326535. doi: 10.1038/clpt.2014.141.

[49] Fachinformation Plavix Sanofi 75 mg / 300 mg Filmtabletten, Available online (accessed on 28 September 2021). URL `https://www.fachinfo.de/suche/fi/003345`.

[50] Hao Jie Zhu, Xinwen Wang, Brian E. Gawronski, Bryan J. Brinda, Dominick J. Angiolillo, and John S. Markowitz. Carboxylesterase 1 as a determinant of clopidogrel metabolism and activations. *Journal of Pharmacology and Experimental Therapeutics*, 344(3):665–672, mar 2013. ISSN 00223565. doi: 10.1124/jpet.112.201640.

[51] Heleen J. Bouman, Edgar Schömig, Jochem W. Van Werkum, Janna Velder, Christian M. Hackeng, Christoph HirschhÄuser, Christopher Waldmann, Hans Günther Schmalz, Jurrién M. Ten Berg, and Dirk Taubert. Paraoxonase-1 is a major determinant of clopidogrel efficacy. *Nature Medicine*, 17(1):110–116, jan 2011. ISSN 10788956. doi: 10.1038/nm.2281.

[52] Miho Kazui, Yumi Nishiya, Tomoko Ishizuka, Katsunobu Hagihara, Nagy A. Farid, Osamu Okazaki, Toshihiko Ikeda, and Atsushi Kurihara. Identification of the human cytochrome P450 enzymes involved in the two oxidative steps in the bioactivation of clopidogrel to its pharmacologically active metabolite. *Drug Metabolism and Disposition*, 38(1):92–99, jan 2010. ISSN 00909556. doi: 10.1124/dmd.109.029132.

[53] Dirk Taubert, Nicolas von Beckerath, Gundula Grimberg, Andreas Lazar, Norma Jung, Tobias Goeser, Adnan Kastrati, Albert Schömig, and Edgar Schömig. Impact of P-glycoprotein on clopidogrel absorption. *Clinical Pharmacology and Therapeutics*, 80(5):486–501, nov 2006. ISSN 00099236. doi: 10.1016/j.clpt.2006.07.007.

[54] Ryosei Kawai, Michel Lemaire, Jean Louis Steimer, Armin Bruelisauer, Werner Niederberger, and Malcolm Rowland. Physiologically based pharmacokinetic study on a cyclosporin derivative, SDZ IMM 125. *Journal of Pharmacokinetics and Biopharmaceutics*, 22(5):327–365, 1994. ISSN 0090466X. doi: 10.1007/BF02353860.

[55] Open Systems Pharmacology Suite Community. Open Systems Pharmacology Suite Manual, Version 7.4 2018, Available online (accessed on 28 September 2021). URL `https://docs.open-systems-pharmacology.org/working-with-pk-sim/pk-sim-documentation`.

[56] Silvia Farfan, Marina Marcos Valdez, Octavio Fandino, Norma Sperandeo, and Sonia Faudone. Comparative dissolution and polymorphism study of clopidogrel bisulfate tablets available in argentine. *Journal of Applied Pharmaceutical Science*, 10(10):62–71, oct 2020. ISSN 22313354. doi: 10.7324/JAPS.2020.10107.

[57] Katsunobu Hagihara, Yumi Nishiya, Atsushi Kurihara, Miho Kazui, Nagy A Farid, and Toshihiko Ikeda. Comparison of human cytochrome P450 inhibition by the thienopyridines prasugrel, clopidogrel, and ticlopidine. *Drug Metab. Pharmacokinet*, 23(6):412–420, 2008.

[58] Robert L. Walsky, Angela V. Astuccio, and R. Scott Obach. Evaluation of 227 drugs for in vitro inhibition of cytochrome P450 2B6. *Journal of Clinical Pharmacology*, 46(12):1426–1438, dec 2006. ISSN 00912700. doi: 10.1177/0091270006293753.

[59] Robert L. Walsky and R. Scott Obach. A comparison of 2-Phenyl-2-(1-piperidinyl)propane (PPP), 1,1',1"-phosphinothioylidynetrisaziridine (ThioTEPA), clopidogrel, and ticlopidine as selective inactivators of human cytochrome P450 2B6. *Drug Metabolism and Disposition*, 35(11):2053–2059, nov 2007. ISSN 00909556. doi: 10.1124/dmd.107.015883.

[60] Yumi Nishiya, Katsunobu Hagihara, Takashi Ito, Masami Tajima, Shin Ichi Miura, Atsushi Kurihara, Nagy A. Farid, and Toshihiko Ikeda. Mechanism-based inhibition of human cytochrome P450 2B6 by ticlopidine, clopidogrel, and the thiolactone metabolite of prasugrel. *Drug Metabolism and Disposition*, 37(3):589–593, mar 2009. ISSN 00909556. doi: 10.1124/dmd.108.022988.

[61] Soo Kyung Bae, Shan Cao, Kyung Ah Seo, Hyunmi Kim, Min Jung Kim, Ji Hong Shon, Kwang Hyeon Liu, Hong Hao Zhou, and Jae Gook Shin. Cytochrome P450 2B6 catalyzes the formation of pharmacologically active sibutramine (N-1-[1-(4-chlorophenyl)cyclobutyl]-3-methylbutyl-N,N- dimethylamine) metabolites in human liver microsomes. *Drug Metabolism and Disposition*, 36(8):1679–1688, aug 2008. ISSN 00909556. doi: 10.1124/dmd.108.020727.

[62] Tanja Richter, Thomas E. Mürdter, Georg Heinkele, Jürgen Pleiss, Stephan Tatzel, Matthias Schwab, Michel Eichelbaum, and Ulrich M. Zanger. Potent mechanism-Based inhibition of human CYP2B6 by clopidogrel and ticlopidine. *Journal of Pharmacology and Experimental Therapeutics*, 308(1):189 – 197, sep 2004. doi: 10.1240/sav_gbm_2002_h_000185.

[63] Haoming Zhang, Hemali Amunugama, Sarah Ney, Nyemade Cooper, and Paul F. Hollenberg. Mechanism-based inactivation of human cytochrome P450 2B6 by clopidogrel: Involvement of both covalent modification of cysteinyl residue 475 and loss of heme. *Molecular Pharmacology*, 80(5):839–847, nov 2011. ISSN 0026895X. doi: 10.1124/mol.111.073783.

[64] J. S. Floyd, R. Kaspera, K. D. Marciante, N. S. Weiss, S. R. Heckbert, T. Lumley, K. L. Wiggins, B. Tamraz, P. Y. Kwok, R. A. Totah, and B. M. Psaty. A screening study of drug-drug interactions in cerivastatin users: An adverse effect of clopidogrel. *Clinical Pharmacology and Therapeutics*, 91(5): 896–904, may 2012. ISSN 00099236. doi: 10.1038/clpt.2011.295.

[65] Robert L. Walsky, Emily A. Gaman, and R. Scott Obach. Examination of 209 drugs for inhibition of cytochrome P450 2C8. *Journal of Clinical Pharmacology*, 45(1):68–78, jan 2005. ISSN 00912700. doi: 10.1177/0091270004270642.

[66] Y. Nishiya, K. Hagihara, A. Kurihara, N. Okudaira, N. A. Farid, O. Okazaki, and T. Ikeda. Comparison of mechanism-based inhibition of human cytochrome P450 2C19 by ticlopidine, clopidogrel, and prasugrel. *Xenobiotica*, 39(11):836–843, nov 2009. ISSN 00498254. doi: 10.3109/00498250903191427.

[67] Bani Tamraz, Hisayo Fukushima, Alan R. Wolfe, Rudiger Kaspera, Rheem A. Totah, James S. Floyd, Benjamin Ma, Catherine Chu, Kristin D. Marciante, Susan R. Heckbert, Bruce M. Psaty, Deanna L. Kroetz, and Pui Yan Kwok. OATP1B1-related drug-drug and drug-gene interactions as potential risk factors for cerivastatin-induced rhabdomyolysis. *Pharmacogenetics and Genomics*, 23(7):355–364, 2013. ISSN 17446880. doi: 10.1097/FPC.0b013e3283620c3b.

[68] ChemAxon Clopidogrel carboxylic acid, Available online (accessed on 19 February 2021). URL `https://chemicalize.com/app/calculation`.

[69] Shobana Ganesan, Craig Williams, Cheryl L. Maslen, and Ganesh Cherala. Clopidogrel variability: Role of plasma protein binding alterations. *British Journal of Clinical Pharmacology*, 75(6):1468–1477, jun 2013. ISSN 03065251. doi: 10.1111/bcp.12017.

[70] Helinä Kahma, Anne M. Filppula, Mikko Neuvonen, E. Katriina Tarkiainen, Aleksi Tornio, Mikko T. Holmberg, Matti K. Itkonen, Moshe Finel, Pertti J. Neuvonen, Mikko Niemi, and Janne T. Backman. Clopidogrel carboxylic acid glucuronidation is mediated mainly by UGT2B7, UGT2B4, and UGT2B17: Implications for pharmacogenetics and drug-drug interactions. *Drug Metabolism and Disposition*, 46(2): 141–150, feb 2018. ISSN 1521009X. doi: 10.1124/dmd.117.078162.

[71] ChemAxon Clopidogrel acyl glucuronide, Available online (accessed on 19 February 2021). URL `https://chemicalize.com/app/calculation`.

[72] ChemAxon 2-Oxo-clopidogrel, Available online (accessed on 19 February 2021). URL `https://chemicalize.com/app/calculation`.

[73] Ru Jun Xu, Wei Min Kong, Xiao Fei An, Jian Jun Zou, Li Liu, and Xiao Dong Liu. Physiologically-based pharmacokinetic-pharmacodynamics model characterizing CYP2C19 polymorphisms to predict clopidogrel pharmacokinetics and its anti-platelet aggregation effect following oral administration to coronary artery disease patients with or without diabetes. *Frontiers in Pharmacology*, 11, dec 2020. ISSN 16639812. doi: 10.3389/fphar.2020.593982.

[74] S. Samant, X. L. Jiang, L. A. Peletier, A. R. Shuldiner, R. B. Horenstein, J. P. Lewis, L. J. Lesko, and S. Schmidt. Identifying clinically relevant sources of variability: The clopidogrel challenge. *Clinical Pharmacology and Therapeutics*, 101(2):264–273, feb 2017. ISSN 15326535. doi: 10.1002/cpt.459.

[75] Walter Schmitt. General approach for the calculation of tissue to plasma partition coefficients. *Toxicology in Vitro*, 22(2):457–467, 2008. ISSN 08872333. doi: 10.1016/j.tiv.2007.09.010.

[76] ChemAxon Clopidogrel Thiol H4, Available online (accessed on 19 February 2021). URL `https://chemicalize.com/app/calculation`.

[77] T. Rodgers, D. Leahy, and M. Rowland. Physiologically based pharmacokinetic modeling 1: predicting the tissue distribution of moderate-to-strong bases. *Journal of pharmaceutical sciences*, 94(6):1259 – 1276, 2005.

[78] T. Rodgers and M. Rowland. Physiologically based pharmacokinetic modelling 2: predicting the tissue distribution of acids, very weak bases, neutrals and zwitterions. *Journal of pharmaceutical sciences*, 95 (6):1238 – 1257, 2006.

[79] Bai Li Song, Meng Wan, Dan Tang, Chao Sun, Yu Bing Zhu, Nyame Linda, Hong Wei Fan, and Jian Jun Zou. Effects of CYP2C19 genetic polymorphisms on the pharmacokinetic and pharmacodynamic properties of clopidogrel and its active metabolite in healthy Chinese subjects. *Clinical Therapeutics*, 40(7): 1170–1178, 2018. ISSN 1879114X. doi: 10.1016/j.clinthera.2018.06.001.

[80] Yifan Zhang, Xiaoxue Zhu, Yan Zhan, Xiaojiao Li, Cai Liu, Yunting Zhu, Hong Zhang, Haijing Wei, Yu Xia, Hongbin Sun, Yongqiang Liu, Xiaojuan Lai, Yanchun Gong, Xuefang Liu, Yongguo Li, Yanhua Ding, and Dafang Zhong. Impacts of CYP2C19 genetic polymorphisms on bioavailability and effect on platelet adhesion of vicagrel, a novel thienopyridine P2Y12 inhibitor. *British Journal of Clinical Pharmacology*, 86(9):1860–1874, sep 2020. ISSN 13652125. doi: 10.1111/bcp.14296.

[81] Eleanor J. Guest, Leon Aarons, J. Brian Houston, Amin Rostami-Hodjegan, and Aleksandra Galetin. Critique of the two-fold measure of prediction success for ratios: Application for the assessment of drug-drug interactions. *Drug Metabolism and Disposition*, 39(2):170–173, 2011. ISSN 00909556. doi: 10.1124/dmd.110.036103.

[82] Jiunn H. Lin. CYP induction-mediated drug interactions: In vitro assessment and clinical implications. *Pharmaceutical Research*, 23(6):1089–1116, 2006. ISSN 07248741. doi: 10.1007/s11095-006-0277-7.

[83] Tommy Andersson, Peter Nagy, Mohammad Niazi, Sven Nylander, Hal Galbraith, Santosh Ranjan, and Lars Wallentin. Effect of esomeprazole with/without acetylsalicylic acid, omeprazole and lansoprazole on pharmacokinetics and pharmacodynamics of clopidogrel in healthy volunteers. *American Journal of Cardiovascular Drugs*, 14(3):217–227, 2014. ISSN 1179187X. doi: 10.1007/s40256-014-0073-4.

[84] Christian Funck-Brentano, Jean Szymezak, Olivier Steichen, Dominique Ducint, Mathieu Molimard, Véronique Remones, Michel Azizi, and Pascale Gaussem. Effects of rabeprazole on the antiplatelet effects and pharmacokinetics of clopidogrel in healthy volunteers. *Archives of Cardiovascular Diseases*, 106(12): 661–671, dec 2013. ISSN 18752136. doi: 10.1016/j.acvd.2013.09.002.

[85] H. M. Judge, S. B. Patil, R. J. Buckland, J. A. Jakubowski, and Robert F. Storey. Potentiation of clopidogrel active metabolite formation by rifampicin leads to greater P2Y12 receptor blockade and inhibition of platelet aggregation after clopidogrel. *Journal of Thrombosis and Haemostasis*, 8(8):1820–1827, aug 2010. ISSN 15387933. doi: 10.1111/j.1538-7836.2010.03925.x.

[86] Miia Turpeinen, Ari Tolonen, Jouko Uusitalo, Jorma Jalonen, Olavi Pelkonen, and Kari Laine. Effect of clopidogrel and ticlopidine on cytochrome P450 2B6 activity as measured by bupropion hydroxylation. *Clinical Pharmacology and Therapeutics*, 77(6):553–559, jun 2005. ISSN 00099236. doi: 10.1016/j.clpt.2005.02.010.

[87] Matti K. Itkonen, Aleksi Tornio, Anne M. Filppula, Mikko Neuvonen, Pertti J. Neuvonen, Mikko Niemi, and Janne T. Backman. Clopidogrel but not prasugrel significantly inhibits the CYP2C8-mediated metabolism of montelukast in humans. *Clinical Pharmacology and Therapeutics*, 104(3):495–504, sep 2018. ISSN 15326535. doi: 10.1002/cpt.947.

[88] B. L. Chen, Y. Chen, J. H. Tu, Y. L. Li, W. Zhang, Q. Li, L. Fan, Z. R. Tan, D. L. Hu, D. Wang, L. S. Wang, D. S. OuYang, and Zhou H. H. Clopidogrel inhibits CYP2C19-dependent hydroxylation of omeprazole related to CYP2C19 genetic polymorphisms. *Journal of Clinical Pharmacology*, 49(574 - 581), 2009.

[89] Matti K. Itkonen, Aleksi Tornio, Mikko Neuvonen, Pertti J. Neuvonen, Mikko Niemi, and Janne T. Backman. Clopidogrel markedly increases plasma concentrations of CYP2C8 substrate pioglitazone. *Drug Metabolism and Disposition*, 44(8):1364–1371, 2016. ISSN 1521009X. doi: 10.1124/dmd.116.070375.

[90] Maurizio Fava, A. John Rush, Michael E. Thase, Anita Clayton, Stephen M. Stahl, James F. Pradko, and J. Andrew Johnston. 15 years of clinical experience with bupropion HCl. *The Primary Care Companion to The Journal of Clinical Psychiatry*, 07(03):106–113, jun 2005. ISSN 1523-5998. doi: 10.4088/PCC.v07n0305.

[91] Jennifer E. Sager, Lauren S.L. Price, and Nina Isoherranen. Stereoselective metabolism of bupropion to OH-bupropion, threohydrobupropion, erythrohydrobupropion, and 49-OH-bupropion in vitro. *Drug Metabolism and Disposition*, 44(10):1709–1719, 2016. ISSN 1521009X. doi: 10.1124/dmd.116.072363.

[92] L. M. Hesse, K. Venkatakrishnan, M. H. Court, L. L. Von Moltke, S. X. Duan, R. I. Shader, and D. J. Greenblatt. CYP2B6 mediates the in vitro hydroxylation of bupropion: Potential drug interactions with other antidepressants. *Drug Metabolism and Disposition*, 28(10):1176–1183, 2000. ISSN 00909556.

[93] Drug development and drug interactions: FDA table of substrates, inhibitors and inducers, Available online (accessed on 28 September 2021). URL `https://www.fda.gov/drugs/drug-interactions-labeling/drug-development-and-drug-interactions-table-substrates-inhibitors-and-inducers`.

[94] Anne M. Filppula, Jouko Laitila, Pertti J. Neuvonen, and Janne T. Backman. Reevaluation of the microsomal metabolism of montelukast: Major contribution by CYP2C8 at clinically relevant concentrations. *Drug Metabolism and Disposition*, 39(5):904–911, 2011. ISSN 00909556. doi: 10.1124/dmd.110.037689.

[95] T. Andersson, JO Miners, ME Veronese, and DJ Birkett. Identification of human liver cytochrome P450 isoforms mediating secondary omeprazole metabolism. *British Journal of Clinical Pharmacology*, 36(6):521–530, 1993. ISSN 13652125. doi: 10.1111/j.1365-2125.1994.tb04310.x.

[96] H Yamazaki, K Inoue, P M Shaw, W J Checovich, F P Guengerich, and T Shimada. Different contributions of cytochrome P450 2C19 and 3A4 in the oxidation of omeprazole by human liver microsomes: effects of contents of these two forms in individual human samples. *Journal of Pharmacology and Experimental Therapeutics*, 283(2):434–442, 1997.

[97] Tiina Jaakkola, Janne T Backman, Mikko Neuvonen, and Pertti J Neuvonen. Effects of gemfibrozil, itraconazole, andtheir combination on the pharmacokineticsof pioglitazone. *Clinical Pharmacology and Therapeutics*, 77(5):404–414, 2005.

[98] Tanja Busk Bidstrup, Inga Bjørnsdottir, Ulla Grove Sidelmann, Mikael Søndergård Thomsen, and Kristian Tage Hansen. CYP2C8 and CYP3A4 are the principal enzymes involved in the human in vitro biotransformation of the insulin secretagogue repaglinide. *British Journal of Clinical Pharmacology*, 56 (3):305–314, 2003. ISSN 03065251. doi: 10.1046/j.0306-5251.2003.01862.x.

[99] Akinori Nakajima, Tatsuki Fukami, Yuki Kobayashi, Akinobu Watanabe, Miki Nakajima, and Tsuyoshi Yokoi. Human arylacetamide deacetylase is responsible for deacetylation of rifamycins: Rifampicin, rifabutin, and rifapentine. *Biochemical Pharmacology*, 82(11):1747–1756, 2011. ISSN 00062952. doi: 10.1016/j.bcp.2011.08.003.

[100] Fatima Zahra Marok, Laura Maria Fuhr, Nina Hanke, Dominik Selzer, and Thorsten Lehr. Physiologically based pharmacokinetic modeling of bupropion and its metabolites in a CYP2B6 drug-drug-gene interaction network. *Pharmaceutics*, 13(3):331, 2021. doi: 10.3390/pharmaceutics.

[101] L. M. Berezhkovskiy. Volume of distribution at steady state for a linear pharmacokinetic system with peripheral elimination. *Journal of Pharmaceutical Sciences*, 93(6):1628–40, 2004.

[102] PBPK model Montelukast, Available online (accessed on 23 September 2021). URL `https://github.com/Open-Systems-Pharmacology/Montelukast-Model/releases/tag/v1.1`.

[103] Tobias Kanacher, Andreas Lindauer, Enrica Mezzalana, Ingrid Michon, Celine Veau, Jose David Gómez Mantilla, Valerie Nock, and Angèle Fleury. A physiologically-based pharmacokinetic (PBPK) model network for the prediction of CYP1A2 and CYP2C19 drug–drug–gene interactions with fluvoxamine, omeprazole, s-mephenytoin, moclobemide, tizanidine, mexiletine, ethinylestradiol, and caffeine. *Pharmaceutics*, 12(12):1191, dec 2020. ISSN 19994923. doi: 10.3390/pharmaceutics12121191.

[104] S. K. Saha, A.K. A. Chowdhury, S. C. Bachar, S. C. Das, R. H. Kuddus, and M. A. Uddin. Comparative in vitro-in vivo correlation analysis with pioglitazone tablets. *Asian Pacific Journal of Tropical Disease*, 3(6):487–91, 2013.

[105] D. Türk, N. Hanke, S. Wolf, S. Frechen, T. Eissing, T. Wendl, M. Schwab, and T. Lehr. Physiologically based pharmacokinetic models for prediction of complex CYP2C8 and OATP1B1 (SLCO1B1) drug-drug-gene interactions: a modeling network of gemfibrozil, repaglinide, pioglitazone, rifampicin, clarithromycin and itraconazole. *Clinical Pharmacokinetics*, 58(12):1595–1607, 2019.

[106] Z. Zhu, T. Yang, Y. Zhao, N. Gao, D. Leng, and P. Ding. A simple method to improve the dissolution of repaglinide and exploration of its mechanism. *Asian Journal of Pharmaceutical Sciences*, 9(4):218–25, 2014.
